# Development of Carborane‐Based Halogenated Naphthyridinone‐Analogues as Cannabinoid Receptor Type 2 (CB_2_R) Ligands

**DOI:** 10.1002/cmdc.202500251

**Published:** 2025-06-25

**Authors:** Lea Ueberham, Winnie Deuther‐Conrad, Peter Lönnecke, Aleksandr Kazimir, Evamarie Hey‐Hawkins

**Affiliations:** ^1^ Institute of Bioanalytical Chemistry Centre for Biotechnology and Biomedicine (BBZ) Faculty of Chemistry and Mineralogy Universität Leipzig Deutscher Platz 5 04103 Leipzig Germany; ^2^ Department of Experimental Neurooncological Radiopharmacy, Institute of Radiopharmaceutical Cancer Research Helmholtz‐Zentrum Dresden‐Rossendorf (HZDR) Research Site Leipzig Permoserstraße 15 04318 Leipzig Germany; ^3^ Institute for Drug Discovery Faculty of Medicine Universität Leipzig Brüderstraße 34 04103 Leipzig Germany; ^4^ Faculty of Chemistry and Chemical Engineering, Department of Chemistry Babeș‐Bolyai University Str. Arany Janos Nr. 11 RO‐400028 Cluj‐Napoca Romania

**Keywords:** carboranes, diagnostics, docking, ligand design, medicinal chemistry

## Abstract

The cannabinoid receptor type 2 (CB_2_R) is overexpressed under pathological conditions. Positron emission tomography is a noninvasive imaging technique for diagnosing disease states, but requires radiotracers with high affinity and selectivity towards CB_2_R. Currently, there is no suitable candidate routinely used in the clinics. The naphthyridinone scaffold, a promising core structure, has been modified in the past years. The modification of naphthyridinone carboxamides with carboranes as hydrophobic surrogates for purely organic moieties can lead to beneficial CB_2_R ligands with high affinity, selectivity, and metabolic stability. Herein, synthesis and characterization of eight *ortho*‐, *meta*‐, *para*‐, and *nido*‐carborane‐based naphthyridinone ligands are reported, along with their in vitro binding affinity towards human CB_1_R and CB_2_R. Additionally, the results of in silico investigations are presented. The *meta*‐ and *para*‐carborane derivatives show high affinity in the low nanomolar range and good selectivity towards CB_2_R. Only a minor influence of bromo‐ versus iodo‐substitution of the compounds is observed experimentally, while in silico data suggest a stronger influence of the halogen atom, resulting in a different order of the respective carborane isomers regarding their CB_2_R affinity. Although these compounds do not outperform the known organic derivatives, these promising carborane‐based naphthyridinones extend the portfolio of potentially useful CB_2_R ligands.

## Graphical abstract

There is no suitable radioligand for positron emission tomography imaging of CB_2_R overexpression for routine clinical usage. Herein, the synthesis, characterization, binding affinity, and docking studies of carborane‐based halo‐substituted naphthyridinone derivatives as CB_2_R ligands are presented. The *meta*‐carborane derivatives are identified as the most affine CB_2_R ligands. Only small differences in selectivity to CB_2_R are observed for the iodo‐ and bromo‐substituted *meta*‐carborane derivatives.

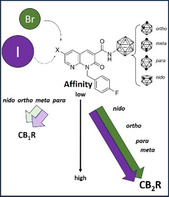

## 1 Introduction

The cannabinoid receptor type 2 (CB_2_R) is overexpressed in the brain under pathological conditions, like neurodegenerative diseases or inflammation,^[^ ^1^, ^2^, ^3^, ^4^, ^5^ ^]^ while under physiological conditions, it is only found to a low degree in the same region.^[^ ^3^, ^6^ ^]^ The CB_2_R is present in immune organs such as spleen, tonsils, and thymus.^[^ ^5^, ^7^, ^8^, ^9^, ^10^, ^11^ ^]^ The CB_1_R, however, is mainly associated with the central nervous system and therefore expressed in the brain.^[^ ^3^, ^5^, ^6^, ^12^, ^13^ ^]^ Both receptors are part of the endocannabinoid system, along with endocannabinoids, like 2‐arachidonoyl glycerol (2‐AG) or anandamide (AEA), and enzymes for synthesis and degradation of them.^[^ ^14^ ^]^ The CB_2_R receptor is of great interest due to its involvement in prominent diseases, and the search for CB_2_R‐targeted therapeutics and diagnostics is ongoing. Requirements, like a high in vivo metabolic stability and bioavailability and the need for high affinity and high selectivity towards CB_2_R, challenge the development of suitable candidates.^[^ ^12^, ^15^, ^16^, ^17^, ^18^, ^19^ ^]^

**Figure 1:**
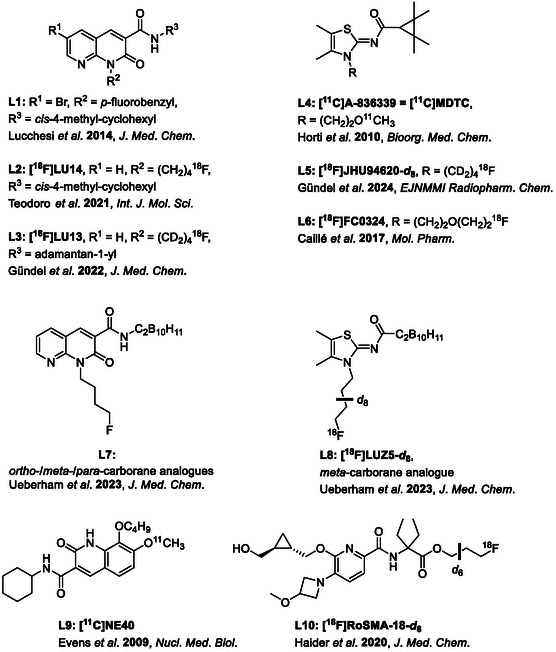
A selection of CB_2_ R (radio)ligands and their carborane‐based analogues.^[^ ^1^, ^24^, ^25^, ^26^, ^29^, ^30^, ^35^, ^38^, ^40^, ^49^, ^50^, ^52^ ^]^

Positron emission tomography (PET) is a powerful diagnostic tool for differentiating between physiological and pathological states through the measurement of CB_2_R availability.^[^ ^15^, ^20^, ^21^ ^]^ For that purpose, a radiotracer with high affinity and selectivity towards CB_2_R is needed.^[^ ^12^, ^15^, ^21^ ^]^ Additionally, a radiolabel like ^18^F or ^11^C must be present.^[^ ^20^, ^21^ ^]^ A series of promising CB_2_R‐targeting compounds, both radiolabeled or not, have been published within the last years,^[^ ^22^, ^23^ ^]^ including scaffolds of naphthyridinones,^[^ ^1^, ^24^, ^25^, ^26^ ^]^ pyridines,^[^ ^27^, ^28^ ^]^ thiazoles,^[^ ^26^, ^29^, ^30^, ^31^, ^32^, ^33^, ^34^, ^35^, ^36^, ^37^, ^38^, ^39^, ^40^ ^]^ or benzothiazoles^[^ ^41^, ^42^ ^]^ among others. Naphthyridinone, a scaffold of interest, has been modified over the past decades, e.g., by Ferrarini et al.^[^ ^43^ ^]^ Manera et al.^[^ ^44^, ^45^, ^46^ ^]^ Lucchesi et al.^[^ ^1^ ^]^ and Pascali et al.^[^ ^47^ ^]^ An additional bromo substituent in position 6 (R^1^ in **L1**, **Figure** **1**) switched the functionality of the reported compound from an agonist to an antagonist/inverse agonist.^[^ ^1^ ^]^ Additionally, the affinity and selectivity towards CB_2_R could be improved, especially for the *cis* analogue. Substituents at C6 (R^1^) and N1 (R^2^) were varied, while the 4‐methylcyclohexyl carboxamide moiety (R^3^) was kept.^[^ ^1^ ^]^ We have recently reported two 1,8‐naphthyridin‐2‐one‐based radioligands, the high affine and stereochemically pure **[** ^**18**^ **F]LU14** (Figure 1, **L2**)^[^ ^25^ ^]^ and the high affine **[** ^**18**^ **F]LU13** (Figure 1, **L3**),^[^ ^24^ ^]^ a promising radiotracer for imaging of CB_2_R overexpression in the brain. **[** ^**11**^ **C]NE40** (Figure 1, **L9**) and **[** ^**11**^ **C]MDTC** (Figure 1, **L4**) have been the first CB_2_R‐selective PET radiotracers applied in human.^[^ ^35^, ^48^ ^]^ The oxoquinoline‐based **[** ^**11**^ **C]NE40** from Evens et al.^[^ ^49^, ^50^ ^]^ has been used in PET studies in healthy humans and patients with Alzheimer's disease (AD). However, the expected increase in CB_2_R availability in AD patients could not be demonstrated.^[^ ^48^, ^51^ ^]^ Additionally, the radioligand was metabolized fast.^[^ ^51^ ^]^ **[** ^**11**^ **C]MDTC** was evaluated in healthy adults with promising results, but thorough metabolite analysis is required before the investigation of conditions with a high CB_2_R expression.^[^ ^35^ ^]^ The compound **[** ^**18**^ **F]RoSMA‐18‐** ***d***_**6**_ (Figure 1, **L10**) is forthcoming to be investigated in a first‐in‐human trial (as NCT05880563; further information is on ClinicalTrials.gov).^[^ ^52^, ^53^, ^54^ ^]^ So far, there is no suitable CB_2_R ligand or radiotracer for PET imaging in routine clinical use.^[^ ^15^, ^17^, ^55^, ^56^ ^]^

Carboranes or *closo*‐dicarba‐dodecaboranes(12) are three‐dimensional cluster compounds consisting of two CH and ten BH units.^[^ ^57^, ^58^ ^]^ Depending on the position of the two CH units, carboranes can be divided into *ortho*‐, *meta‐,* and *para*‐carborane.^[^ ^57^ ^]^ The *ortho*‐carborane is the isomer that can be deboronated most easily resulting in a *nido*‐carborane.^[^ ^57^, ^58^ ^]^ The *ortho*‐ and *nido*‐carboranes are interesting substituents, especially, in the medicinal context.^[^ ^57^, ^58^, ^59^, ^60^, ^61^, ^62^, ^63^, ^64^, ^65^, ^66^, ^67^, ^68^, ^69^, ^70^, ^71^, ^72^, ^73^, ^74^ ^]^ Besides the (fore)seen high metabolic stability,^[^ ^26^, ^59^, ^60^, ^75^, ^76^, ^77^ ^]^ other benefits are the ability to form noncovalent dihydrogen bonds^[^ ^59^ ^]^ or the chance to fine‐tune properties via substitution in a three‐dimensional manner.^[^ ^59^ ^]^ The hydrophobicity of the cluster can be favorable for the transport across cell membranes, e.g., the blood–brain barrier.^[^ ^59^, ^78^ ^]^ Often carboranes are compared to benzene, since both share the delocalization of electrons over the whole structure.^[^ ^57^, ^58^ ^]^ Regarding the van der Waals volume, adamantane is a better fit for comparison.^[^ ^57^ ^]^ Carboranes are hydrophobic substituents for multiple chemical groups.^[^ ^26^, ^59^, ^79^ ^]^ They have been used for a broad range of medicinal targets, like the estrogen receptor,^[^ ^80^ ^]^ cyclooxygenase‐2,^[^ ^79^, ^81^ ^]^ ABCG2,^[^ ^82^ ^]^ epidermal growth factor receptor,^[^ ^77^ ^]^ or vitamin D receptor.^[^ ^83^ ^]^

We have recently reported the carborane‐based naphthyridinone derivative **L7** (Figure 1) and the first carborane‐based PET radioligand **[** ^**18**^ **F]LUZ5‐** ***d***_**8**_ ((*Z*)‐*N*‐[3‐(4‐fluoro[^18^F]butyl‐*d*_8_)‐4,5‐dimethylthiazole‐2(3* H*)‐ylidene]‐(1,7‐dicarba‐*closo*‐dodecaboranyl)‐carboxamide, Figure 1, **L8**, *K*_i_CB_2_R = 0.8 nM), which binds to the CB_2_R with high affinity.^[^ ^26^ ^]^ Advantageous metabolic stability in vivo in rats and mice could be observed, which was superior to the literature‐known analogues **[** ^**18**^ **F]JHU94620** ^[^ ^31^ ^]^ and **[** ^**18**^ **F]FC0324** ^[^ ^30^ ^]^ (Figure 1, **L6**) and, especially in rat brain 30 min p.i., also superior to **[** ^**18**^ **F]JHU94620‐** ***d***_**8**_^[^ ^40^ ^]^ (Figure 1, **L5**). However, the disadvantages were a low uptake of radiotracer in the brain of healthy animals and nondisplaceable binding in the spleen.^[^ ^26^ ^]^

We here report eight carborane‐based CB_2_R ligands with a naphthyridine‐2‐one scaffold, bromine or iodine at C6 and an *ortho*‐, *meta*‐, *para*‐, or *nido*‐carborane substituent at the carboxamide group. The synthesis, full characterization, and in vitro evaluation in binding affinity assays towards CB_1_R and CB_2_R are presented and complemented by in silico studies of their binding affinity towards CB_2_R.

## 2 Results and Discussion

### 2.1 Chemical Synthesis

**Figure 2:**
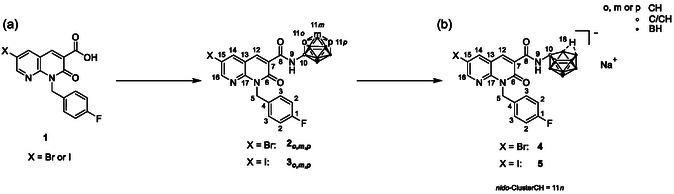
Synthesis of target compounds **2**_***o,m,p***_, **3**_***o,m,p***_, **4**, and **5**. Reagents and conditions: a) X = Br (**2**_***o***, ***m***, ***p***_): (i) SOCl_2_, toluene, reflux, 19–21 h or X = I (**3**_***o***, ***m***, ***p***_): (i) SOCl_2_, DMF cat., CH_2_Cl_2_, 0 °C → rt, 1.5 h–2 d, (ii) respective carborane‐amine C_2_B_10_H_11_‐NH_2_ (**E3**_***o***, ***m***, ***p***_), toluene, DIPEA, reflux, 19 h–3 d, and (iii) stop of reaction or continuation or heat reduction to 90 °C, 5 h–3 d. b) For **2**_***o***_ and **3**_***o***_: NaF, EtOH/H_2_O (1:1, *v*/*v*), microwave, 150 °C, 10 min.

**Figure 3:**
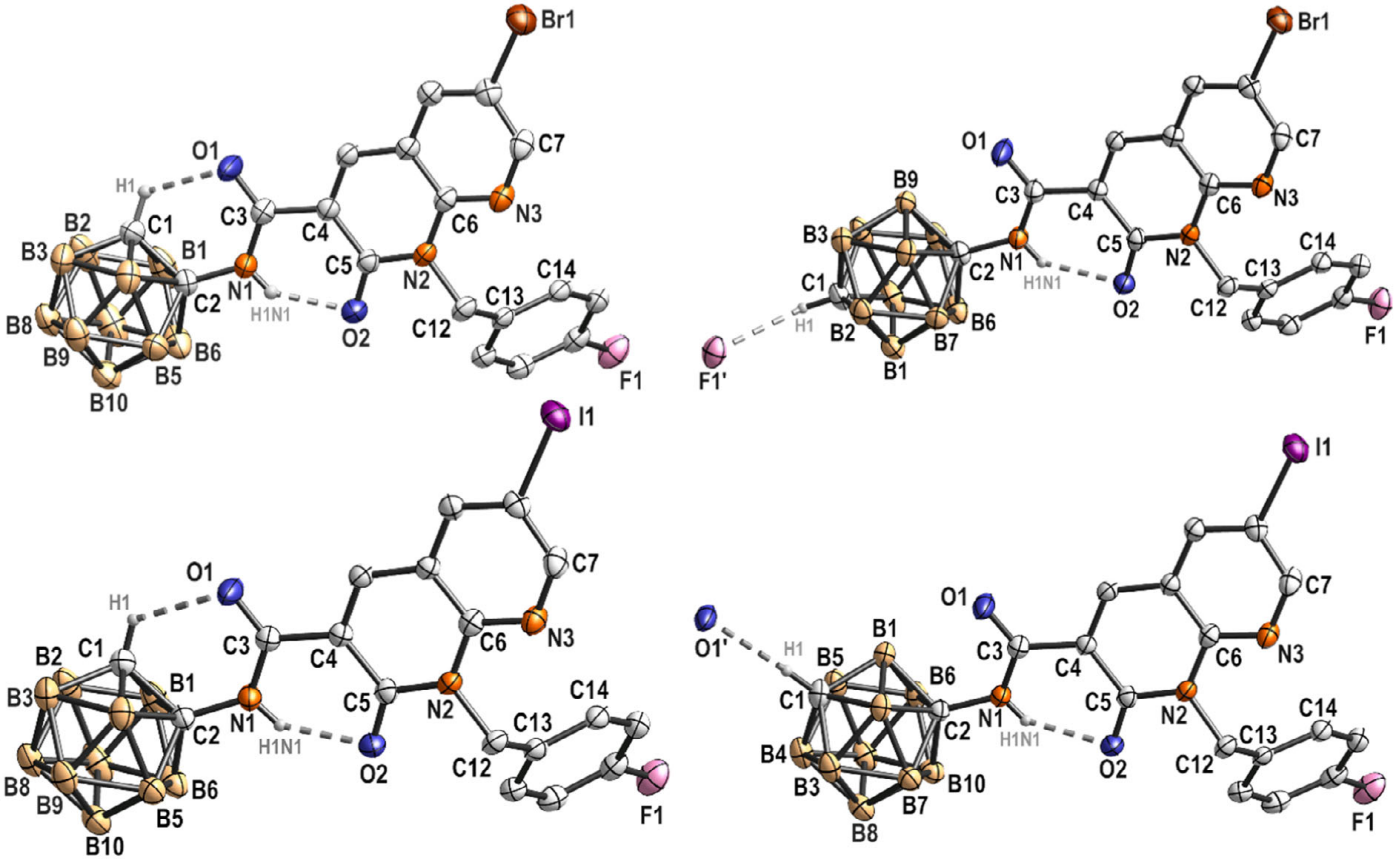
Molecular structure and labeling scheme of **2**_***o***_ (top left), **2**_***p***_ (top right), **3**_***o***_ (bottom left), and **3**_***m***_ (bottom right). Hydrogen atoms, except H atoms involved in hydrogen bonding, were omitted for clarity. Displacement ellipsoids are drawn at the 50% probability level.

The target compounds **2**_***o***, ***m***, ***p***_ and **3**_***o***, ***m***, ***p***_ (**Figure** **2**) were prepared by chlorination of compound **1** (prepared according to the literature, see Supporting Information)^[^ ^84^, ^85^ ^]^ followed by reaction with the respective amine **3**_***o***, ***m***, ***p***_ (adapted from Vázquez et al.)^[^ ^85^ ^]^ The *ortho*‐carborane derivatives **2**_***o***_ and **3**_***o***_ were deboronated with NaF and EtOH/H_2_O (1:1, *v*/*v*) in a microwave reaction to yield the respective *nido*‐carborane derivatives **4** and **5** according to Louie et al.^[^ ^86^ ^]^ The products were purified by column chromatography and fully characterized by 1D and 2D nuclear magnetic resonance (NMR) spectroscopy and electrospray ionization (ESI)‐high‐resolution mass spectrometry (HRMS). Single crystals of **2**_***o***_, **2**_***p***_, **3**_***o***_, and **3**_***m***_ suitable for X‐ray crystallography could be obtained from CDCl_3_ (Table S1 and S2, Supporting Information). The molecular structures are shown in **Figure** **3**. All compounds had a purity >95% as determined by high‐performance liquid chromatography ‐ mass spectrometry ( HPLC‐MS) measurements on an RP column (Figure S69–S81, Supporting Information) and were stable for at least 1 d, except compound **3**_***o***_, which had a purity of 93% after 6 h (Figure S82–S89, Supporting Information).

### 2.2 Biological Results

The eight target compounds **2**_***o***, ***m***, ***p***_, **3**_***o***, ***m***, ***p***_, **4**, and **5** have been investigated in in vitro binding affinity assays using crude membrane preparations obtained from Chinese hamster ovary cells stably transfected with either human CB_1_R or CB_2_R and the target‐specific radioligands **[** ^**3**^ **H]SR141716A** and **[** ^**3**^ **H]WIN55212‐2**, respectively (structures shown in Figure S90, Supporting Information).

In the previously reported series of naphthyridin‐2‐one derivatives with a fluorobutyl chain instead of a *para*‐fluoro‐benzyl group at nitrogen (**L7**, Figure 1, **Table** **1**), the *para*‐carborane compound was the derivative with the highest affinity toward CB_2_R (**L7** (***para***): *K*_i_CB_2_R = 3.9 nM). In the new series, the *meta*‐carborane derivatives are the most potent (**2**_***m***_: *K*_i_CB_2_R = 6.16 nM, **3**_***m***_: *K*_i_CB_2_R = 6.95 nM), even though the difference to the *para*‐carborane derivatives is only marginal (**2**_***p***_: *K*_i_CB_2_R = 7.71 nM, **3**_***p***_: *K*_i_CB_2_R = 7.55 nM). This corresponds to the trend previously observed for carborane‐based thiazole derivative **L8** (Figure 1).^[^ ^26^ ^]^ While the brominated analogue **2**_***m***_ and the iodinated analogue **3**_***m***_, bind with similar affinity to CB_2_R, the latter one has a lower affinity towards CB_1_R and thus a higher selectivity towards CB_2_R; but in general, there is only a slight influence of the different halogen atom at C6. The *nido*‐carborane derivatives **4** and **5** have a very low affinity towards CB_2_R. The loss of one boron atom and therefore changing properties of the carborane derivative markedly reduces the affinity for CB_2_R. The halogenated compounds **2**_***o***, ***m***, ***p***_, **3**_***o***, ***m***, ***p***_, **4**, and **5** could not reach the extremely high CB_2_R‐affinity of compound **L1** (*cis*, Figure 1, Table 1, *K*_i_CB_2_R = 0.12 nM), reported by Lucchesi et al.^[^ ^1^ ^]^ The *cis*‐4‐methyl‐cyclohexyl appears to be, in this case, the more favorable carboxamide‐substituent, then the carboranes. Nonetheless, carboranes seem to be suitable substituents for CB_2_R‐ligands, since a high affinity to CB_2_R, in the nanomolar range, can be reached.

**Table 1:** Binding affinities of target compounds **2**_***o***, ***m***, ***p***_, **3**_***o***, ***m***, ***p***_, **4**, and **5**.

Compound	*K*_i_CB_2_R [nM] meanb)	*K*_i_CB_1_R [nM] meanb)	Selectivity index (SI)e)
**2**_***o***_	29.0	97 845	3378
**2**_***m***_	6.16	1680	273
**2_*p*_**	7.71	7221	937
**3_*o*_**	15.2	3940c)	260
**3_*m*_**	6.95	6090c)	877
**3_*p*_**	7.55	5515	730
**4**	857	53 160	62
**5**	368	90 810	247
***References***			
**L1 (*cis*)** ^[^ ^1^ ^]^	0.12 ± 0.002d)	121 ± 5.32d)	1010
**L7 (*ortho*)** ^[^ ^26^ ^]^	40.8a)	30 682	752
**L7 (*meta*)** ^[^ ^26^ ^]^	35.4	3493	99
**L7 (*para*)** ^[^ ^26^ ^]^	3.9	10 140	2600

a) one value;

b) mean of two values;

c) mean of three values;

d) mean values for at least three separate experiments performed in duplicate, other testing conditions used then we applied, for details, see ref. [1];

e) SI = *K*_i_CB_1_R/*K*_i_CB_2_R.

### 2.3 Docking Results

For the docking studies, the structures of compounds **2**_***o***_, **2**_***p***_, **3**_***o***_, and **3**_***m***_ were obtained from crystallographic data, while the structures of **2** ***_m_***, **3** ***_p_***, **4**, and **5** were constructed in silico and optimized using the semiempirical PBEh‐3c method, developed by the ORCA team.^[^ ^87^ ^]^ To reproduce the charge distribution at the *nido*‐carborane clusters properly, we manually included the calculated PBEh‐3c charges in the PDBQT file of the docked structures with the *nido*‐carborane clusters (for more details see the Supporting Information). Docking simulations were performed to evaluate the binding affinity of carborane‐based compounds to the human cannabinoid receptor 2 (CB_2_R) and to determine whether specific ligand–amino acid interactions play a crucial role in receptor activation. A compound may act as an antagonist if its binding stabilizes the receptor in an inactive conformational state. In the case of human CB_2_R, the interaction with Trp258–a residue reported as a toggle switch–or conformational changes in the binding pocket that may influence Trp258 are particularly significant.^[^ ^9^ ^]^

**Figure 4:**
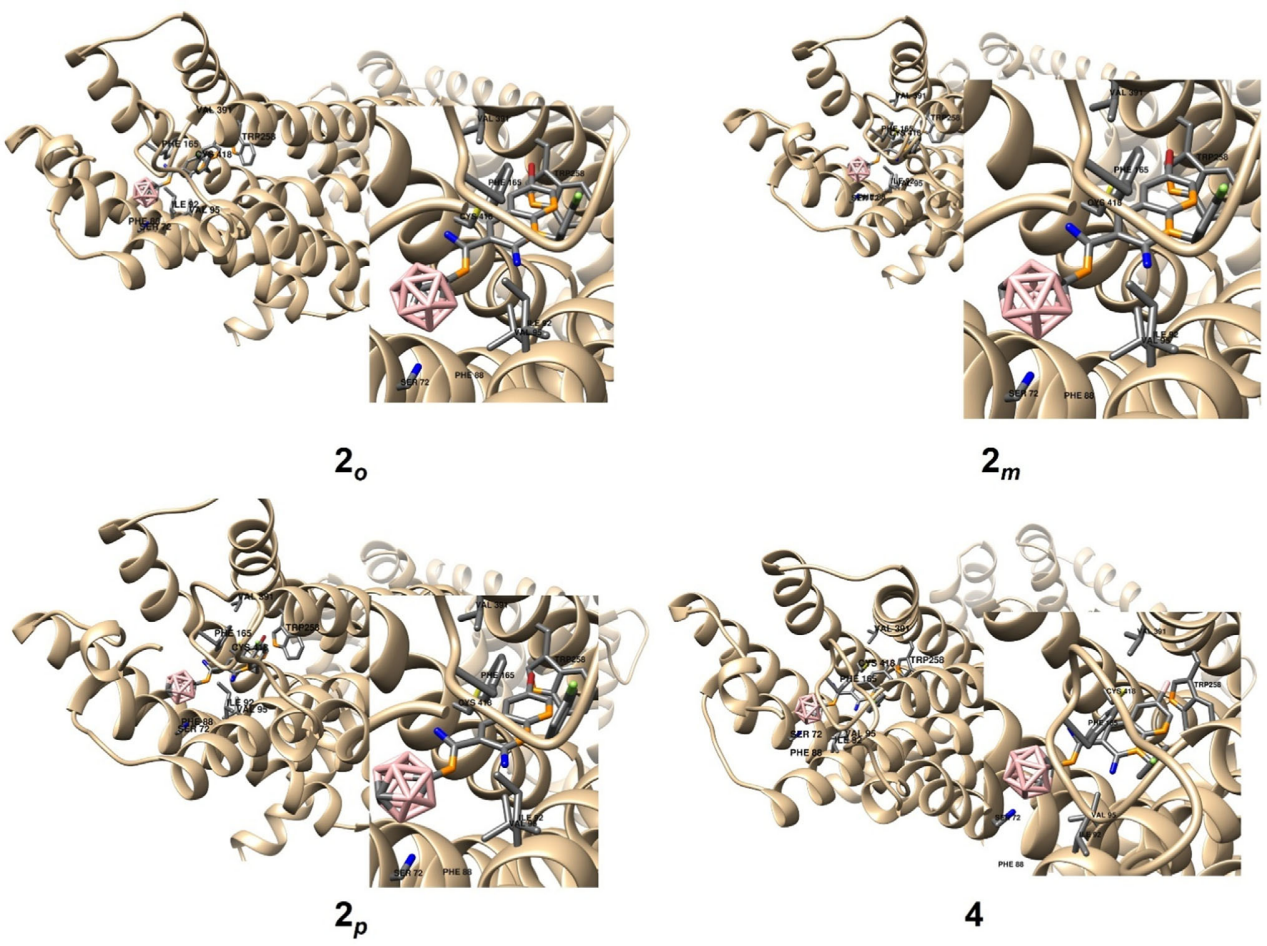
In silico investigation of the binding modes of compounds **2**_***o***_, **2**_***m***_, **2**_***p***_, and **4** based on docking studies. The highest‐ranked docked positions are shown together with amino acid residues. Hydrogen atoms have been removed for clarity.

The best‐ranked binding poses of the bromo derivatives **2**_***o***_, **2**_***m***_, **2**_***p***_, and **4** (**Table** **2**) demonstrated similar binding mode. The structures formed halogen−π noncovalent interactions between the bromine atom of the naphthyridine‐2‐one moiety and the aromatic ring of tryptophan (Trp258 labeled in our scheme is the toggle switch as Trp258 reported in the scheme of Li et al.)^[^ ^9^ ^]^ Additionally, the *nido*‐carborane derivative exhibited the lowest binding affinity among the studied compounds, with the affinity ranking following *meta* ≈ *ortho* ≈ *para* > *nido* (**Figure** **4**). The highest binding affinity for **2**_***m***_ and lowest binding affinity for **4** toward CB_2_R obtained by docking are in good agreement with the experimental data. However, the affinity of *ortho*‐carborane derivative **2**_***o***_ compared to *meta*‐carborane derivative **2**_***m***_ obtained by docking is in contrast to the experimental data, where **2**_***o***_ exhibited a significantly poorer affinity to CB_2_R.

**Figure 5:**
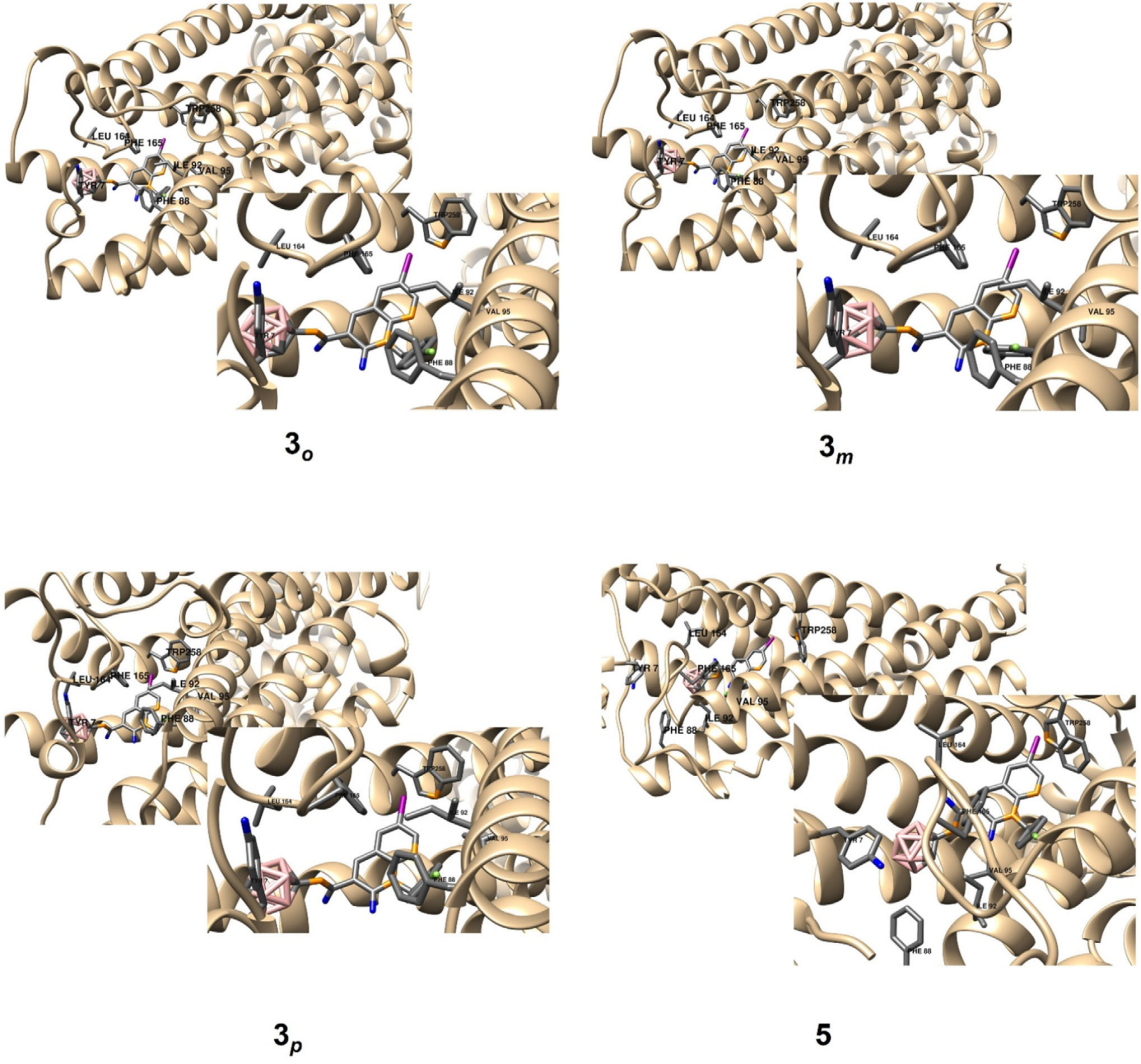
In silico investigation of the binding modes of compounds **3**_***o***_, **3**_***m***_, **3**_***p***_, and **5** based on docking studies. The highest‐ranked docked positions are shown together with amino acid residues (no interaction with Trp258 is observed for **3**_***o***_, **3**_***m***_, and **3**_***p***_). Hydrogen atoms have been removed for clarity.

Interestingly, unlike the bromo derivatives **2**_***o***_, **2**_***m***_, **2**_***p***_, and **4**, the best‐docked positions of the iodo derivatives **3**_***o***_, **3**_***m***_, **3**_***p***_, and **5** (Table 2) did not exhibit any interaction with Trp258, a key residue involved in receptor activation, despite the similarity in the halogenated position within the pyridine moiety. Although the iodine atom was oriented toward the aromatic ring of Trp258, the distance was too large to allow the formation of a noncovalent bond between **3**_***o***_, **3**_***m***_, and **3**_***p***_ and this residue. However, *nido*‐carborane derivative **5** demonstrated halogen−π interaction between iodine and Trp258. Instead, these structures were stabilized by interactions with Phe88, Val95, Ile92, Phe165, and other residues. Binding energy calculations indicated a decreasing affinity following *para* > *ortho* ≈ *meta* > *nido* (**Figure** **5**). The least affine compound was in both cases, the calculated and the experimental, the *nido* compound **5**. The *meta*‐carborane derivative **3**_***m***_ showed in the in silico derived data a binding affinity comparable to the *ortho* derivative (**3**_***o***_) and a lower binding affinity preference toward CB_2_R than the *para‐*carborane derivative (**3**_***p***_), while experimentally, the *meta*‐carborane derivative **3**_***m***_ was the most affine compound (nonetheless with only a marginal difference to the *para*‐carborane analogue). The in silico investigation indicated a stronger influence, as was observed experimentally, of the halogenated atom on the order of *closo*‐carborane isomers, regarding their binding affinity toward CB_2_R (Table 2).

**Table 2:** Binding energy values determined by docking of compounds **2**_***o***_, **2**_***m***_, **2**_***p***_, **3**_***o***_, **3**_***m***_, **3**_***p***_, **4**, and **5** into CB_2_R (PDB structure: 5zty).^[^ ^100^ ^]^

Compound	Binding energy values in [kcal/mol]
**2**_***o***_	−10.15
**2**_***m***_	−10.18
**2**_***p***_	−10.08
**3**_***o***_	−11.94
**3**_***m***_	−11.78
**3**_***p***_	−13.56
**4**	−9.88
**5**	−10.96

## 3 Conclusion

PET imaging is a useful diagnostic tool to distinguish between physiological and disease states, where the CB_2_R is overexpressed. To date, there is no CB_2_R radioligand available for routine clinical usage. The naphthyridinone scaffold was shown to be a promising core structure for the development of potent CB_2_R ligands. We have therefore synthesized eight *ortho*‐, *meta*‐, *para*‐, and *nido*‐carborane‐based CB_2_R ligands with bromine or iodine as halogen atom at C6 of the naphthyridinone moiety and evaluated them in binding affinity tests toward CB_1_R and CB_2_R. Whereas the CB_2_R affinity was markedly reduced for the *nido*‐carborane derivatives, the *meta*‐ and *para*‐carborane derivatives showed selectivity toward CB_2_R and affinities in the nanomolar range between 6.16 nM and 7.71 nM. The *meta*‐carborane derivatives were the most affine compounds, thus featuring a different trend as was previously observed for related carborane‐based naphthyridines.^[^ ^26^ ^]^ The influence of the halogen atom in position C6 was only small. The docking results indicated that the *nido*‐carborane derivatives are the least CB_2_R‐affine compounds, which is in agreement with the experimental data. However, a different order for the affinity of the *closo*‐cluster derivatives toward CB_2_R was obtained in silico compared to in vitro. Thus, the docking studies indicated a noticeable influence of the halogene atom on the order of carborane isomers.

In conclusion, the compound library of naphtyhridinone‐based CB_2_R ligands could be extended. Furthermore, we could show that the influence of different halogen atoms, here bromine and iodine, on the affinity toward CB_2_R was only very low. Carboranes are suitable substituents to modify purely organic CB_2_R ligands and might be favorable in the application as CB_2_R‐PET ligands. Future studies will now focus on the metabolic stability and in vivo behavior of the compounds presented here.

## 4 Experimental Section

4.1

4.1.1

##### General Information

Carborane‐based reactions were mostly carried out under nitrogen atmosphere, using Schlenk technique. Anhydrous toluene was dried with a solvent purification system (MBRAUN, M.Braun Inertgas‐Systeme GmbH, Garching, Germany) and stored over molecular sieves (4 or 5 Å). Diisopropylethylamine (Hünig's base) was purified and dried as described in Purification of Laboratory Chemicals.^[^ ^88^ ^]^ Compound **1** was synthesized according to the literature.^[^ ^1^, ^89^, ^90^ ^]^ All other solvents and chemicals were commercially available and used without further purification. For microwave reactions, an Initiator+ microwave from Biotage (Uppsala, Sweden) was used. Reaction progress and purification were monitored by thin‐layer chromatography (TLC) using precoated silica gel 60 F_254_ Alugram plates (Xtra SIL G) from Macherey–Nagel (Düren, Germany). Carborane‐containing TLC spots were stained with a solution of 5%–10% PdCl_2_ in methanol. Chromatography was performed in air, with silica gel (60 Å, 0.035 −0.070 mm particle diameter) or in an automated fashion with Isolera‐4 and ELSD 1080 (Biotage) with commercially available solvents. NMR spectra were recorded with a Varian MERCURYplus 300 or 400 spectrometer or with Bruker AVANCE III HD 400 or Bruker Fourier 300 spectrometer. Measurements were performed at 400 MHz (^1^H), 128 MHz (^11^B), and 76 or 101 MHz (^13^C), 377 MHz (^19^F). Chemical shifts (*δ*) are given in parts per million (ppm). ^1^H and ^13^C NMR spectra were referenced to internal deuterated solvent and ^11^B{^1^H} NMR spectra to the Ξ scale.^[^ ^91^ ^]^ The deuterated solvents CDCl_3_ and CD_3_OD were purchased from Eurisotop (Saint‐Aubin, France) with a deuteration rate of 99.80%. HRMS was conducted in either positive or negative ion mode with an ESI‐TOF microTOF instrument from Bruker Daltonik GmbH (Bremen, Germany). The target compounds were dissolved in CH_3_CN (*closo*‐carborane derivatives) or MeOH (*nido*‐carborane derivatives). The simulation of mass spectra was carried out with the Web‐based MS online tool of Scientific Instrument Service (SISweb, Palmer, MA).^[^ ^92^ ^]^ The analysis of NMR and MS data was done with MestReNova version 14.1.0.^[^ ^93^ ^]^ X‐ray analysis was performed with single crystals, obtained from CDCl_3_ at room temperature by slow evaporation of the solvent. The crystals were measured on a Gemini diffractometer (Rigaku Oxford Diffraction) with Mo K*α* radiation (*λ* = 71.073 pm) in *ω*‐scan mode. Data reduction was performed with CrysAlis Pro.^[^ ^94^ ^]^ Empirical absorption correction was performed with SCALE3 ABSPACK.^[^ ^95^ ^]^ Structure solution and anisotropic refinement were performed with SHELXT^[^ ^96^ ^]^ and SHELXL.^[^ ^97^ ^]^ All structures presented herein are isotype. For deep temperature 190 K measurements, all hydrogen atoms were located by difference Fourier maps calculated at the final stage of the structure refinement, whereas for the room temperature data most hydrogen atoms are calculated on idealized positions. With the exception of the disordered room temperature measurement of **2**_***o***_, all carborane carbon atoms could be localized with a bond length and displacement parameter analysis. The observed carborane disorder of **2**_***o***_ at room temperature is most likely a result of the relatively weak intramolecular C1‐H1.…O1 interaction (Figure 3; top left). For further details see the supporting information. Structure figures were generated with Diamond (version 4.6.8).^[^ ^98^ ^]^

##### General Procedure for the Synthesis of 2_*o,m,p*_ and 3_*o,m,p*_

For **2**_***o***, ***m***, ***p***_: Thionyl chloride was added to a solution of the respective derivative of **1** in dry toluene (10–10.5 mL) and refluxed for 19–21 h.

For **3**_***o***, ***m***, ***p***_: Thionyl chloride and cat. DMF (two droplets) was added at 0 °C to a suspension of the respective derivative of **1** in CH_2_Cl_2_ (8–9 mL) and stirred for 1.5 h to 2 d.

For **2**_***o***, ***m***, ***p***_ and **3**_***o***, ***m***, ***p***_: The solvent was removed under reduced pressure and the remaining solid was suspended in dry toluene (3–4 mL). The mixture was added to a solution of the corresponding amine **E3**_***o,m,p***_ in dry toluene (2–2.5 mL). Dry DIPEA was added directly or after 45 min to the mixture and refluxed for 19 h to 3 d. The reaction was either stopped, continued, or kept at room temperature or 90 °C, while stirring, for another 5 h to 3 d. The solvent was removed at room temperature under reduced pressure and the crude product was purified by column chromatography.

##### 6‐Bromo‐1‐(4‐fluorobenzyl)‐2‐oxo‐N‐(1,2‐*closo*‐dicarba‐dodecaborane)‐1,2‐dihydro‐1,8‐naphthyridine‐3‐carboxamide (2_*o*_)

Reagents and conditions: **1**: 151.6 mg, 0.402 mmol, 1.00 eq.; SOCl_2_: 0.3 mL, 491.4 mg, 4.13 mmol, 10 eq.; **E3**_***o***_: 66.2 mg, 0.416 mmol, 1.03 eq.; dry DIPEA: 1.0 mL, 760 mg, 5.88 mmol, 15 eq., column chromatography: *n*‐hexane/EtOAc, 83:17 *v*/*v* → 100% EtOAc. The volume of the solution containing the product was reduced. The formed crystals were isolated by filtration and washed with EtOAc and *n*‐hexane. Impure fractions were purified further by column chromatography: *n*‐hexane/EtOAc, 87:13 *v*/*v* → 25% EtOAc. The volume of the solution containing the product was reduced. The formed crystals were isolated by filtration and washed with EtOAc and *n*‐hexane. Compound **2**_***o***_ (19.6 mg, 0.038 mmol) was obtained in 9% yield as an off‐white solid. ^1^H NMR (400 MHz, CDCl_3_) *δ* 0.94–3.45 (br, 10 H, B**H**), 5.10 (s, 1 H, **11** ***o*** **‐**C**H**_**Cluster**_), 5.72 (s, 2 H, **5‐**C**H**_**2**_), 6.98 (m, 2 H, **2‐**C**H**_**ar**_), 7.49 (m, 2 H, **3‐**C**H**_**ar**_), 8.22 (d, ^3^ *J* = 2.4 Hz, 1 H, **14‐**C**H**_**ar**_), 8.74 (s, 1 H, **12‐**C**H**_**ar**_), 8.81 (d, *J* = 2.4 Hz, 1 H, **16‐**C**H**_**ar**_), 11.46 (s, 1 H, **9‐**N**H**); ^11^B{^1^H} NMR (128 MHz, CDCl_3_) *δ* – 13.8 (s, 3B), – 10.8 (s, 5B), – 7.0 (s, 1B), – 4.0 (s, 1B); ^13^C{^1^H} NMR (101 MHz, CDCl_3_) *δ* 44.5 (**5‐C**H_2_), 60.3 (**11** ***o*** **‐** **C**H_**Cluster**_), 78.5 (**10‐** **C**_**Cluster**_), 115.1 (**15‐** **C**Br), 115.4 (d, *J* = 21.4 Hz, **2‐C**H_ar_), 115.8 (**7‐/13‐** **C**_**quart**_), 122.2 (**7‐/13‐** **C**_**quart**_), 131.0 (d, *J* = 8.2 Hz, **3‐** **C**H_ar_), 131.7 (d, *J* = 3.3 Hz, **4‐** **C**_**quart**_), 140.3 (**14‐** **C**H_ar_), 142.5 (**12‐** **C**H_ar_), 148.2 (**17‐** **C**_**quart**_), 154.0 (**16‐** **C**H_ar_), 161.0 (**8‐C**O), 162.2 (**6‐C**O), 162.4 (d, *J*_CF_ = 246.9 Hz, **1‐C**F); ^19^F{^1^H} NMR (377 MHz, CDCl_3_) *δ* – 113.8; HRMS (ESI–) *m*/*z* for C_18_H_20_B_10_BrFN_3_O_2_, [M – H]^‐^ 517.1691, calcd 517.1690.

##### 6‐Bromo‐1‐(4‐fluorobenzyl)‐2‐oxo‐N‐(1,7‐*closo*‐dicarba‐dodecaborane)‐1,2‐dihydro‐1,8‐naphthyridine‐3‐carboxamide (2_*m*_)

Reagents and conditions: **1**: 148.2 mg, 0.393 mmol, 1.00 eq.; SOCl_2_: 0.3 mL, 491.4 mg, 4.13 mmol, 11 eq.; **E3**_***m***_: 65.0 mg, 0.408 mmol, 1.04 eq.; dry DIPEA: 1.0 mL, 760 mg, 5.88 mmol, 15 eq.; column chromatography: *n*‐hexane/EtOAc 87:13 *v*/*v* → 100% EtOAc and *n*‐hexane/EtOAc 83:17 *v*/*v* → 50% EtOAc. The volume of the solution containing the product was reduced, the formed crystals isolated by filtration and washed with *n*‐hexane and have been further purified by column chromatography (*n*‐hexane/EtOAc) 90:10 *v*/*v* → 100% EtOAc). Compound **2**_***m***_ (31.5 mg, 0.061 mmol) was obtained as an off‐white solid in 15% yield. ^1^H NMR (400 MHz, CDCl_3_) *δ* 0.55‐3.65 (br, 10 H, B**H**), 2.96 (s, 1 H, **11** ***m*** **‐**C**H**_**Cluster**_), 5.70 (s, 2 H, **5‐**C**H**_**2**_), 6.97 (m, 2 H, **2‐**C**H**_**ar**_), 7.46 (m, 2 H, **3‐**C**H**_**ar**_), 8.18 (d, ^3^ *J* = 2.4 Hz, 1 H, **14‐**C**H**_**ar**_), 8.77 (m, 2 H, **12‐**C**H**_**ar**_, **16‐**C**H**_**ar**_), 10.76 (s, 1 H, **9‐**N**H**); ^11^B{^1^H} NMR (128 MHz, CDCl_3_) *δ* – 15.0 (s, 4B), – 12.3 (s, 2B), – 10.7 (s, 3B), – 3.7 (s, 1B); ^13^C{^1^H} NMR (76 MHz, CDCl_3_) *δ* 44.6 (**5‐** **C**H_2_), 53.3 (**11** ***m*** **‐** **C**H_**Cluster**_), 79.9 (**10‐** **C**_**Cluster**_), 115.0 (**15‐** **C**Br), 115.5 (d, *J* = 21.4 Hz, **2‐** **C**H_ar_), 116.1 (**7‐/13‐** **C**_**quart**_), 123.2 (**7‐/13‐**C_**quart**_), 131.0 (d, *J* = 8.4 Hz, **3‐** **C**H_ar_), 132.1 (d, *J* = 3.3 Hz, **4‐** **C**_**quart**_), 140.3 (**14‐** **C**H_ar_), 142.3 (**12‐** **C**H_ar_), 148.4 (**17‐** **C**_**quart**_), 153.6 (**16‐** **C**H_ar_), 160.5 (**8‐C**O), 162.3 (**6‐C**O), 162.5 (d, *J*_CF_ = 247.6 Hz, **1‐C**F); ^19^F{^1^H} NMR (377 MHz, CDCl_3_) *δ* – 114.1; HRMS (ESI+) *m*/*z* for C_18_H_22_B_10_BrFN_3_O_2_, [M + H]^+^ 519.1856, calcd 519.1846.

##### 6‐Bromo‐1‐(4‐fluorobenzyl)‐2‐oxo‐N‐(1,12‐*closo*‐dicarba‐dodecaborane)‐1,2‐dihydro‐1,8‐naphthyridine‐3‐carboxamide (2_*p*_)

Reagents and conditions: **1**: 149.8 mg, 0.397 mmol, 1.00 eq.; SOCl_2_: 0.3 mL, 491.4 mg, 4.13 mmol, 10 eq.; **E3**_***p***_:64.8 mg, 0.407 mmol, 1.02 eq.; dry DIPEA: 1.0 mL, 760 mg, 5.88 mmol, 15 eq.; column chromatography: *n*‐hexane/EtOAc 87:13 *v*/*v* → 36% EtOAc. Compound **2**_***p***_ (60.6 mg, 0.117 mmol) was obtained as an off‐white solid in 29% yield. ^1^H NMR (400 MHz, CDCl_3_) *δ* 0.60‐3.49 (br, 10 H, B**H**), 2.72 (s, 1 H, **11** ***p*** **‐**C**H**_**Cluster**_), 5.67 (s, 2 H, **5‐**C**H**_**2**_), 6.96 (m, 2 H, **2‐**C**H**_**ar**_), 7.44 (m, 2 H, **3‐**C**H**_**ar**_), 8.14 (d, ^3^ *J* = 2.4 Hz, 1 H, **14‐**C**H**_**ar**_), 8.69 (s, 1 H, **12‐**C**H**_**ar**_), 8.74 (d, ^3^ *J* = 2.4 Hz, 1 H, **16‐**C**H**_**ar**_), 10.46 (s, 1 H, **9‐**N**H**); ^11^B{^1^H} NMR (128 MHz, CDCl_3_) *δ* – 16.4 (s, 5B), – 12.4 (s, 5B); ^13^C{^1^H} NMR (101 MHz, CDCl_3_) *δ* 44.3 (**5‐** **C**H_2_), 56.3 (**11** ***p*** **‐** **C**H_**Cluster**_), 87.6 (**10‐** **C**_**Cluster**_), 114.8 (**15‐C**Br), 115.3 (d, *J* = 21.3 Hz, **2‐** **C**H_ar_), 115.9 (**7‐/13‐** **C**_**quart**_), 123.2 (**7‐/13‐** **C**_**quart**_), 130.8 (d, *J* = 8.1 Hz, **3‐** **C**H_ar_), 132.0 (d, *J* = 3.3 Hz, **4‐** **C**_**quart**_), 140.0 (**14‐** **C**H_ar_), 142.0 (**12‐** **C**H_ar_), 148.1 (**17‐C**_**quart**_), 153.3 (**16‐** **C**H_ar_), 159.5 (**8‐C**O), 162.1 (**6‐C**O), 162.3 (d, *J*_CF_ = 246.5 Hz, **1‐C**F); ^19^F{^1^H} NMR (377 MHz, CDCl_3_) *δ* – 114.2; HRMS (ESI+) *m*/*z* for C_18_H_22_B_10_BrFN_3_O_2_, [M + H]^+^ 519.1830, calcd 519.1846.

##### 1‐(4‐Fluorobenzyl)‐6‐iodo‐2‐oxo‐N‐(1,2‐*closo*‐dicarba‐dodecaborane)‐1,2‐dihydro‐1,8‐naphthyridine‐3‐carboxamide (3_*o*_)

Reagents and conditions: **1**: 173.5 mg, 0.409 mmol, 1.00 eq.; SOCl_2_: 0.45 mL, 737.1 mg, 6.20 mmol, 15 eq.; **E3**_***o***_: 72.8 mg, 0.457 mmol, 1.12 eq.; dry DIPEA: 1.1 mL, 836 mg, 6.47 mmol, 16 eq.; column chromatography: *n*‐hexane/EtOAc 4:1 *v*/*v*. The formed crystals were isolated by decanting of solvent and washed with *n*‐hexane. The supernatant was further purified by column chromatography: *n*‐hexane/EtOAc, 87:13 *v*/*v*. Compound **3**_***o***_ (18.9 mg, 0.033 mmol) was obtained as a pale yellow to an off‐white solid in 8% yield. ^1^H NMR (400 MHz, CDCl_3_) *δ* 0.69‐3.49 (br, 10 H, B**H**), 5.10 (s, 1 H, **11** ***o*** **‐**C**H**_**Cluster**_), 5.70 (s, 2 H, **5‐**C**H**_**2**_), 6.98 (m, 2 H, **2‐**C**H**_**ar**_), 7.48 (m, 2 H, **3‐**C**H**_**ar**_), 8.38 (d, ^3^ *J* = 2.2 Hz, 1 H, **14‐**C**H**_**ar**_), 8.71 (s, 1 H, **12‐**C**H**_**ar**_), 8.94 (d, ^3^ *J* = 2.2 Hz, 1 H, **16‐**C**H**_**ar**_), 11.45 (s, 1 H, **9‐**N**H**); ^11^B{^1^H} NMR (128 MHz, CDCl_3_) *δ* – 13.8 (s, 3B), – 10.8 (s, 5B), – 7.0 (s, 1B), – 4.0 (s, 1B); ^13^C{^1^H} NMR (101 MHz, CDCl_3_) *δ* 44.4 (**5‐** **C**H_2_), 60.3 (**11** ***o*** **‐** **C**H_**Cluster**_), 78.5 (**10‐** **C**_**Cluster**_), 86.0 (**15‐C**I), 115.4 (d, *J* = 21.5 Hz, **2‐** **C**H_ar_), 116.6 (**7‐/13‐** **C**_**quart**_), 122.0 (**7‐/13‐** **C**_**quart**_), 131.0 (d, *J* = 8.1 Hz, **3‐** **C**H_ar_), 131.8 (d, *J* = 3.3 Hz, **4‐** **C**_**quart**_), 142.5 (**12‐** **C**H_ar_), 146.1 (**14‐** **C**H_ar_), 148.7 (**17‐** **C**_**quart**_), 158.6 (**16‐** **C**H_ar_), 161.0 (**8‐C**O), 162.2 (**6‐C**O), 162.4 (d, *J*_CF_ = 246.5 Hz, **1‐C**F); ^19^F{^1^H} NMR (377 MHz, CDCl_3_) *δ* – 113.9; HRMS (ESI–) *m*/*z* for C_18_H_20_B_10_FIN_3_O_2_, [M – H]^‐^ 564.1587, calcd 564.1588.

##### 1‐(4‐Fluorobenzyl)‐6‐iodo‐2‐oxo‐N‐(1,7‐*closo*‐dicarba‐dodecaborane)‐1,2‐dihydro‐1,8‐naphthyridine‐3‐carboxamide (3_*m*_)

Reagents and conditions: **1**: 153.7 mg, 0.362 mmol, 1.00 eq.; SOCl_2_: 0.40 mL, 655.2 mg, 5.51 mmol, 15 eq.; **E3**_***m***_: 64.0 mg, 0.402 mmol, 1.11 eq.; dry DIPEA: 1.0 mL, 760 mg, 5.88 mmol, 16 eq.; column chromatography: thrice *n*‐hexane/EtOAc 5:1 *v*/*v* and *n*‐hexane → *n*‐hexane/EtOAc 1:1 *v*/*v*. Compound **3**_***m***_ (45.8 mg, 0.081 mmol) was obtained as a pale yellow to off‐white solid in 22% yield. ^1^H NMR (400 MHz, CDCl_3_) *δ* 0.52‐3.36 (br, 10 H, B**H**), 2.96 (s, 1 H, **11** ***m*** **‐**C**H**_Cluster_), 5.68 (s, 2 H, **5‐**C**H**_**2**_), 6.96 (m, 2 H, **2‐**C**H**_**ar**_), 7.46 (m, 2 H, **3‐**C**H**_**ar**_), 8.34 (d, ^3^ *J* = 2.2 Hz, 1 H, **14‐**C**H**_**ar**_), 8.73 (s, 1 H, **12‐**C**H**_**ar**_), 8.90 (d, ^3^ *J* = 2.2 Hz, 1 H, **16‐**C**H**_**ar**_), 10.76 (s, 1 H, **9‐**N**H**); ^11^B{^1^H} NMR (128 MHz, CDCl_3_) *δ* – 15.2 (s, 6B), – 12.4 (s, 2B), – 10.9 (s, 1B), – 3.9 (s, 1B); ^13^C{^1^H} NMR (101 MHz, CDCl_3_) *δ* 44.4 (**5‐** **C**H_2_), 53.3 (**11** ***m*** **‐** **C**H_**Cluster**_), 79.9 (**10‐** **C**_**Cluster**_), 85.9 (**15‐C**I), 115.5 (d, *J* = 21.4 Hz, **2‐C**H_ar_), 116.8 (**7‐/13‐** **C**_**quart**_), 122.9 (**7‐/13‐** **C**_**quart**_), 131.0 (d, *J* = 8.5 Hz, **3‐** **C**H_ar_), 132.1 (d, *J* = 3.3 Hz, **4‐** **C**_**quart**_), 142.2 (**12‐** **C**H_ar_), 146.0 (**14‐** **C**H_ar_), 148.8 (**17‐**C_**quart**_), 158.3 (**16‐C**H_ar_), 160.5 (**8‐C**O), 162.3 (**6‐C**O), 162.5 (d, *J*_CF_ = 246.5 Hz, **1‐C**F); ^19^F{^1^H} NMR (377 MHz, CDCl_3_) *δ* – 114.1; HRMS (ESI+) *m*/*z* for C_18_H_22_B_10_FIN_3_O_2_, [M + H]^+^ 566.1722, calcd 566.1744.

##### 1‐(4‐Fluorobenzyl)‐6‐iodo‐2‐oxo‐N‐(1,12‐*closo*‐dicarba‐dodecaborane)‐1,2‐dihydro‐1,8‐naphthyridine‐3‐carboxamide (3_*p*_)

Reagents and conditions: **1** (a mixture of acid **1** and the already chlorinated derivative was used in an unknown ratio; calculations therefore are based on **1** = 100%): 83.2 mg, 0.432 mmol, 1.00 eq.; SOCl_2_: 0.46 mL, 753.5 mg, 6.33 mmol, 15 eq.; **E3**_***p***_: 74.7 mg, 0.469 mmol, 1.09 eq.; dry DIPEA: 1.2 mL, 912 mg, 7.06 mmol, 16 eq.; column chromatography: *n*‐hexane/EtOAc 7:1 *v*/*v*. The formed crystals have been isolated by decanting of solvent and washed with *n*‐hexane. For further purification, column chromatography was performed with *n*‐hexane/EtOAc 87:13 *v*/*v* → 80:20 *v*/*v* for one part of fractions and with *n*‐hexane → *n*‐hexane/EtOAc 80:20 *v*/*v* for another part. The formed crystals have been treated as described before. Compound **3**_***p***_ (76.7 mg, 0.136 mmol) was obtained in 31% yield as an off‐white solid. ^1^H NMR (400 MHz, CDCl_3_) *δ* 0.61‐3.48 (br, 10 H, B**H**), 2.71 (s, 1 H, **11** ***p*** **‐**C**H**_Cluster_), 5.65 (s, 2 H, **5‐**C**H**_**2**_), 6.96 (m, 2 H, **2‐**C**H**_**ar**_), 7.43 (m, 2 H, **3‐**C**H**_**ar**_), 8.30 (d, ^3^ *J* = 2.2 Hz, 1 H, **14‐**C**H**_**ar**_), 8.66 (s, 1 H, **12‐**C**H**_**ar**_), 8.87 (d, ^3^ *J* = 2.2 Hz, 1 H, **16‐**C**H**_**ar**_), 10.45 (s, 1 H, **9‐**N**H**); ^11^B{^1^H} NMR (128 MHz, CDCl_3_) *δ* – 16.4 (s, 5B), – 12.4 (s, 5B); ^13^C{^1^H} NMR (101 MHz, CDCl_3_) *δ* 44.4 (**5‐** **C**H_2_), 56.5 (**11** ***p*** **‐** **C**H_**Cluster**_), 85.8 (**15‐C**I), 87.8 (**10‐** **C**_**Cluster**_), 115.5 (d, *J* = 21.2 Hz, **2‐** **C**H_ar_), 116.8 (**7‐/13‐** **C**_**quart**_), 123.2 (**7‐/13‐** **C**_**quart**_), 130.9 (d, *J* = 8.2 Hz, **3‐** **C**H_ar_), 132.2 (d, *J* = 3.2 Hz, **4‐** **C**_**quart**_), 142.1 (**12‐** **C**H_ar_), 146.0 (**14‐** **C**H_ar_), 148.7 (**17‐** **C**_**quart**_), 158.2 (**16‐** **C**H_ar_), 159.7 (**8‐C**O), 162.3 (**6‐C**O), 162.4 (d, *J*_CF_ = 246.5 Hz, **1‐** **C**F); ^19^ F{^1^H} NMR (377 MHz, CDCl_3_) *δ* – 114.2; HRMS (ESI+) *m*/*z* for C_18_H_22_B_10_FIN_3_O_2_, [M + H]^+^ 566.1725, calcd 566.1744.

##### General Procedure for *nido*‐Carborane Synthesis

A microwave vial was filled with the respective *ortho*‐carborane analogue **2**_***o***_ or **3**_***o***_, NaF, and a mixture of degassed EtOH/H_2_O (3.0 mL, 1:1, *v*/*v*). The suspension was stirred for 1 min at room temperature and 10 min at 150 °C in the microwave (780 rpm). The solvent was removed under reduced pressure and the crude product was purified by column chromatography.

##### Sodium 6‐Bromo‐1‐(4‐fluorobenzyl)‐2‐oxo‐N‐(7,8‐*nido*‐dicarba‐dodecahydroundecaborate(–1))‐1,2‐dihydro‐1,8‐naphthyridine‐3‐carboxamide (4, racemate)

Reagents and conditions: **2**_***o***_: 39.1 mg, 0.075 mmol, 1.00 eq.; NaF: 31.7 mg, 0.755 mmol, 10 eq.; column chromatography: *n*‐hexane/EtOAc 3:1 *v*/*v* → 100% EtOAc → CH_2_Cl_2_/MeOH 9:1 and *n*‐hexane/EtOAc 1:1 *v*/*v* → 100% EtOAc. The stationary phase was washed with MeOH after each column, filtered and the solvent was removed under reduced pressure. Compound **4** (36.5 mg, 0.069 mmol) was obtained as a yellow solid in 91% yield. ^1^H NMR (400 MHz, CD_3_OD) *δ* – 2.17 (d, *J* = 65.5 Hz, 1 H, **18‐μ‐H**), – 0.59–2.92 (br, 9 H, B**H**), 2.00 (s, 1 H, **11** ***n*** **‐**C**H**_Cluster_), 5.70 (s, 2 H, **5‐**C**H**_**2**_), 6.98 (t, ^3^ *J* = 8.7 Hz, 2 H, **2‐**C**H**_**ar**_), 7.44 (m, 2 H, **3‐**C**H**_**ar**_), 8.50 (m, 1 H, **14‐**C**H**_**ar**_), 8.76 (s, 1 H, **12‐**C**H**_**ar**_), 8.78 (m, 1 H, **16‐**C**H**_**ar**_); ^11^B{^1^H} NMR (128 MHz, CD_3_OD) *δ* – 38.9 (s, 1B), – 34.5 (s, 1B), – 24.8 (s, 1B), – 20.0 (s, 3B), – 15.2 (s, 1B), – 11.6 (s, 2B); ^13^C{^1^H} NMR (101 MHz, CD_3_OD) *δ* 45.2 (**5‐** **C**H_2_), 115.4 (**15‐C**Br), 116.0 (d, *J* = 21.7 Hz, **2‐** **C**H_ar_), 117.6 (**7‐/13‐** **C**_**quart**_), 125.1 (**7‐/13‐** **C**_**quart**_), 131.5 (d, *J* = 8.3 Hz, **3‐** **C**H_ar_), 134.3 (d, *J* = 3.0 Hz, **4‐** **C**_**quart**_), 141.8 (**14‐** **C**H_ar_), 142.3 (**12‐** **C**H_ar_), 149.6 (**17‐** **C**_**quart**_), 154.0 (**16‐** **C**H_ar_), 163.4 (**6‐C**O), 165.2 (**8‐C**O); ^19^ F{^1^H} NMR (377 MHz, CD_3_OD) *δ* – 118.0; HRMS (ESI–) *m*/*z* for C_18_H_21_B_9_BrFN_3_O_2_, [M – Na]^‐^ 507.1697, calcd 507.1675.

##### Sodium 1‐(4‐Fluorobenzyl)‐6‐iodo‐2‐oxo‐N‐(7,8‐*nido*‐dicarba‐dodecahydroundecaborate(–1))‐1,2‐dihydro‐1,8‐naphthyridine‐3‐carboxamide (5, racemate)

Reagents and conditions: **3**_***o***_: 16.8 mg, 0.030 mmol, 1.00 eq.; NaF: 28.9 mg, 0.688 mmol, 23 eq.; column chromatography: *n*‐hexane/EtOAc 1:1 *v*/*v* → 100% EtOAc. The stationary phase was washed with MeOH, filtered and the solvent was removed under reduced pressure. Compound **5** was obtained quantitatively as a yellow solid. ^1^H NMR (400 MHz, CD_3_OD) *δ* – 2.15 (d, *J* = 63.1 Hz, 1 H, **18‐μ‐H**), – 0.62–2.92 (br, 9 H, B**H**), 2.00 (s, 1 H, **11** ***n*** **‐**C**H**_Cluster_), 5.68 (s, 2 H, **5‐**C**H**_**2**_), 6.98 (m, 2 H, **2‐**C**H**_**ar**_), 7.44 (m, 2 H, **3‐**C**H**_**ar**_), 8.63 (d, ^3^ *J* = 2.2 Hz, 1 H, **14‐**C**H**_**ar**_), 8.73 (s, 1 H, **12‐**C**H**_**ar**_), 8.90 (d, ^3^ *J* = 2.2 Hz, 1 H, **16‐**C**H**_**ar**_); ^11^B{^1^H} NMR (128 MHz, CD_3_OD) *δ* – 38.9 (s, 1B), – 34.5 (s, 1B), –24.8 (s, 1B), – 20.0 (s, 3B), –15.1 (s, 1B), – 11.6 (s, 2B); ^13^C{^1^H} NMR (101 MHz, CD_3_OD) *δ* 45.0 (**5‐** **C**H_2_), 86.2 (**15‐C**I), 116.0 (d, *J* = 21.7 Hz, **2‐** **C**H_ar_), 118.2 (**7‐/13‐** **C**_**quart**_), 124.8 (**7‐/13‐** **C**_**quart**_), 131.5 (d, *J* = 8.1 Hz, **3‐** **C**H_ar_), 134.3 (*d*, *J* = 3.2 Hz, **4‐** **C**_**quart**_), 142.3 (**12‐** **C**H_ar_), 147.6 (**14‐** **C**H_ar_), 149.9 (**17‐** **C**_**quart**_), 158.5 (**16‐** **C**H_ar_), 163.4 (**6‐C**O), 163.5 (d, *J*_CF_ = 245.2 Hz, **1‐** **C**F), 165.2 (**8‐C**O); ^19^ F{^1^H} NMR (377 MHz, CD_3_OD) *δ* – 118.0; HRMS (ESI–) *m*/*z* for C_18_H_21_B_9_FIN_3_O_2_, [M – Na]^‐^ 555.1538, calcd 555.1537.

##### Purity Determination

Compounds **2**_***o***, ***m***, ***p***_ and **3**_***o***, ***m***, ***p***_ were dissolved in CH_3_CN and compounds **4** and **5** in MeOH. The purity was determined with an HPLC‐UV‐MS system (UltiMate 3000 UHPLC System from Thermo Scientific, Germering, Germany, DAD detector: DAD‐3000RS, coupled to MSQ Plus single quadrupole mass spectrometer from Thermo Scientific, Austin, TX), as described in a previous publication.^[^ ^79^ ^]^ The column used was a Poroshell 120 EC‐C18 column (100 mm × 3 mm, 2.7 μm) from Agilent Technologies (Waldbronn, Germany) and the elution system consisted of eluent A: LC‐MS grade water + 0.1% formic acid, and eluent B: CH_3_CN + 0.1% formic acid. The flow was set to 0.7 mL/min and the gradient used for **2**_***o***, ***m***, ***p***_ and **3**_***o***, ***m***, ***p***_ was eluent B: 40% (0–1.5 min), 40%–100% (1.5–10 min), 100% (10–15 min), 40% (15–20 min). For compounds **4** and **5**, the gradient changed at 10 min, eluent B: 100% (10–12 min), 40% (12–15 min). Prior to each measurement, a blank measurement with the respective solvent was performed. The target compounds had a purity of >95% (Figure S69–S81, Supporting Information).

##### Stability Measurements

The stability was determined analogously to the purity determination and as described in a previous publication.^[^ ^79^ ^]^ Instead of CH_3_CN or MeOH, a solvent mixture of DMSO/H_2_O (1:1, *v*/*v*) was used for all samples, as well as the blank measurements. Measurements were conducted directly after solvent addition and up to three days (Figure S82–S89, Supporting Information).

##### Binding Affinity

The affinity assays have been performed with membrane homogenates from Chinese hamster ovary cells (CHO) stably transfected either with human CB_2_R or CB_1_R. Already published protocols have been used.^[^ ^99^ ^]^

## Conflict of Interest

The authors declare no conflict of interest.

## Supporting information

Supplementary Material

## Data Availability

The data that support the findings of this study are available in the supplementary material of this article.
