# Supplementary material for: Development of Carborane‐Based Halogenated Naphthyridinone‐Analogues as Cannabinoid Receptor Type 2 (CB2R) Ligands
Source: ChemMedChem. 2025 Jun 25;20(17):e202500251. doi: 10.1002/cmdc.202500251 (PMC12444839; doi:10.1002/cmdc.202500251)
Supplement: Supplementary file 1 — Supplementary Material [file CMDC-20-e202500251-s001.pdf]

Supporting Information

Development of Carborane-Based Halogenated Naphthyridinone-Analogues as Cannabinoid Receptor Type 2 (CB_2R) Ligands

Lea Ueberham,[a] Winnie Deuther-Conrad,[b] Peter Lönnecke,[a] Aleksandr Kazimir[c] and Evamarie Hey-Hawkins*[a, d]

[a] L. Ueberham, Dr. P. Lönnecke, Prof. Dr. Dr. h.c. mult. E. Hey-Hawkins
Centre for Biotechnology and Biomedicine (BBZ)
Faculty of Chemistry and Mineralogy
Institute of Bioanalytical Chemistry
Universität Leipzig
Deutscher Platz 5, 04103 Leipzig, Germany
E-mail: hey@uni-leipzig.de

[b] Dr. W. Deuther-Conrad
Department of Experimental Neurooncological Radiopharmacy
Institute of Radiopharmaceutical Cancer Research
Helmholtz-Zentrum Dresden-Rossendorf (HZDR),
Research Site Leipzig
Permoserstraße 15, 04318 Leipzig, Germany

[c] Dr. A. Kazimir
Institute for Drug Discovery
Faculty of Medicine
Universität Leipzig
Brüderstraße 34, 04103 Leipzig, Germany

[d] Prof. Dr. Dr. h.c. mult. E. Hey-Hawkins
Faculty of Chemistry and Chemical Engineering
Department of Chemistry
Babeș-Bolyai University
Str. Arany Janos Nr. 11
RO-400028 Cluj-Napoca, Romania

Contents

1 Experimental Section – Synthesis of compounds **1** and **E3**$_{o,m,p}$ 2

2 NMR spectra of compounds **2**$_{o,m,p}$, **3**$_{o,m,p}$, **4**, and **5** 4

3 HR-ESI Mass Spectra of Compounds **2**$_{o,m,p}$, **3**$_{o,m,p}$, **4**, and **5** 38

4 Determination of HPLC Purity of Compounds **2**$_{o,m,p}$, **3**$_{o,m,p}$, **4**, and **5** 42

5 Determination of the Stability of Compounds **2**$_{o,m,p}$, **3**$_{o,m,p}$, **4**, and **5** by HPLC 49

6 X-ray Crystallography Data of Compounds **2**$_o$, **2**$_p$, **3**$_o$, and **3**$_m$ 54

7 Docking Studies of Compounds **2**$_{o,m,p}$, **3**$_{o,m,p}$, **4**, and **5** 56

8 Chemical Structures of WIN55212-2 and SR141716A 57

1 Experimental Section – Synthesis of compounds 1 and E3$_{o,m,p}$

The synthesis of compound **1** starts with the literature-known halogenation of aldehyde **E4** with bromine or iodine.[1, 89] The work-up for the iodination was as following: sodium thiosulfate pentahydrate was dissolved in water (10%-aqueous solution) and sodium hydroxide, until pH 7 was reached, were added to the reaction mixture. After extraction with EtOAc (four times), drying over MgSO4 and filtration, the semi-crude was purified by column chromatography (*n*-hexane/EtOAc, 4:1 $\rightarrow$ 1:1, (*v*/*v*)). In the second step, a Knoevenagel condensation was performed yielding **E6**,[1] followed by the alkylation of N1 with *para*-fluorobenzyl chloride as published by Lucchesi *et al*.[1] The basic ester hydrolysis of **E7** with $LiOH \cdot H_2O$ in THF/MeOH/H_2O (2:1:1, *v*/*v*/*v*) at 60 °C[90] led to compound **1**. The synthesis of the aminocarboranes **E3$_{o,m,p}$** has been reported for the *ortho*-carborane derivative by Nie *et al*.[100] and for the *meta*- and *para*-carborane derivatives by Scholz *et al*.[101] and Choi and Byun[102].

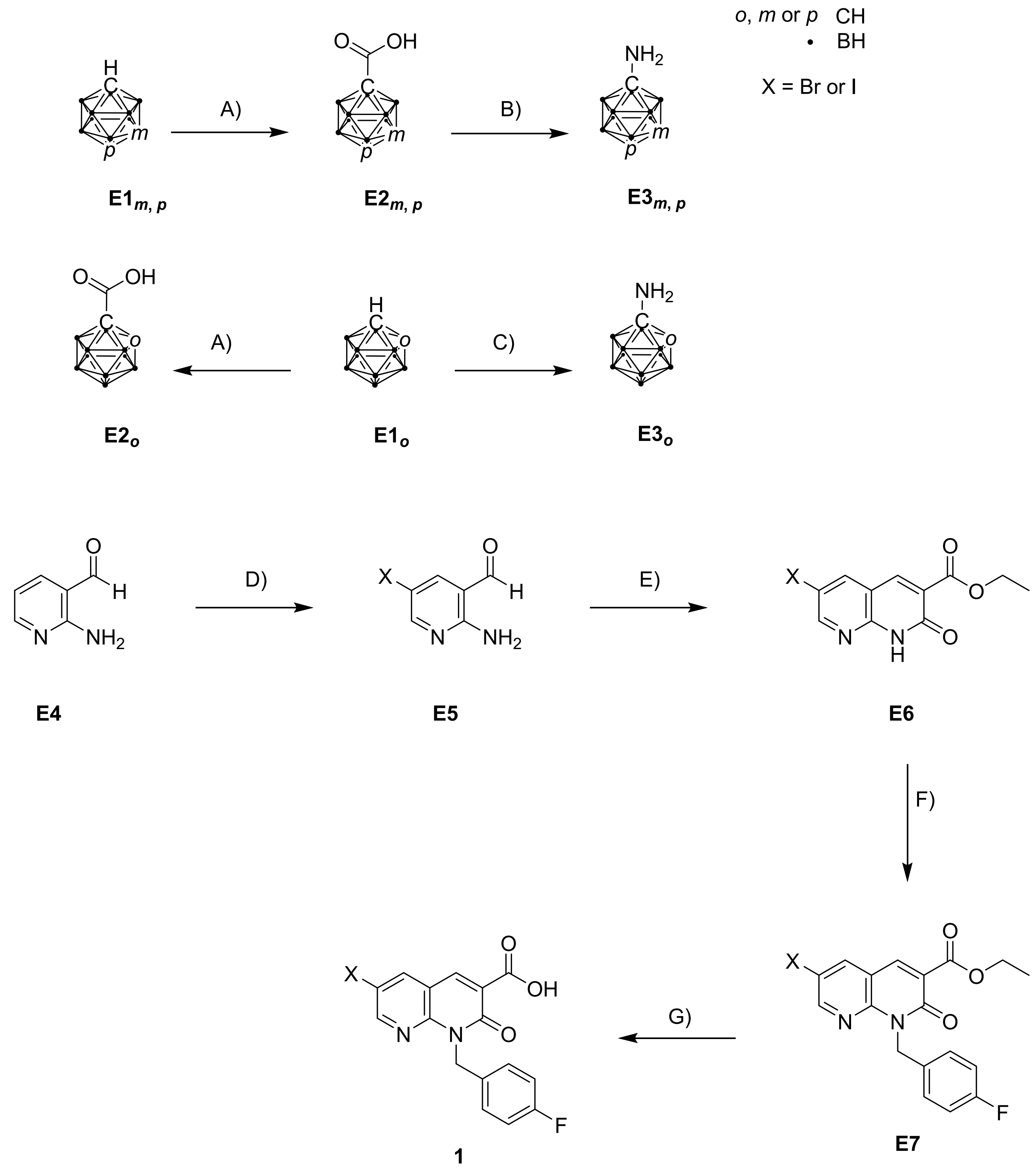

Figure S1. Synthesis of target compounds $\mathbf{2}_{o,m,p}$, $\mathbf{3}_{o,m,p}$, **4**, and **5**. Reagents and conditions: for $\mathbf{E3}_{m,p}$: (A) (i) *n*-butyllithium,Et_2O, CO_2, –78°C → RT, 16-21 h (ii) HCl and B) (i) 4-DMAP, diphenylphosphoryl azide, NEt_3, *t*ert-BuOH, reflux, 17 h (ii) TFA, CH_2Cl_2, RT, 1 d; for $\mathbf{E3}_o$: C) *n*-butyllithium, benzyl azide, Et_2O, RT, 3h (ii) glacial acetic acid, 90°C, 2h; D) for X = Br: Br_2, AcOH, RT, 1-5 d; for X = I: HIO_4, I_2, $CH_3COOH/H_2O/H_2SO_4$ (117:5.8:1, *v*/*v*/*v*), 80°C, 19.5 h ; E) diethylmalonate, piperidine, EtOH, reflux, 18-21.5 h, F) *para*-fluorobenzyl chloride, Cs_2CO_3, DMF, 50-61°C, 18.5 h; G) (i) $LiOH*H_2O$, THF/MeOH/H_2O, 2:1:1 (*v*/*v*/*v*), 60°C, 2.5-3 h, (ii) HCl.

2 NMR spectra of compounds $2_{o,m,p}$, $3_{o,m,p}$, 4, and 5

Compound **2_o**

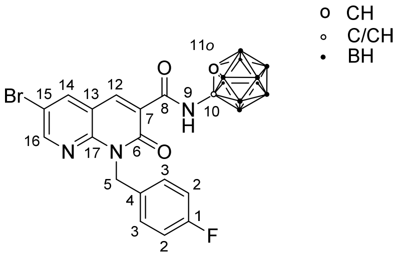

2_o

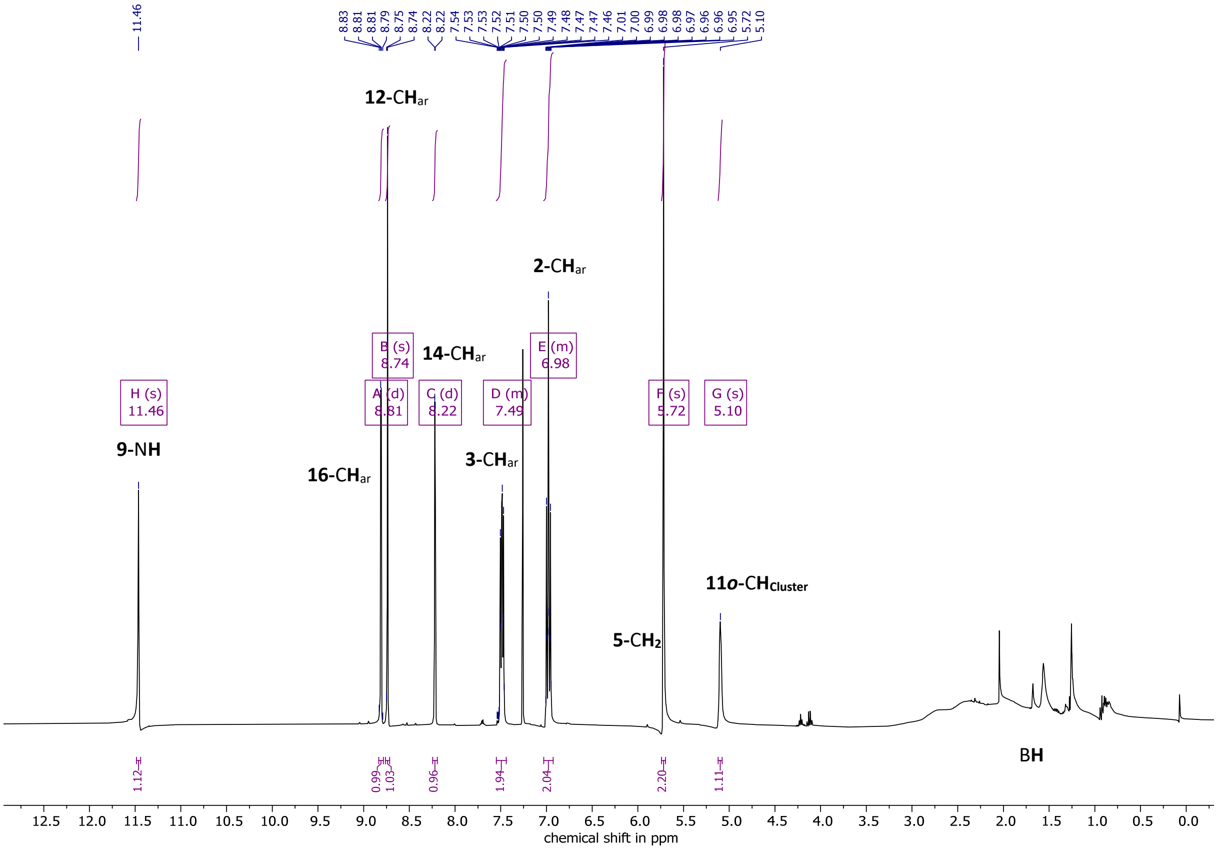

Figure S2. ^1H NMR spectrum of compound **2_o** in $CDCl_3$.

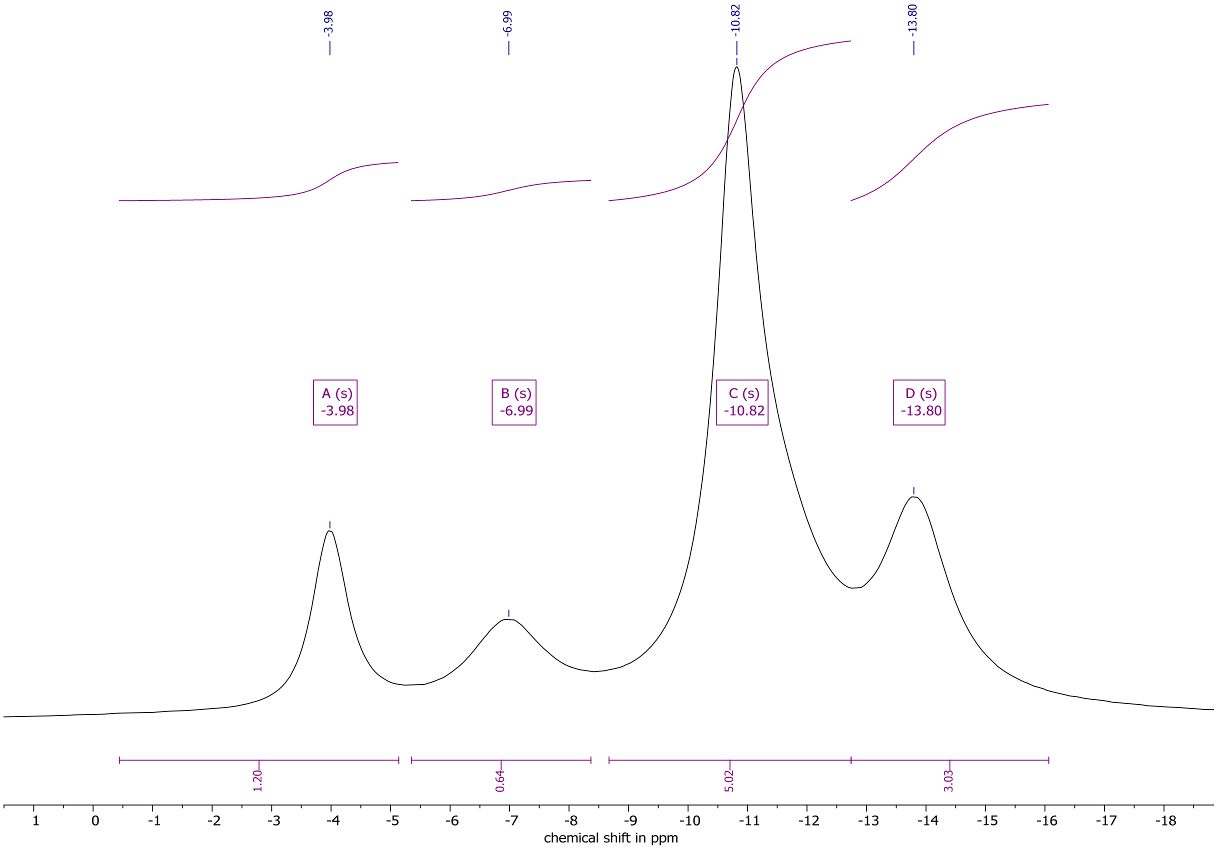

Figure S3. $^{11}B\{^{1}H\}$ NMR spectrum of compound **2*o*** in $CDCl_3$.

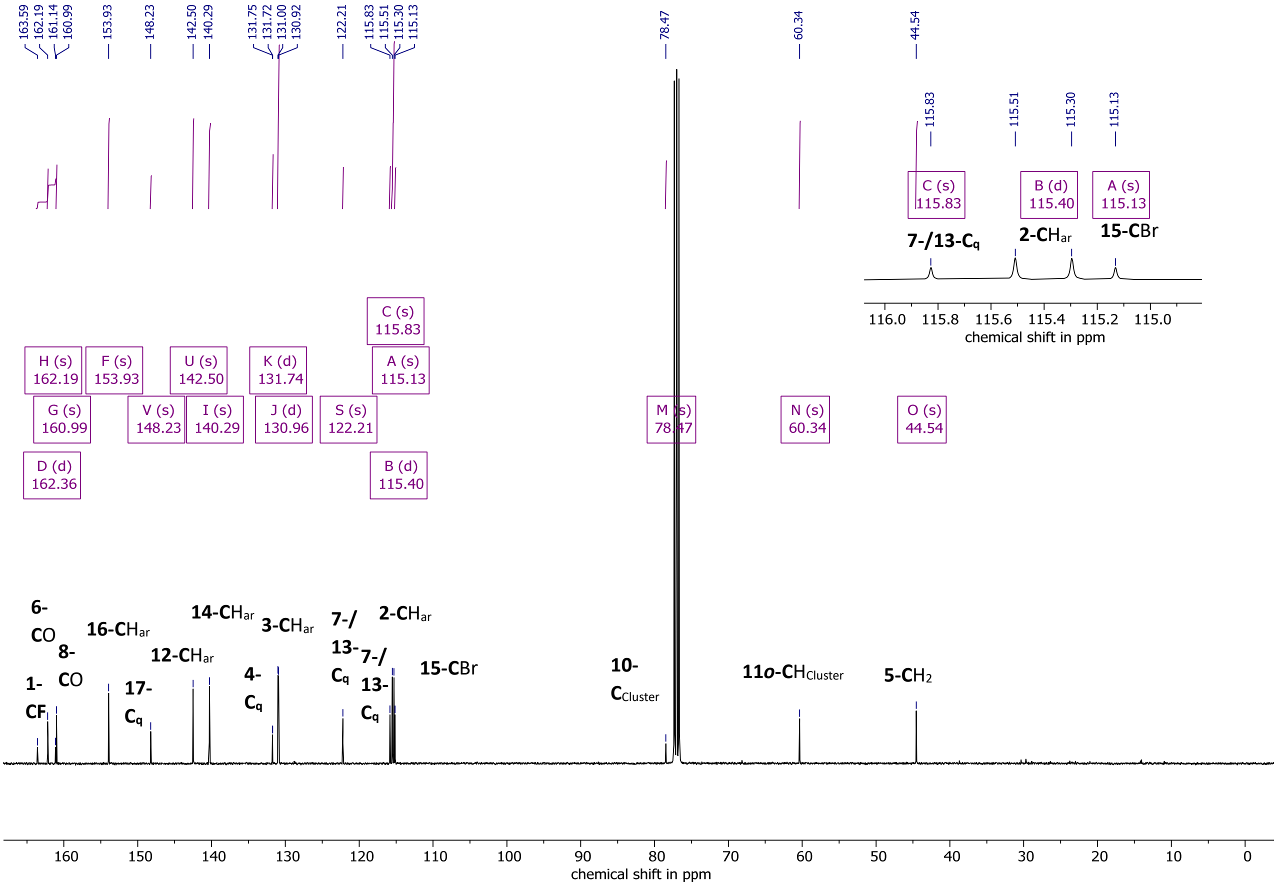

Figure S4. $^{13}C\{^{1}H\}$ NMR spectrum of compound $\mathbf{2}_{\boldsymbol{o}}$ in $CDCl_3$.

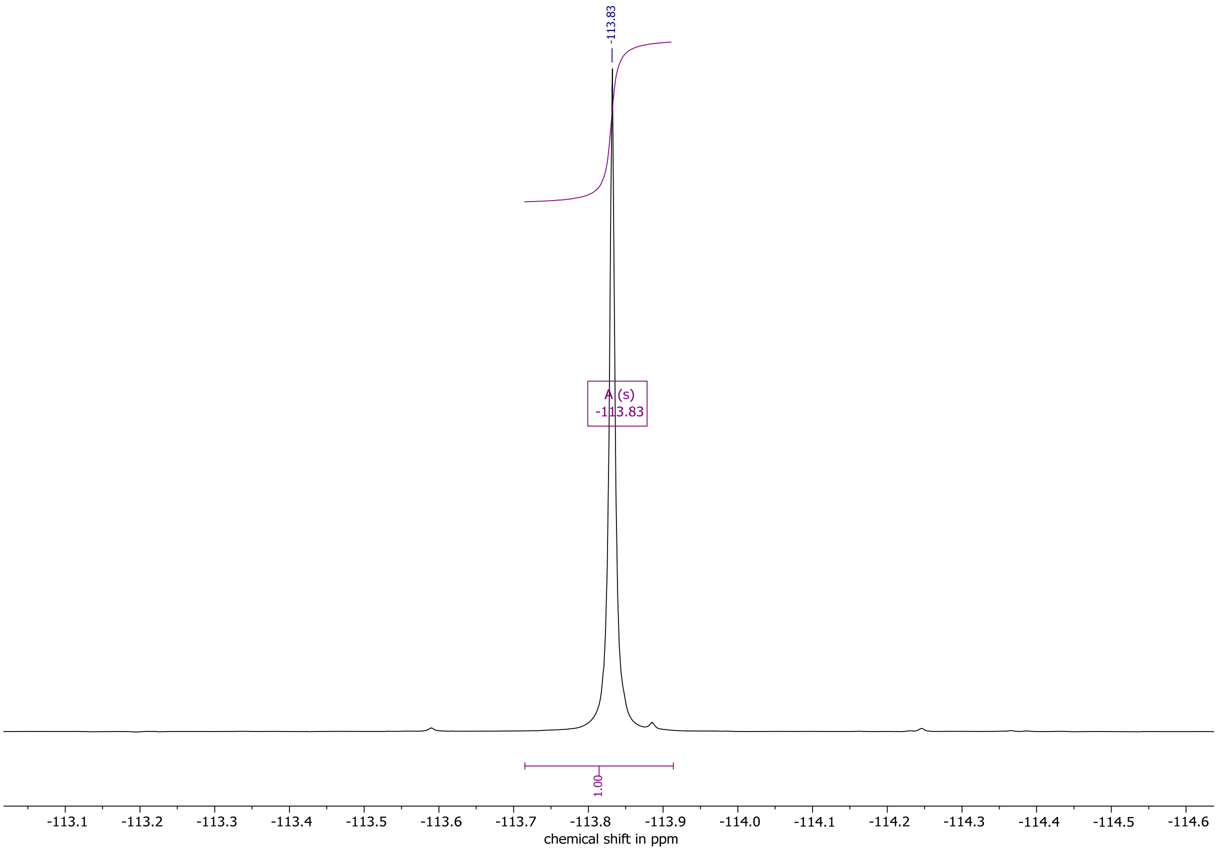

Figure S5. $^{19}F\{^{1}H\}$ NMR spectrum of compound $\mathbf{2}_{\boldsymbol{o}}$ in $CDCl_3$.

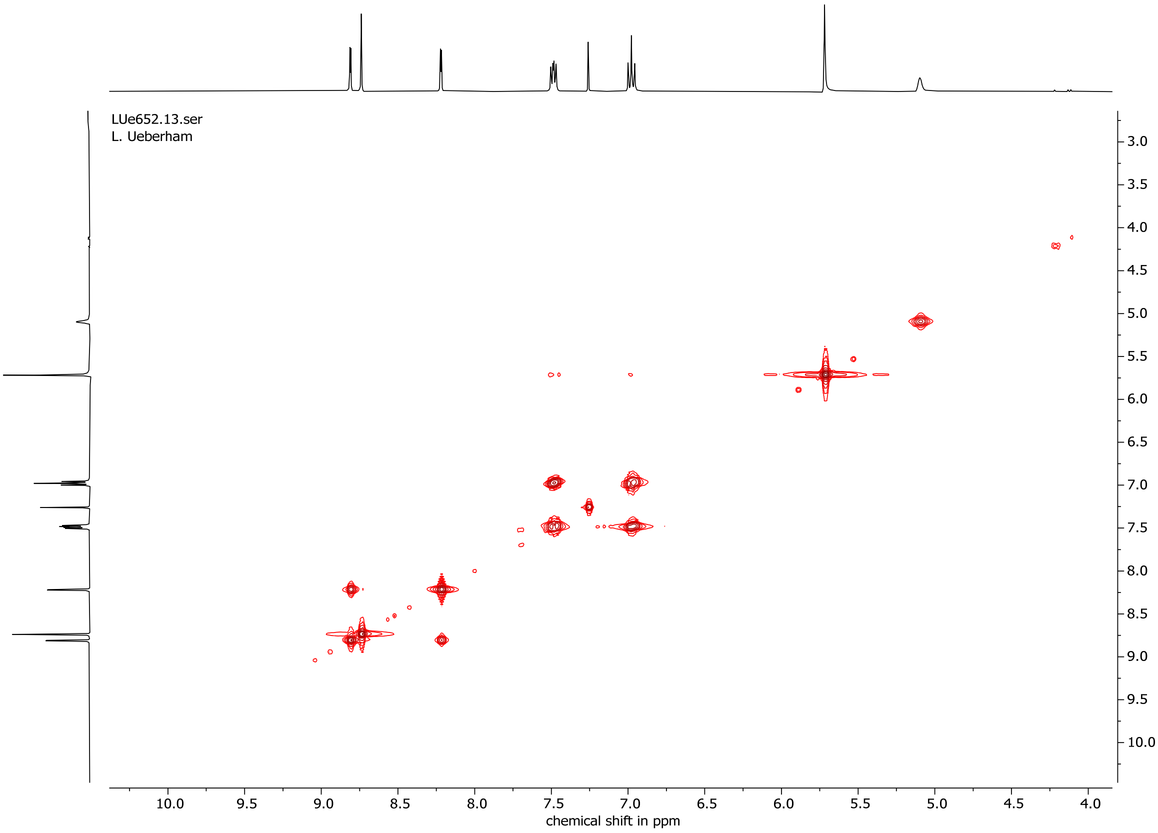

Figure S6. COSY (^1H, ^1H) NMR spectrum of compound **2o** in $CDCl_3$ (magnified spectrum, zoomed in).

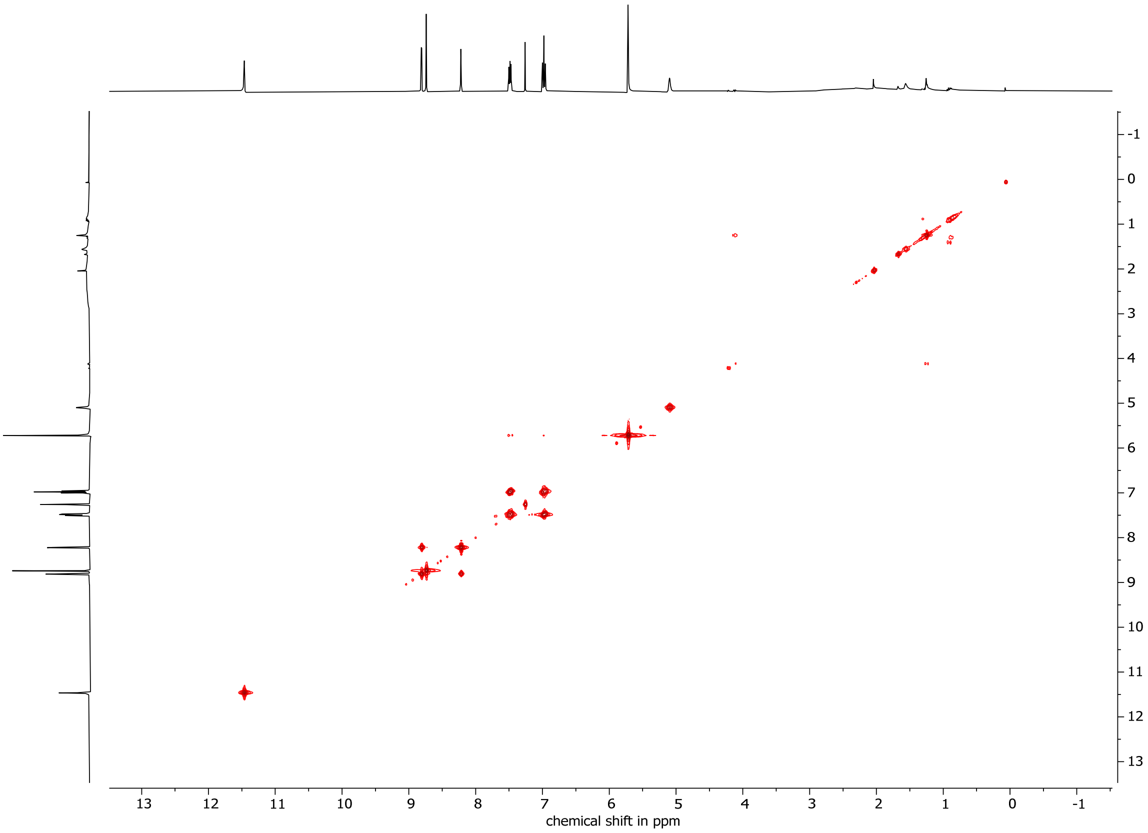

Figure S7. COSY (^1H, ^1H) NMR spectrum of compound **2o** in $CDCl_3$ full spectrum.

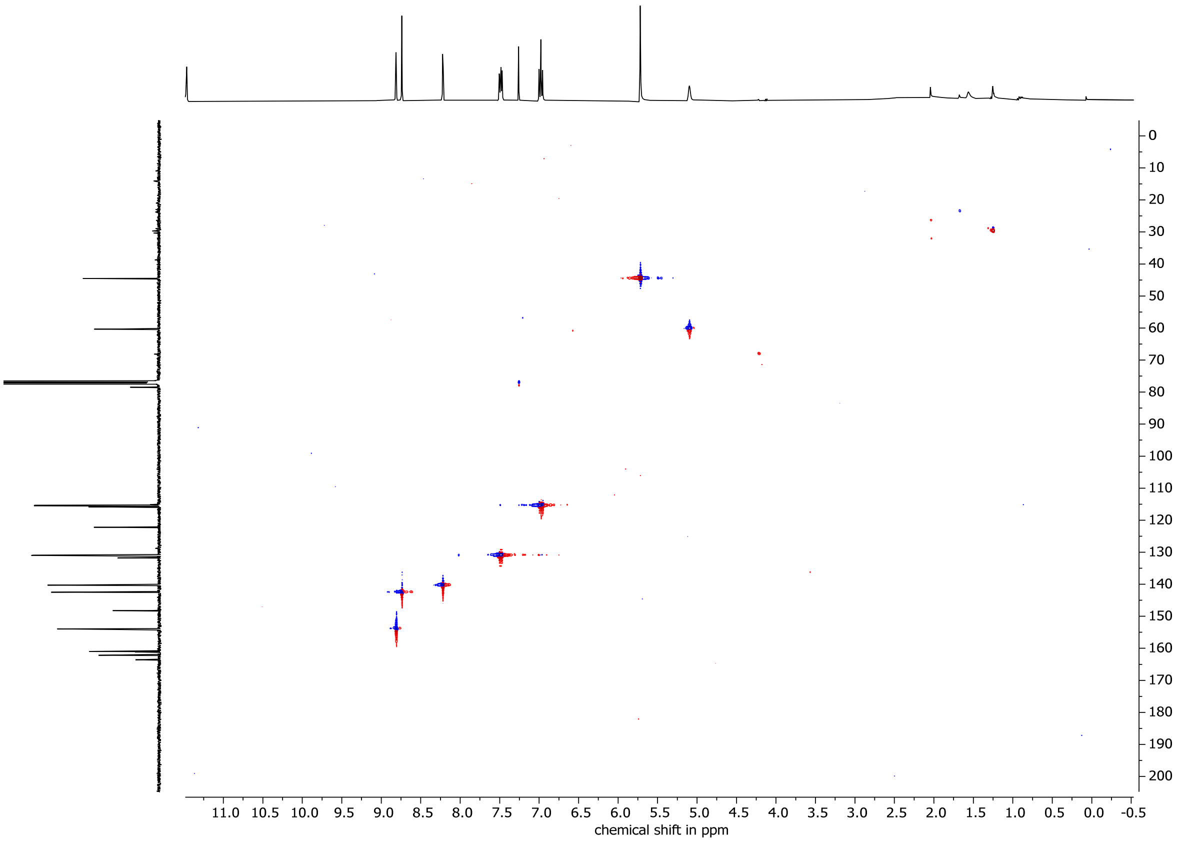

Figure S8. HSQC (^1H, ^{13}C) NMR spectrum of compound **2o** in $CDCl_3$.

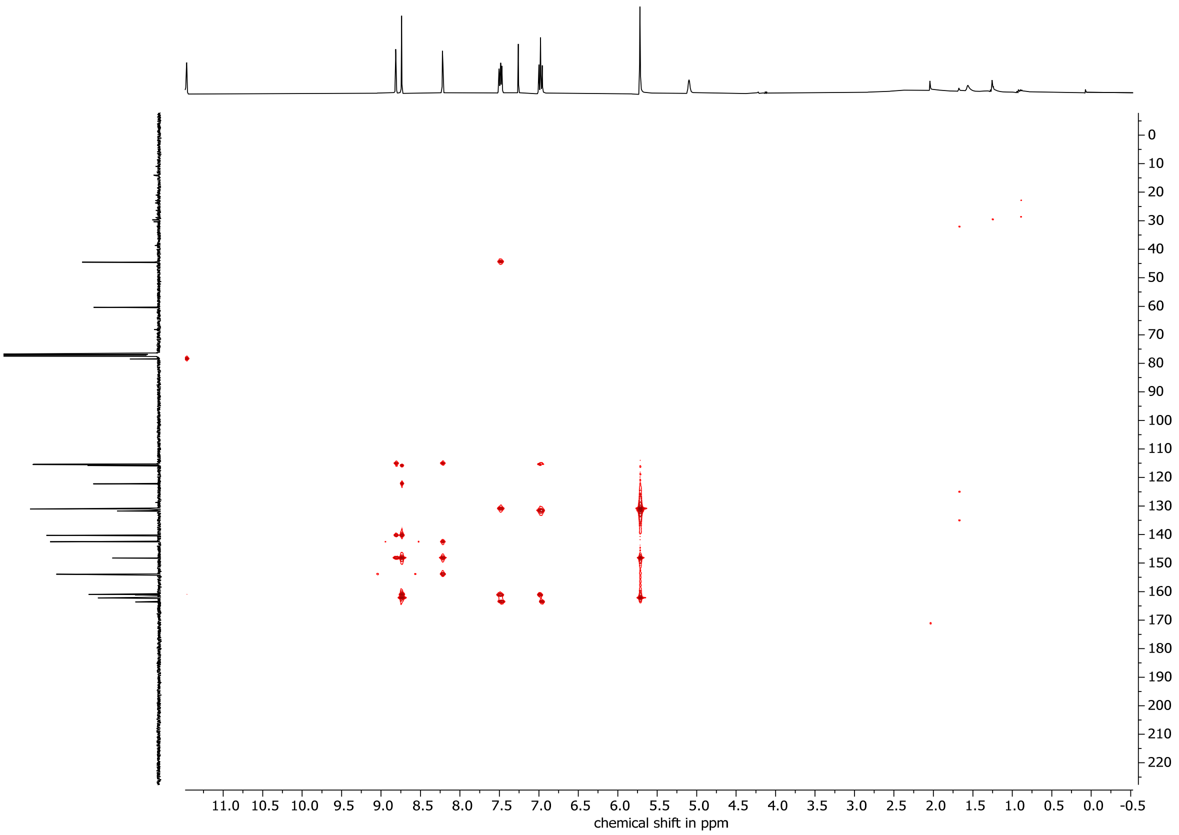

Figure S9. HMBC (^1H, ^{13}C) NMR spectrum of compound **2o** in $CDCl_3$.

Compound **2*m***

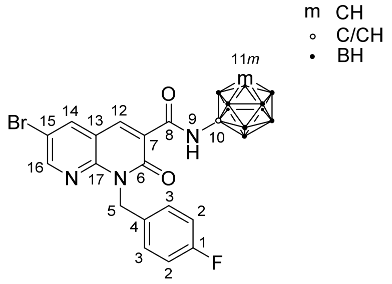

2*m*

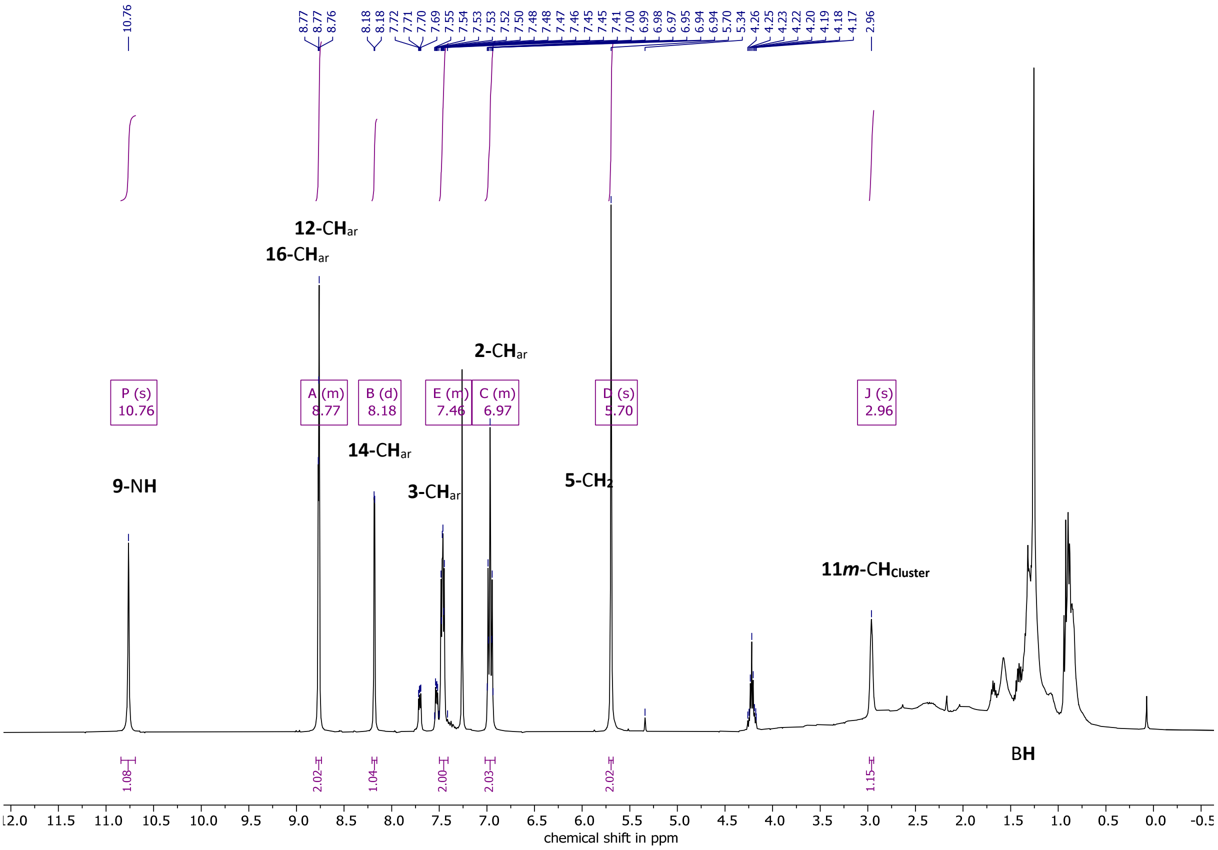

Figure S10. ^{1}H NMR spectrum of compound **2*m*** in $CDCl_3$.

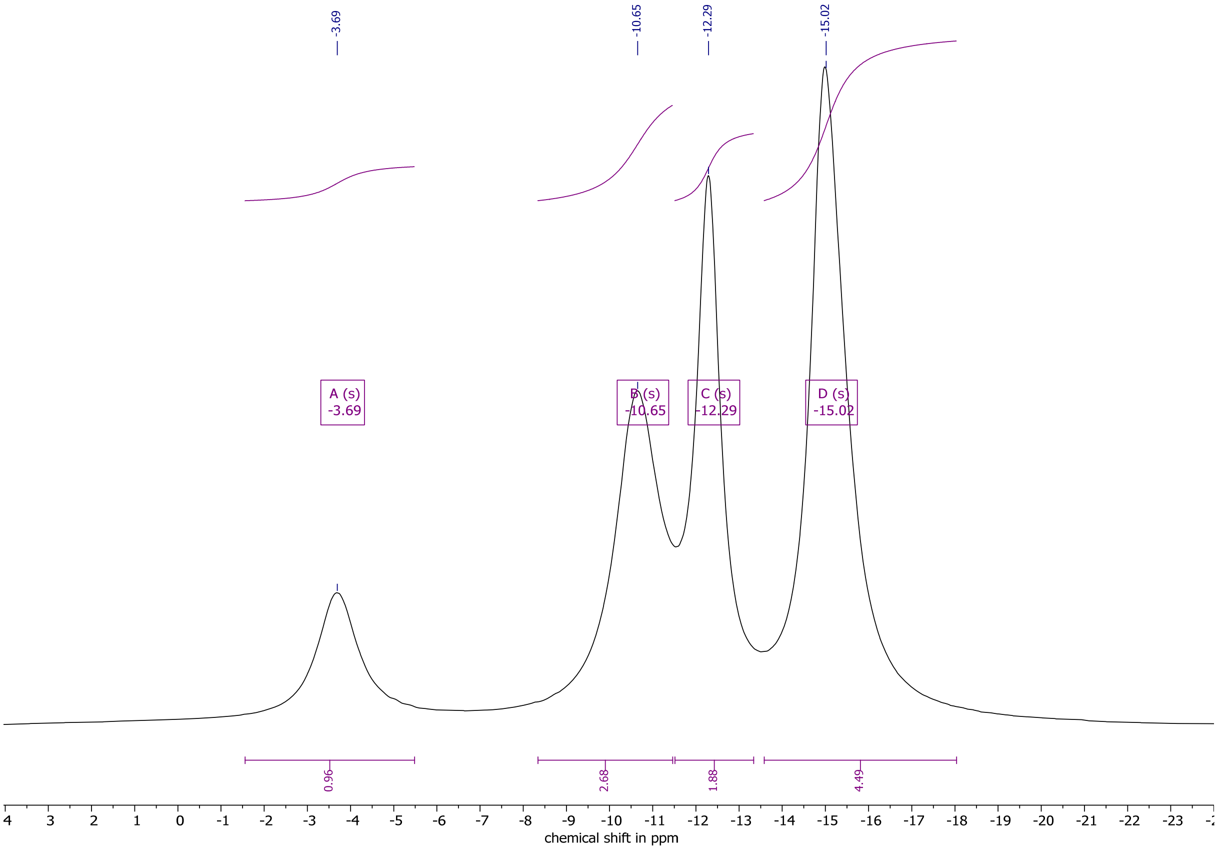

Figure S11. $^{11}B\{^1H\}$ NMR spectrum of compound $\mathbf{2}_{\boldsymbol{m}}$ in $CDCl_3$.

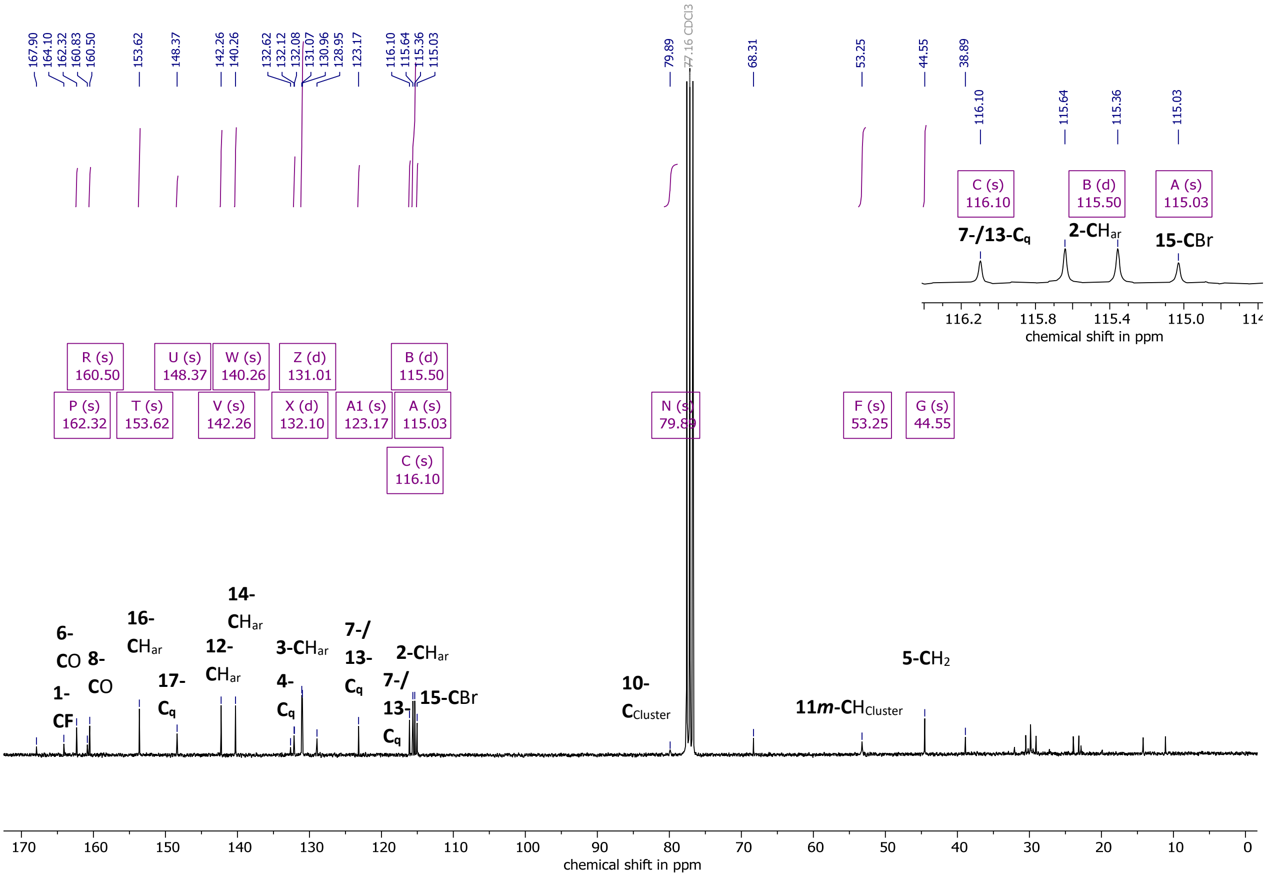

Figure S12. $^{13}C\{^1H\}$ NMR spectrum of compound $\mathbf{2}_{\boldsymbol{m}}$ in $CDCl_3$.

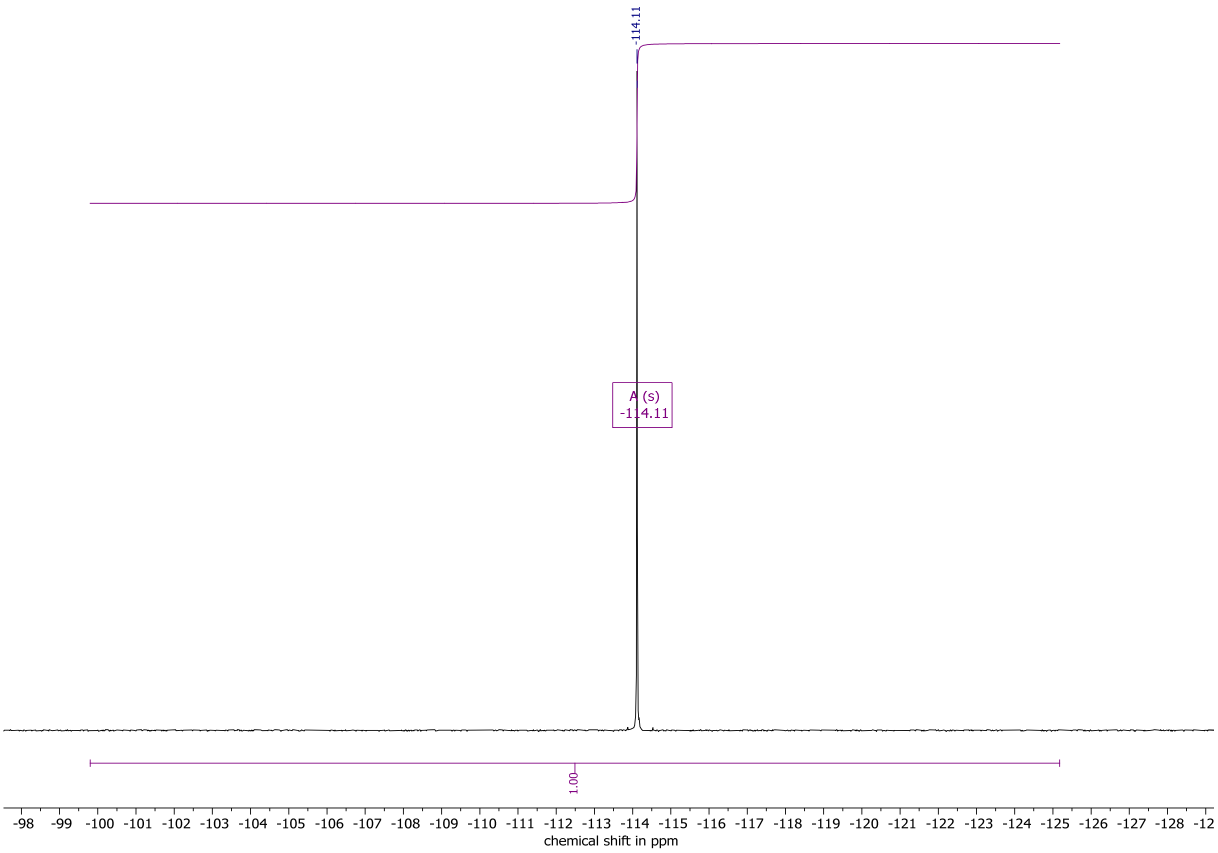

Figure S13. $^{19}F\{^{1}H\}$ NMR spectrum of compound **2*m*** in $CDCl_3$.

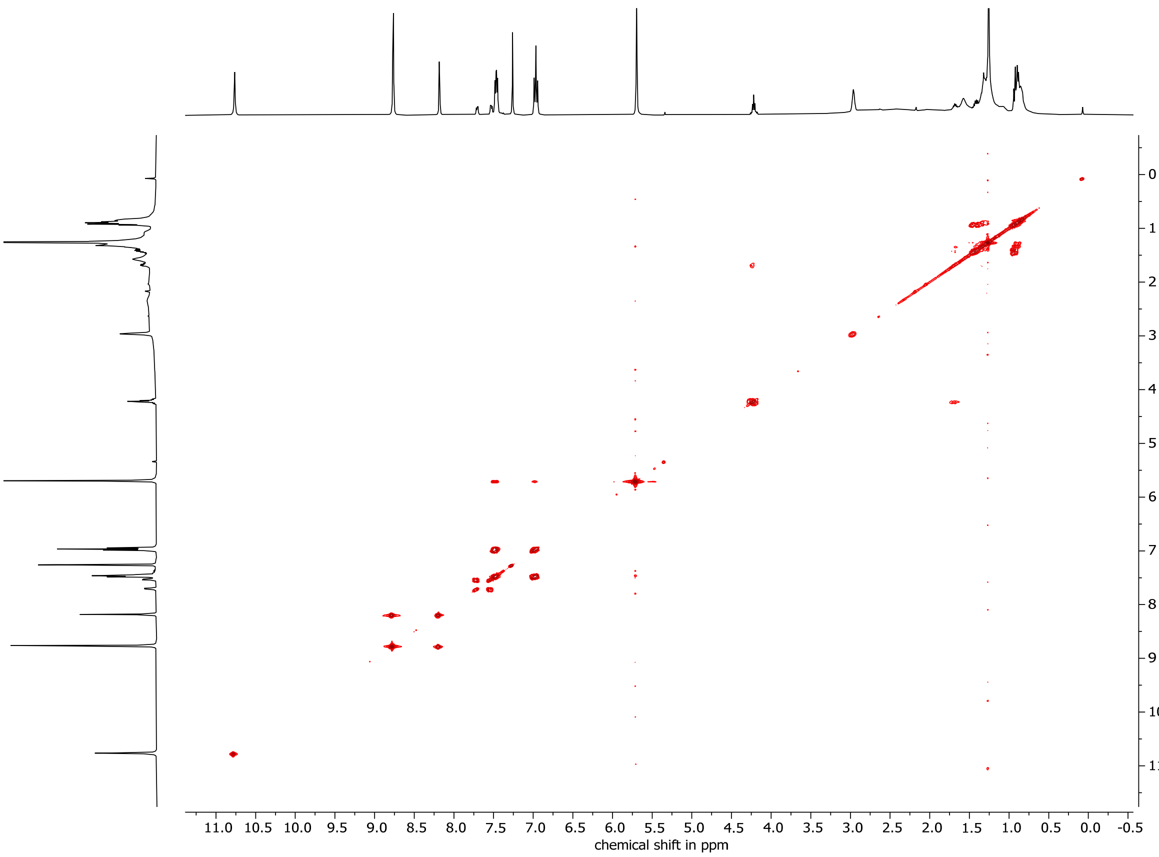

Figure S14. COSY (^{1}H, ^{1}H) NMR spectrum of compound **2*m*** in $CDCl_3$.

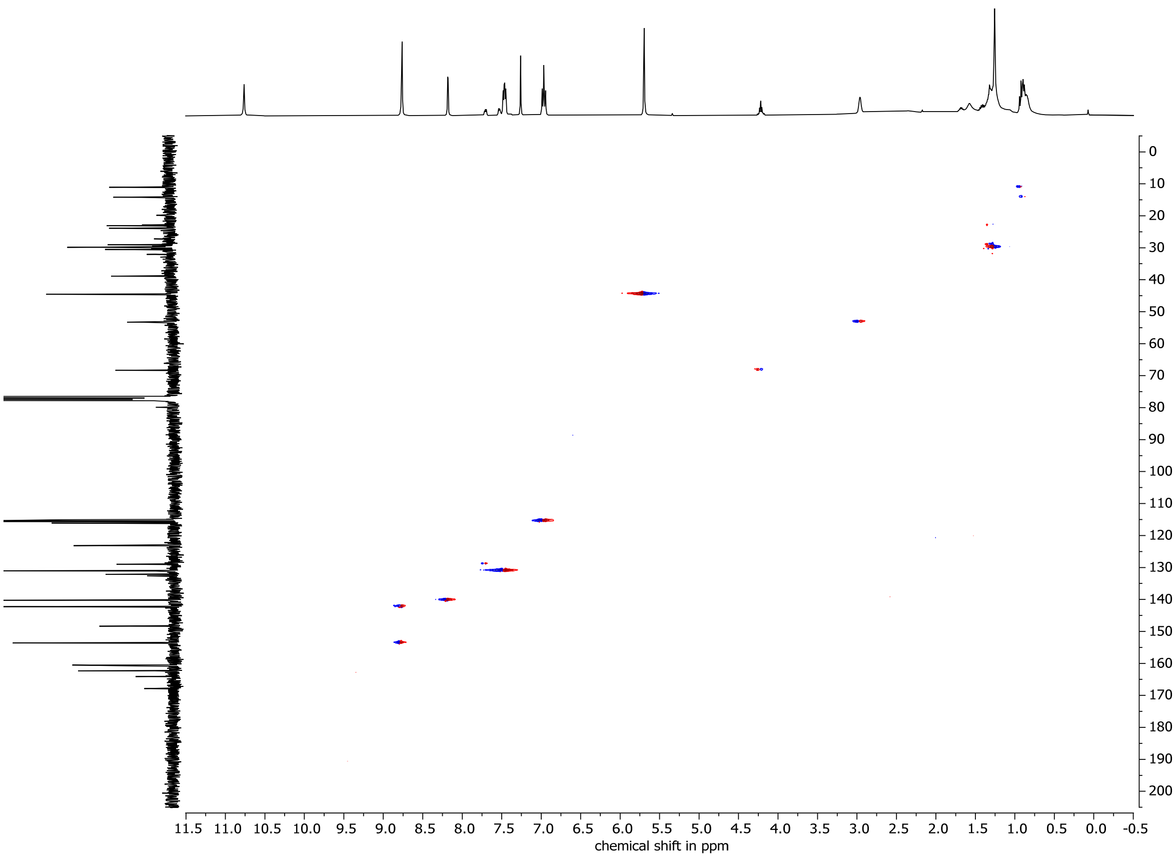

Figure S15. HSQC (^{1}H, ^{13}C) NMR spectrum of compound **2*m*** in $CDCl_3$.

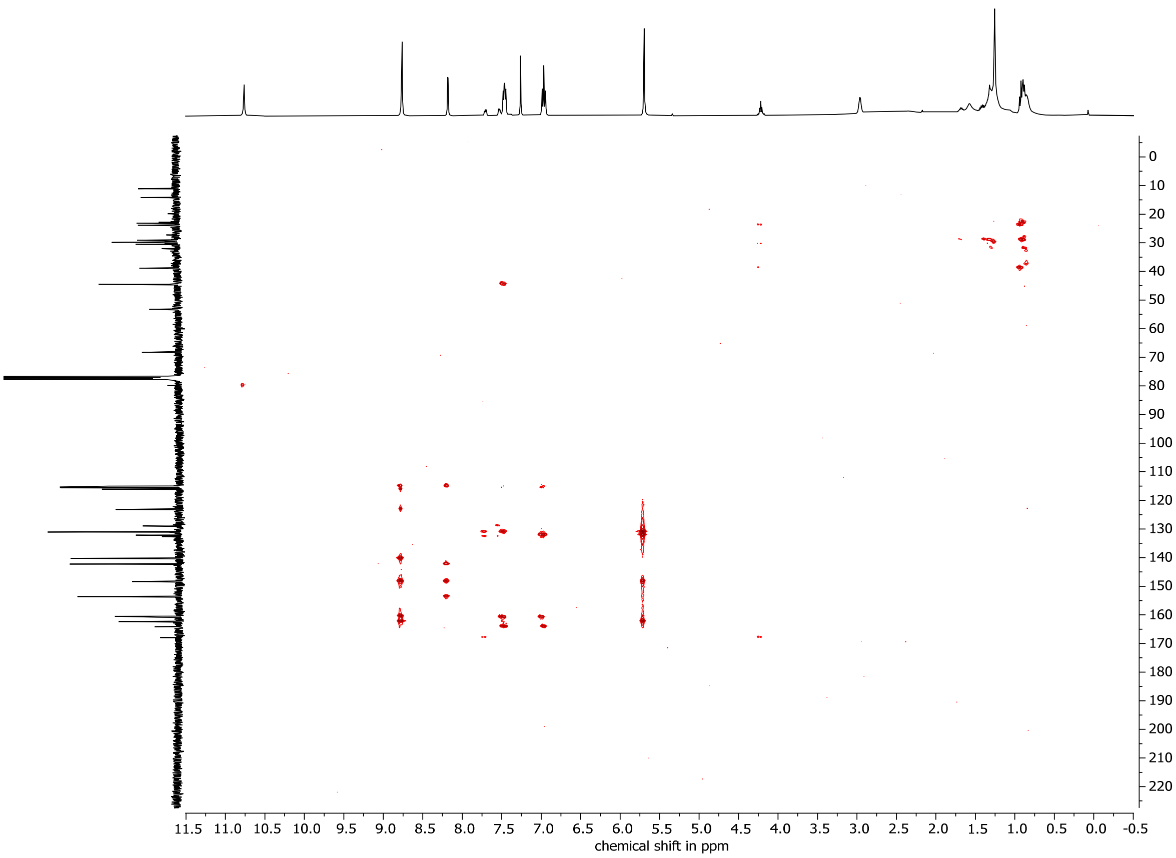

Figure S16. HMBC (^{1}H, ^{13}C) NMR spectrum of compound **2*m*** in $CDCl_3$.

Compound **2*p***

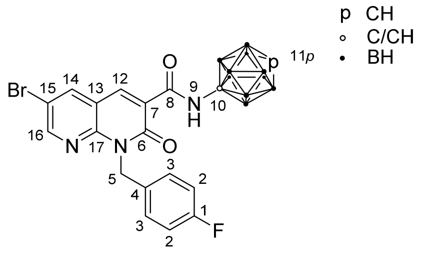

2*p*

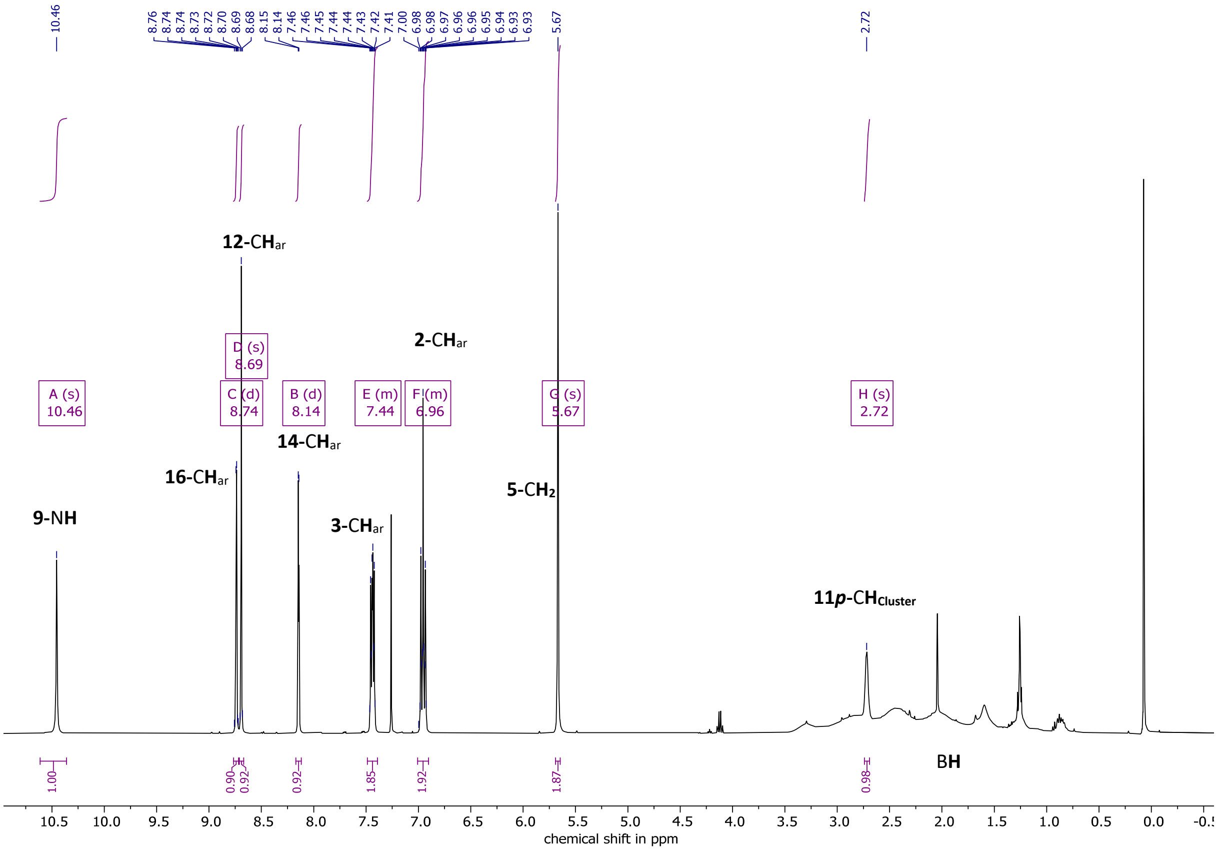

Figure S17. ^{1}H NMR spectrum of compound **2*p*** in $CDCl_3$.

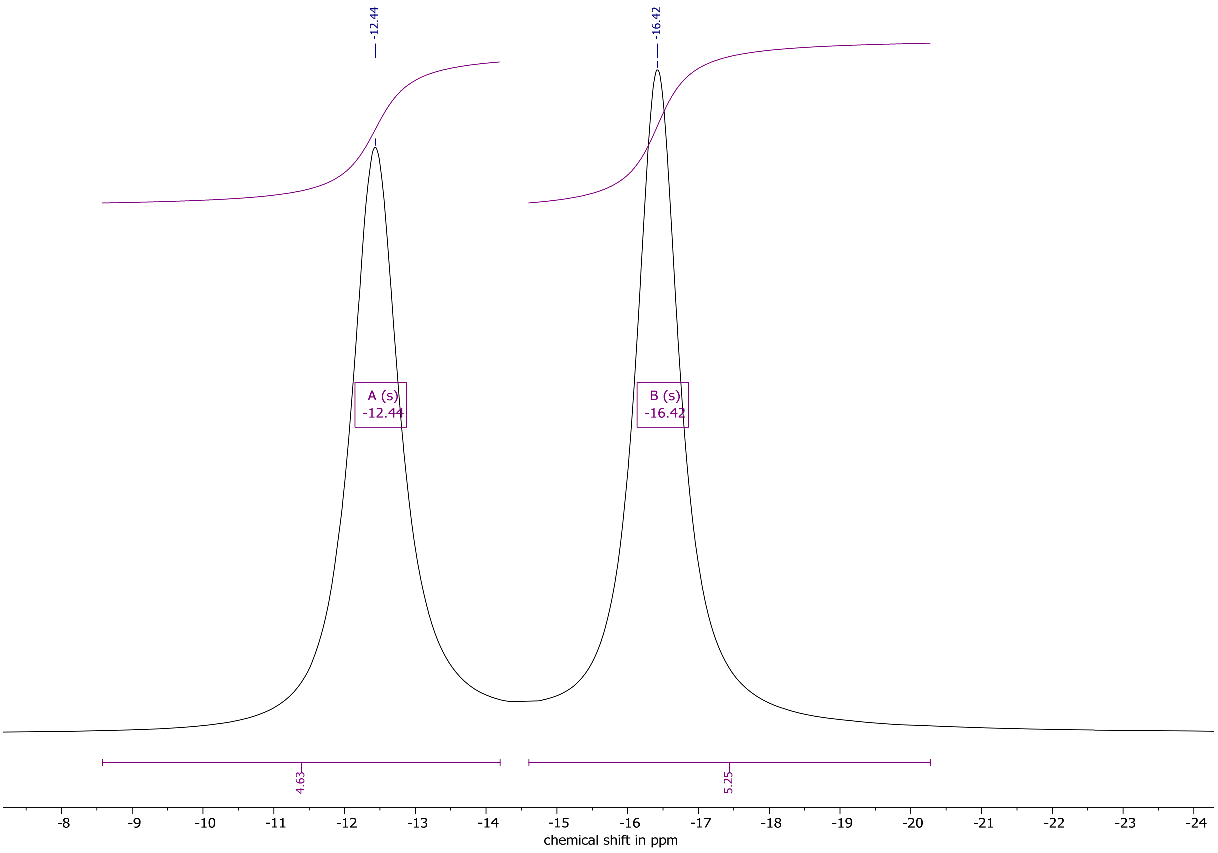

Figure S18. $^{11}B\{^1H\}$ NMR spectrum of compound $\mathbf{2}_{\boldsymbol{p}}$ in $CDCl_3$.

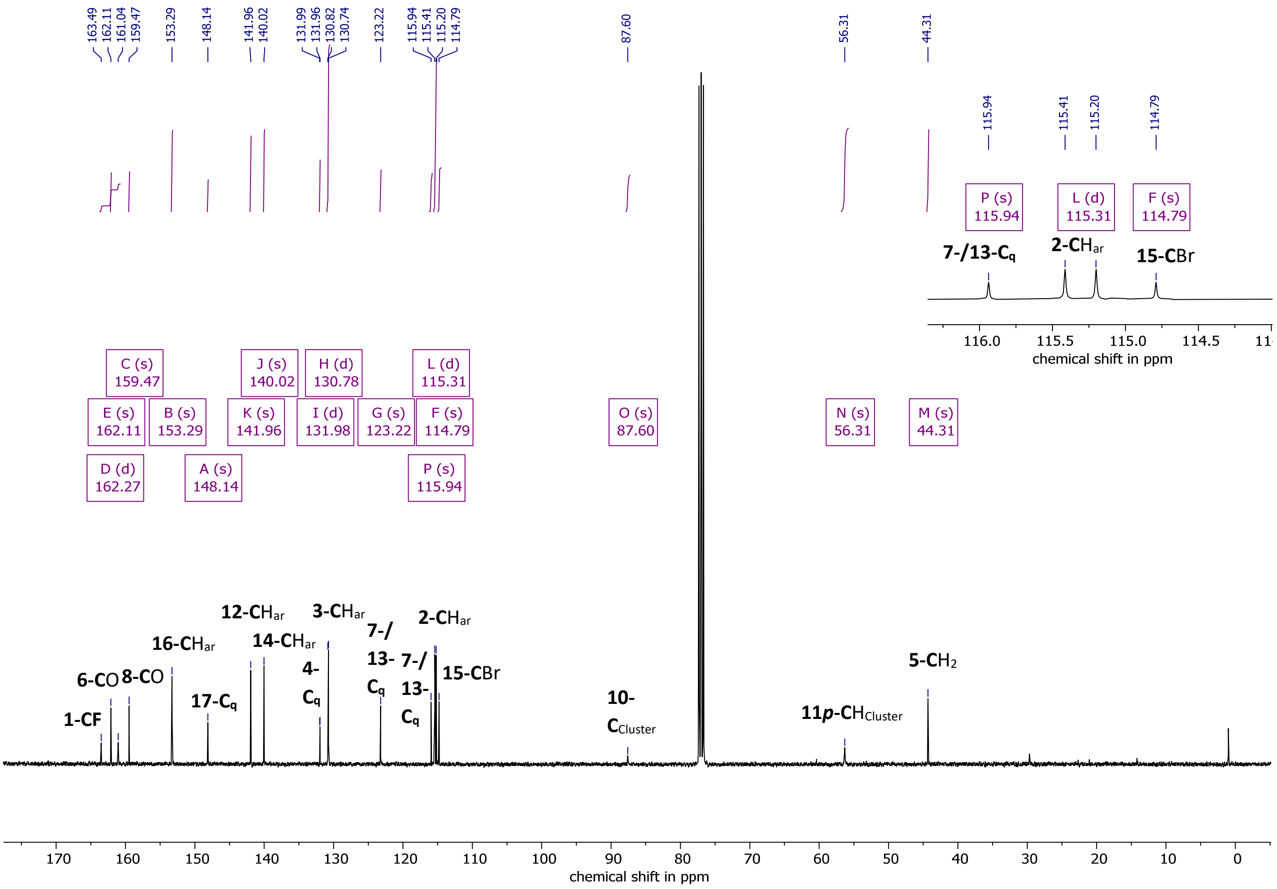

Figure S19. $^{13}C\{^1H\}$ NMR spectrum of compound $\mathbf{2}_{\boldsymbol{p}}$ in $CDCl_3$.

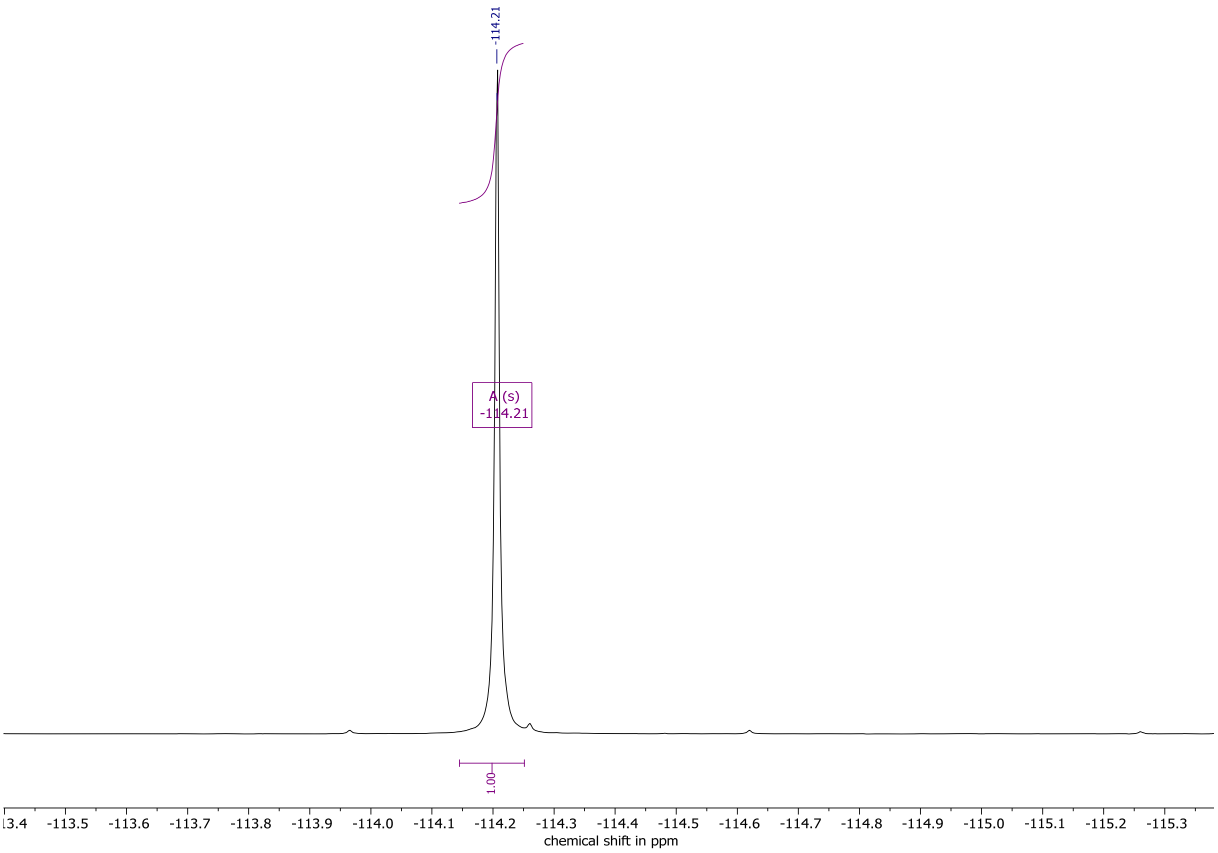

Figure S20. $^{19}F\{^{1}H\}$ NMR spectrum of compound **2*p*** in $CDCl_3$.

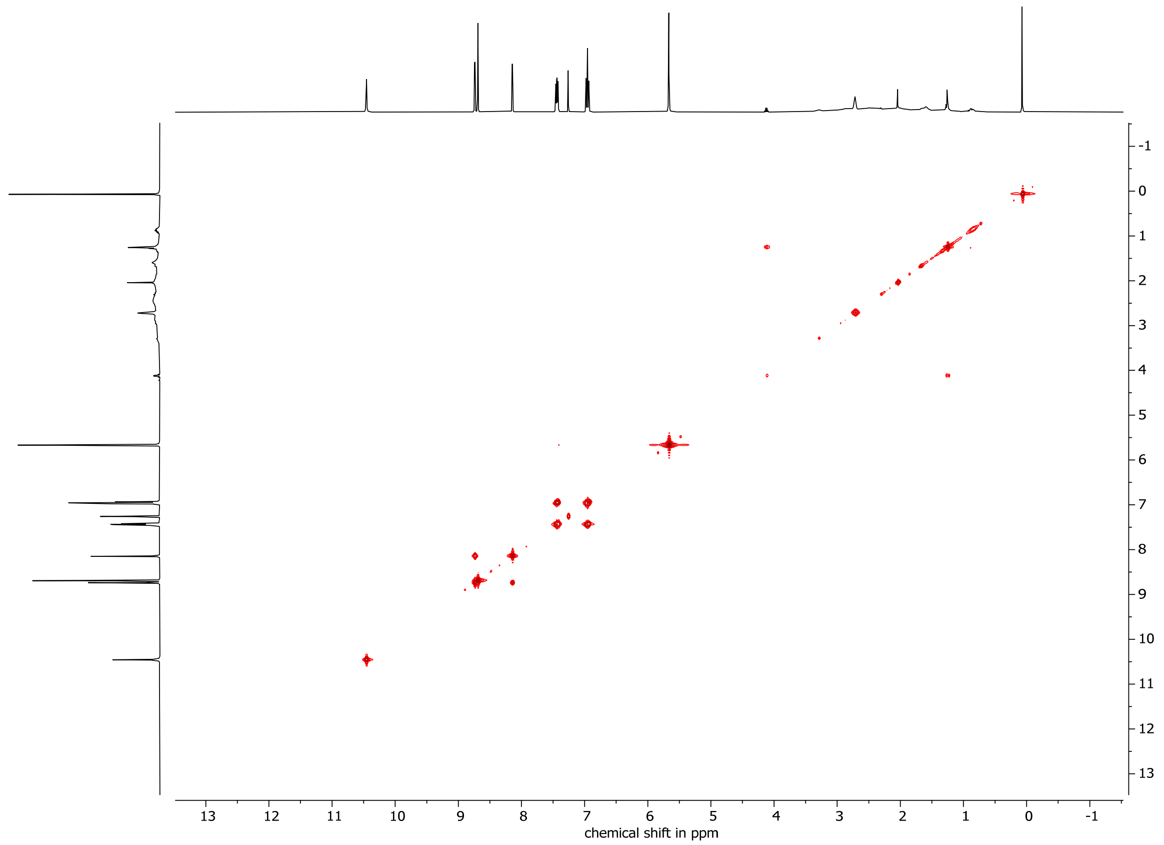

Figure S21. COSY (^{1}H, ^{1}H) NMR spectrum of compound **2*p*** in $CDCl_3$.

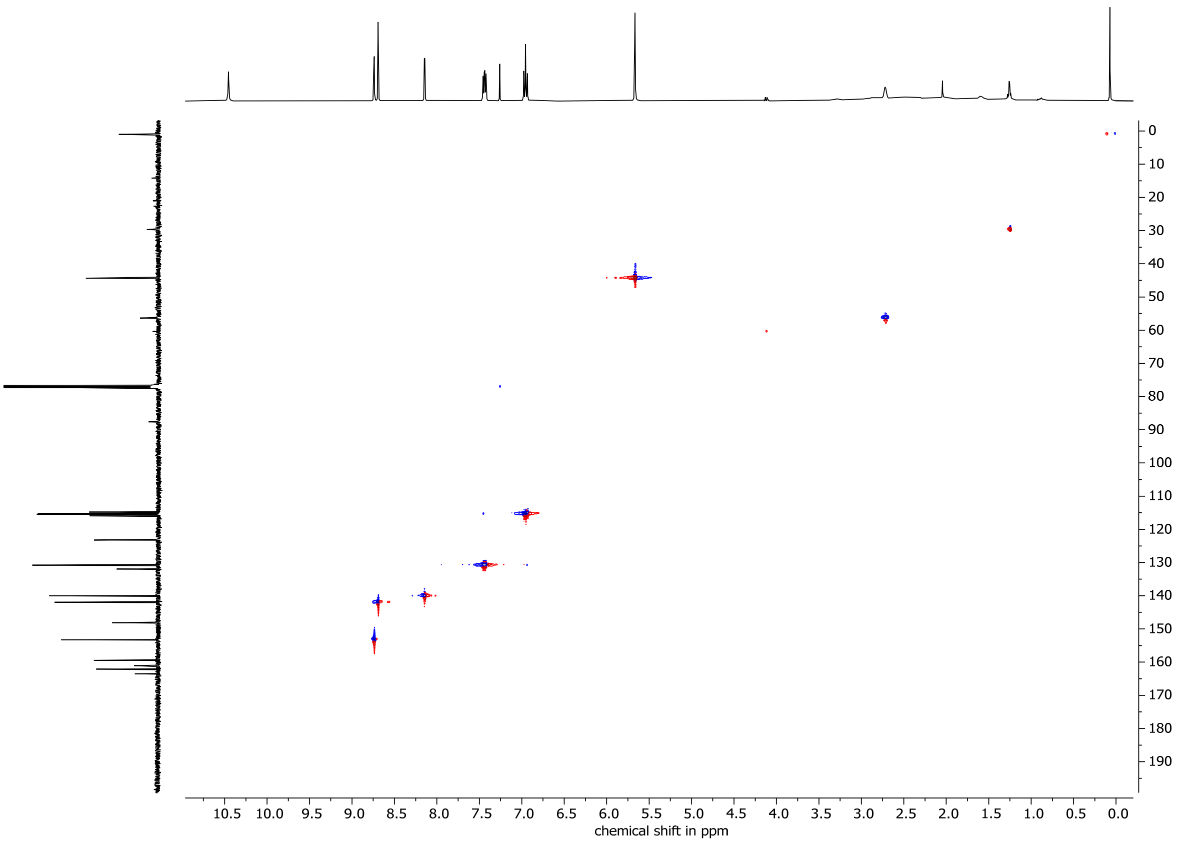

Figure S22. HSQC (^{1}H, ^{13}C) NMR spectrum of compound **2*p*** in $CDCl_3$.

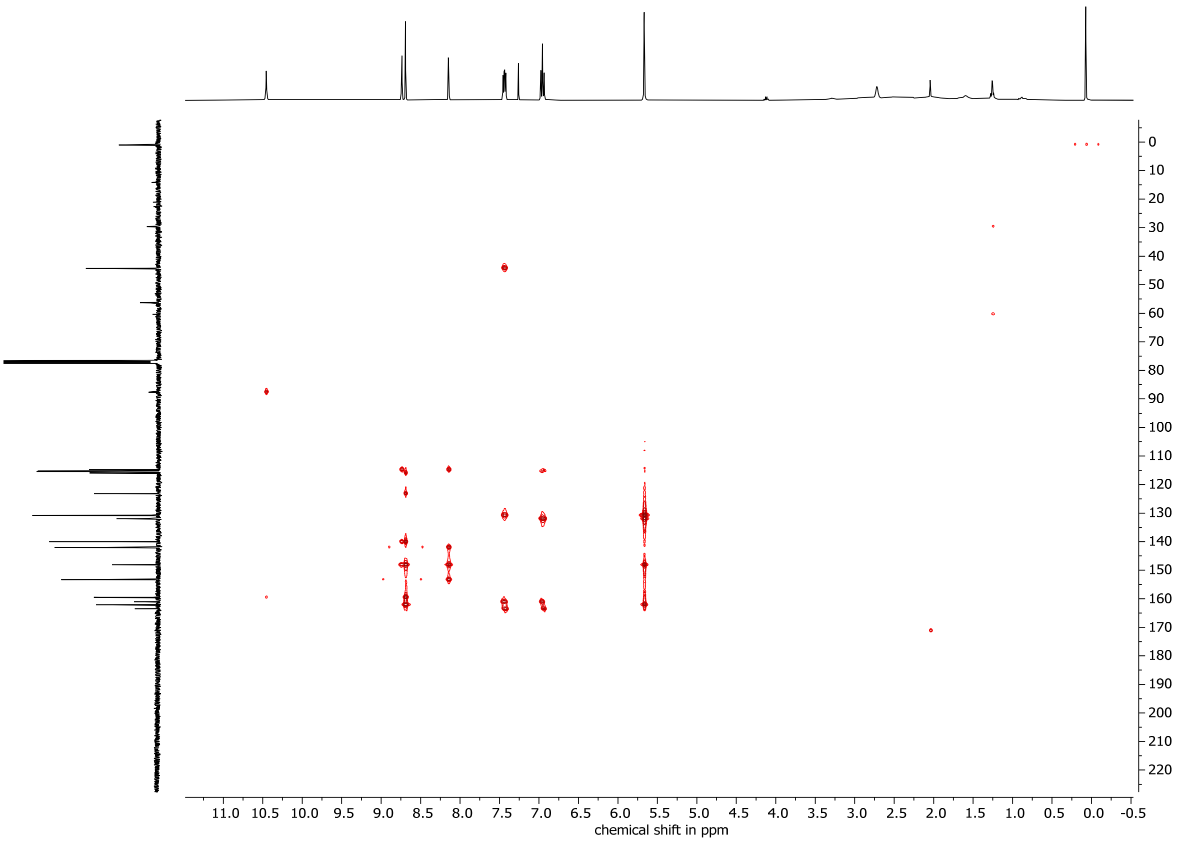

Figure S23. HMBC (^{1}H, ^{13}C) NMR spectrum of compound **2*p*** in $CDCl_3$.

Compound $\mathbf{3}_o$

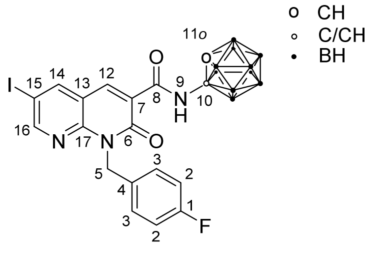

$\mathbf{3}_o$

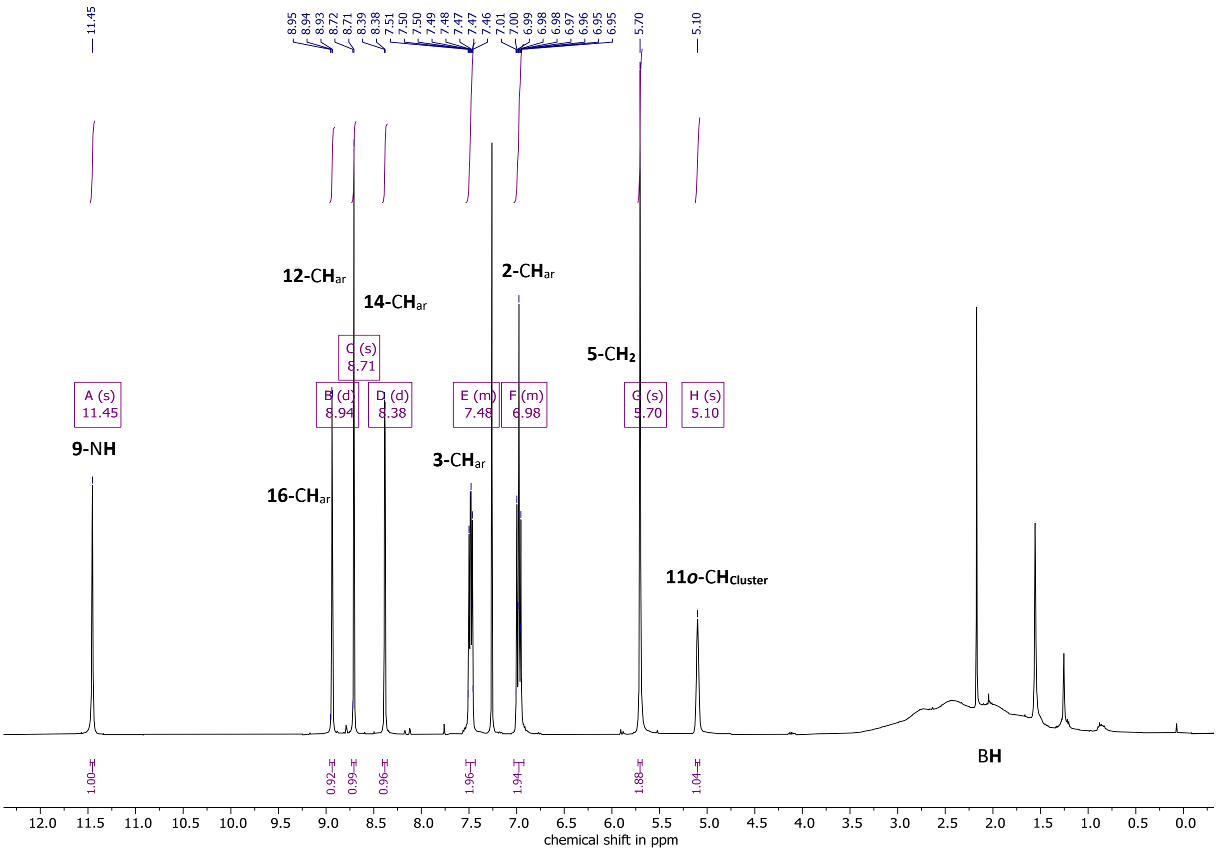

Figure S24. ^{1}H NMR spectrum of compound $\mathbf{3}_o$ in $CDCl_3$.

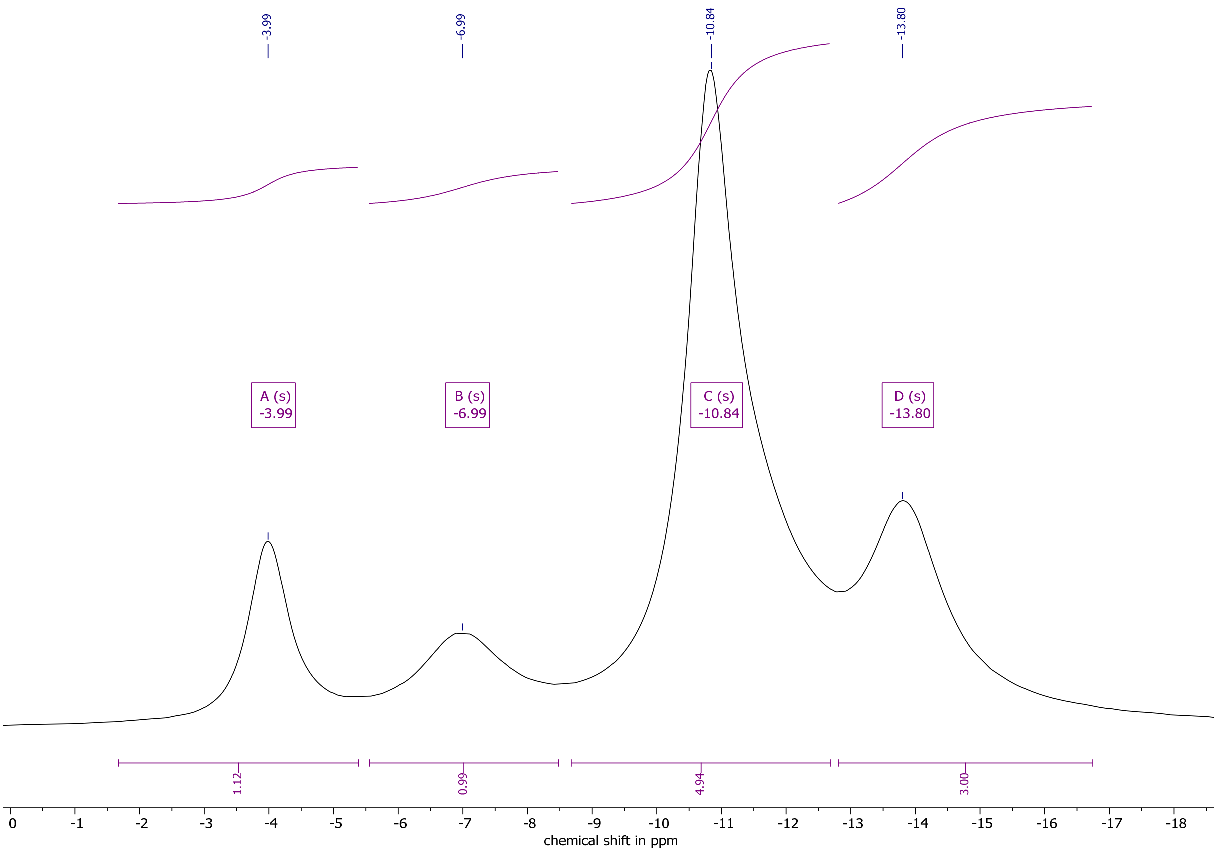

Figure S25. $^{11}B\{^{1}H\}$ NMR spectrum of compound **3o** in $CDCl_3$.

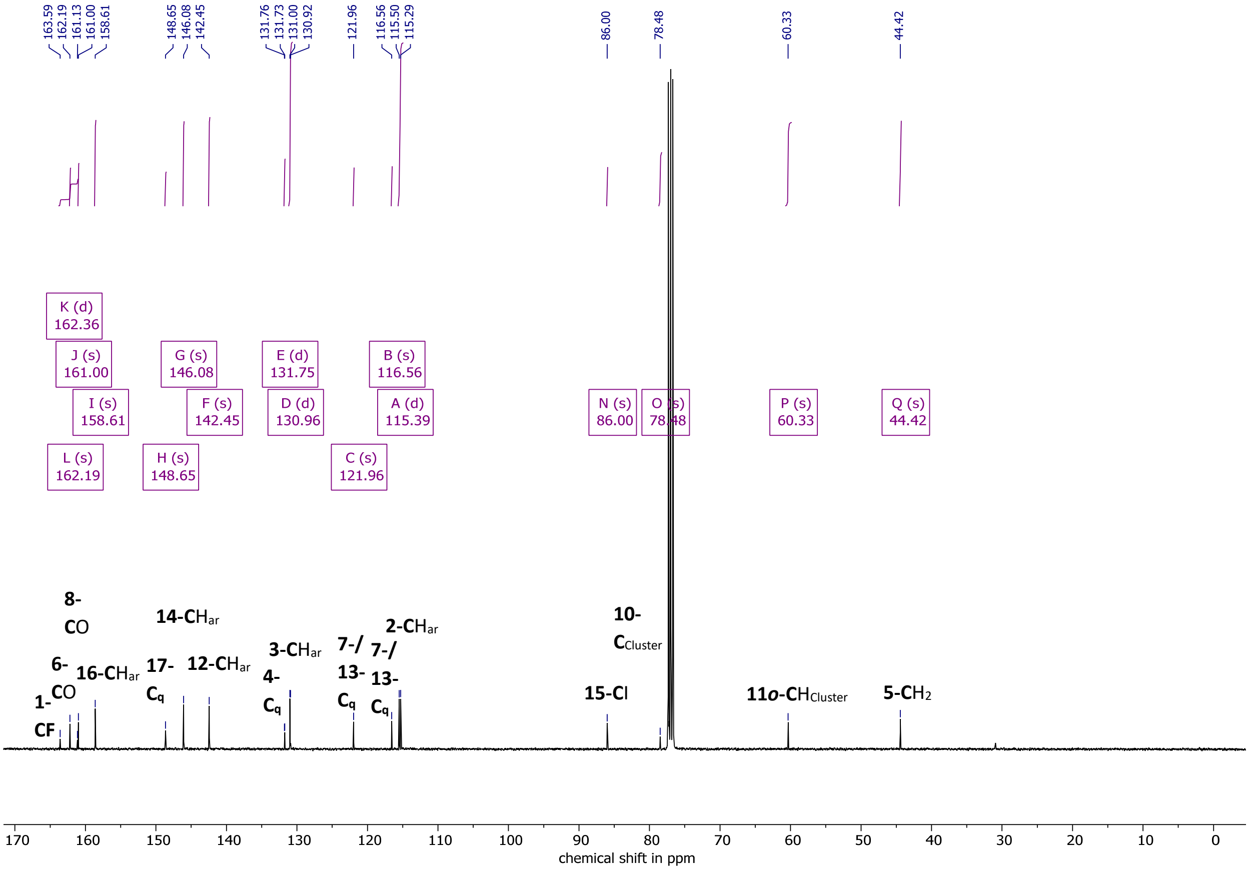

Figure S26. $^{13}C\{^{1}H\}$ NMR spectrum of compound **3o** in $CDCl_3$.

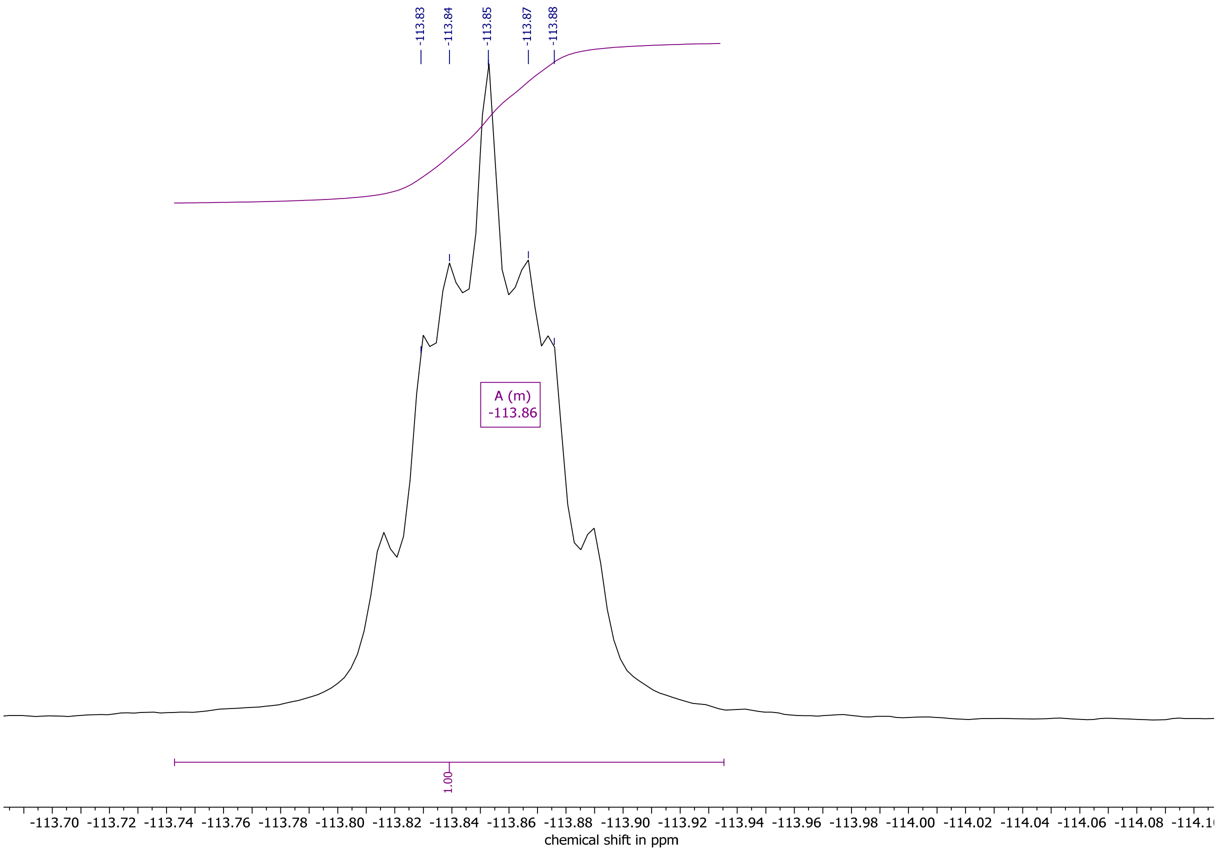

Figure S27. $^{19}F\{^{1}H\}$ NMR spectrum of compound **3o** in $CDCl_3$.

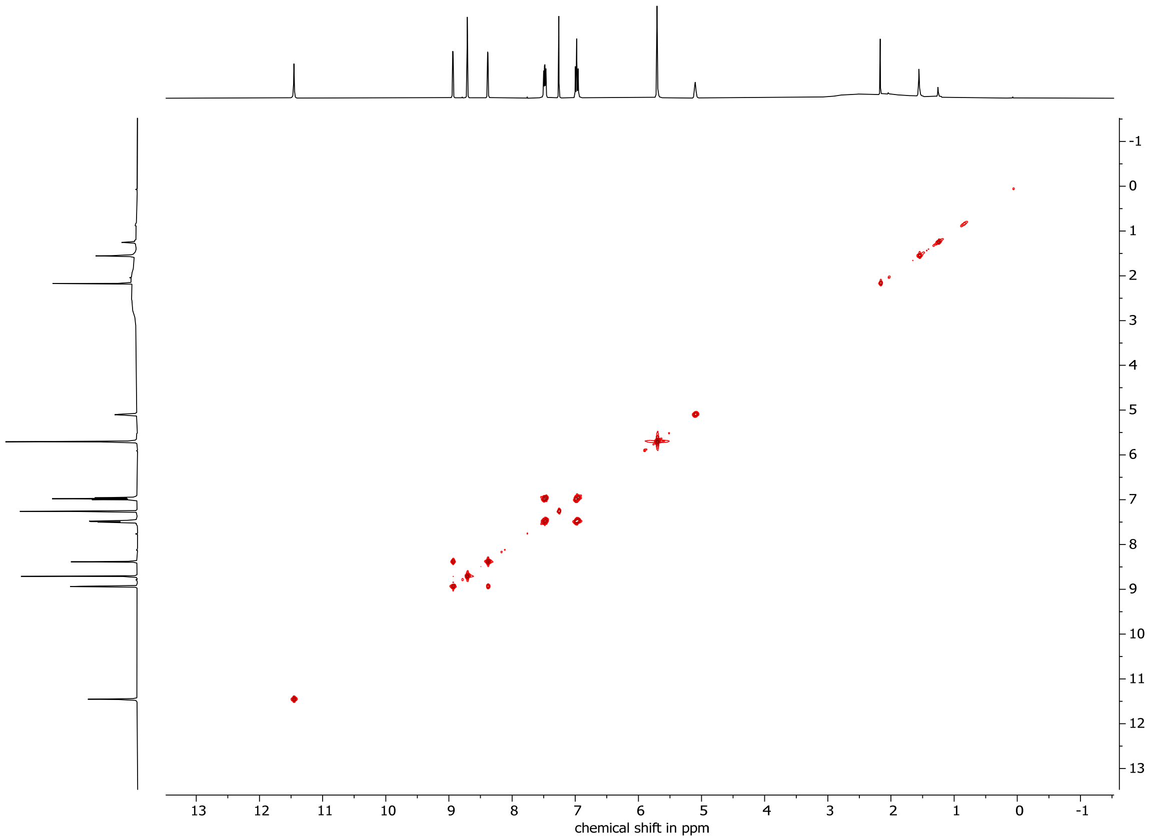

Figure S28. COSY (^{1}H, ^{1}H) NMR spectrum of compound **3o** in $CDCl_3$.

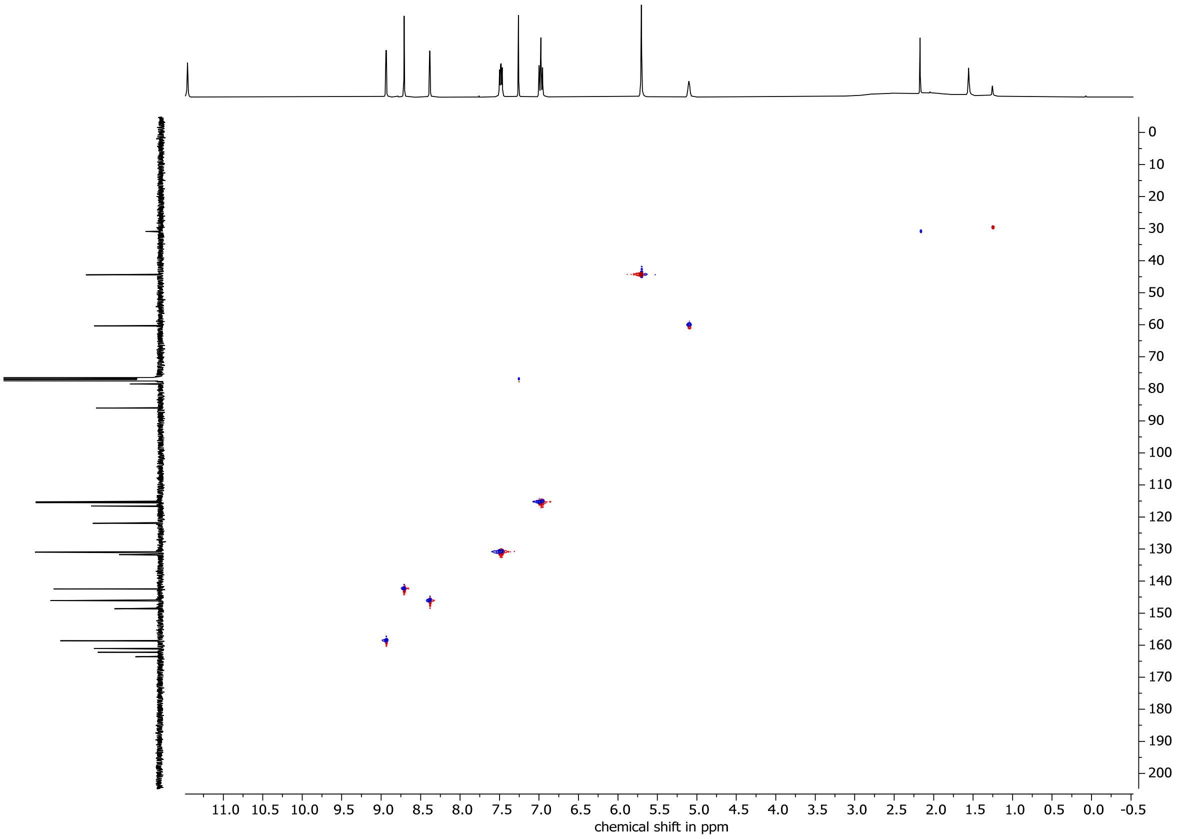

Figure S29. HSQC (^{1}H, ^{13}C) NMR spectrum of compound **3o** in $CDCl_3$.

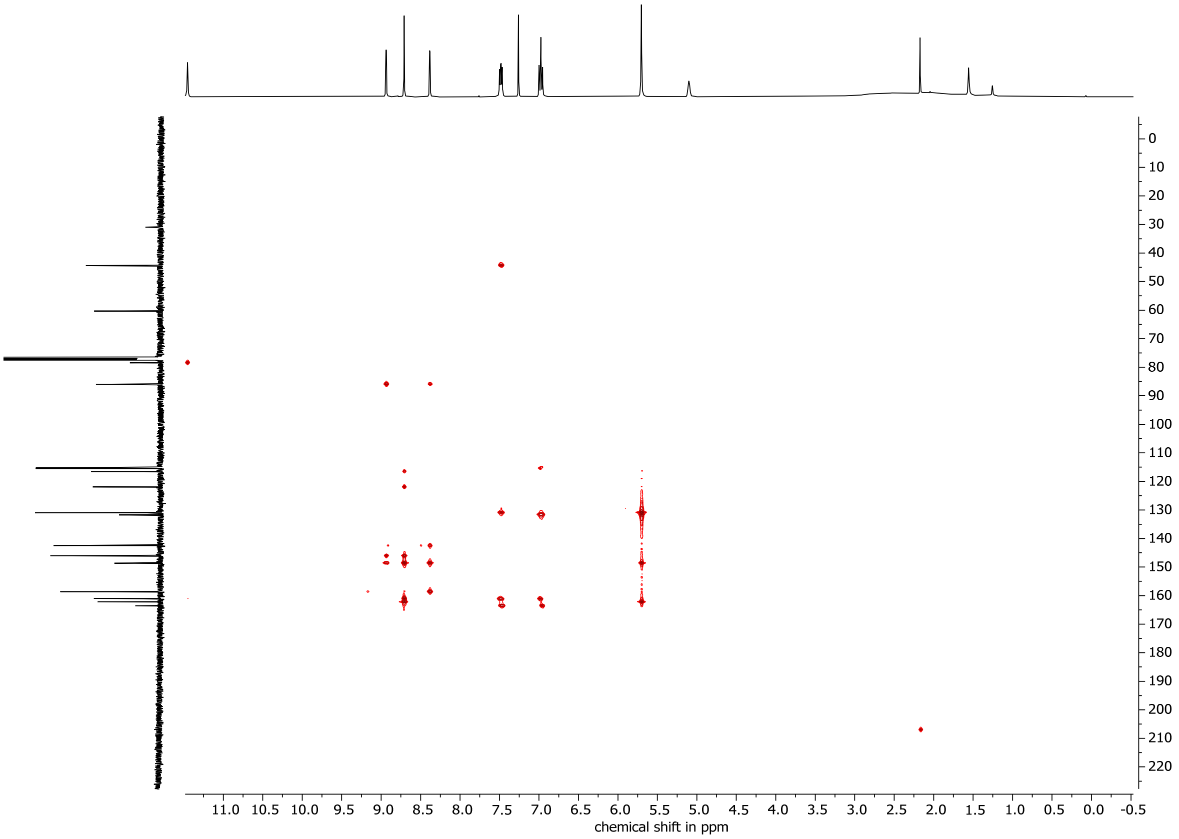

Figure S30. HMBC (^{1}H, ^{13}C) NMR spectrum of compound **3o** in $CDCl_3$.

Compound **3*m***

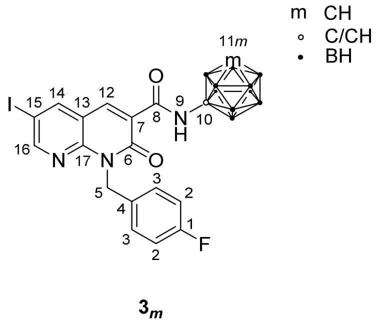

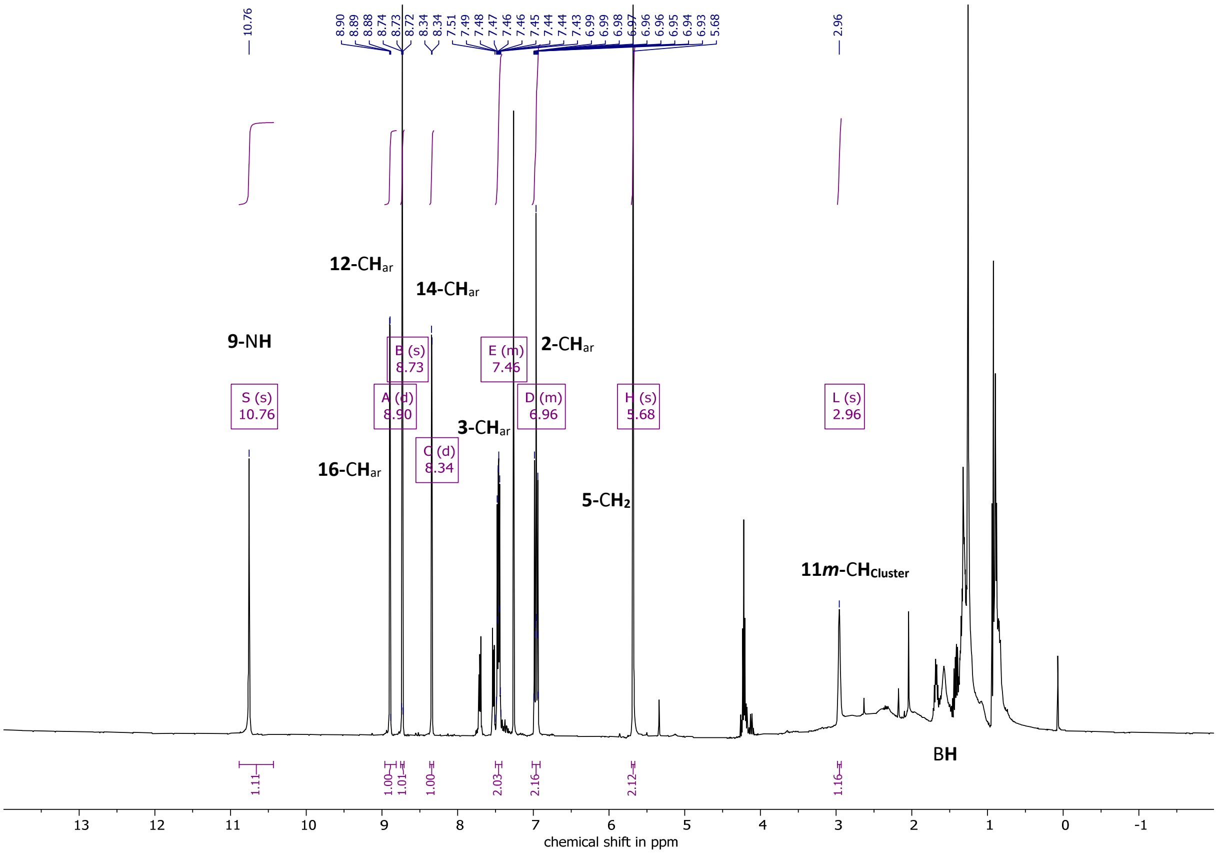

Figure S31. ^{1}H NMR spectrum of compound **3*m*** in $CDCl_3$.

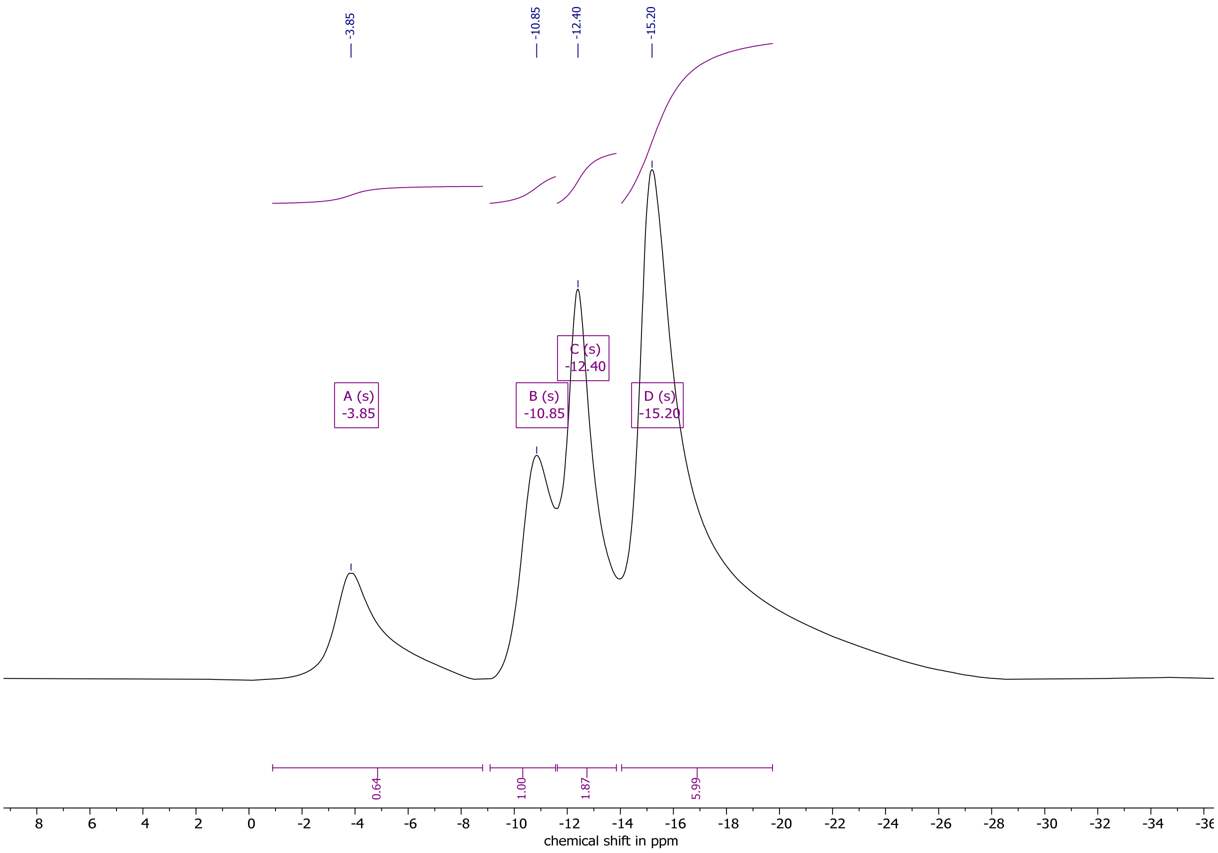

Figure S32. $^{11}B\{^{1}H\}$ NMR spectrum of compound **3*m*** in $CDCl_3$.

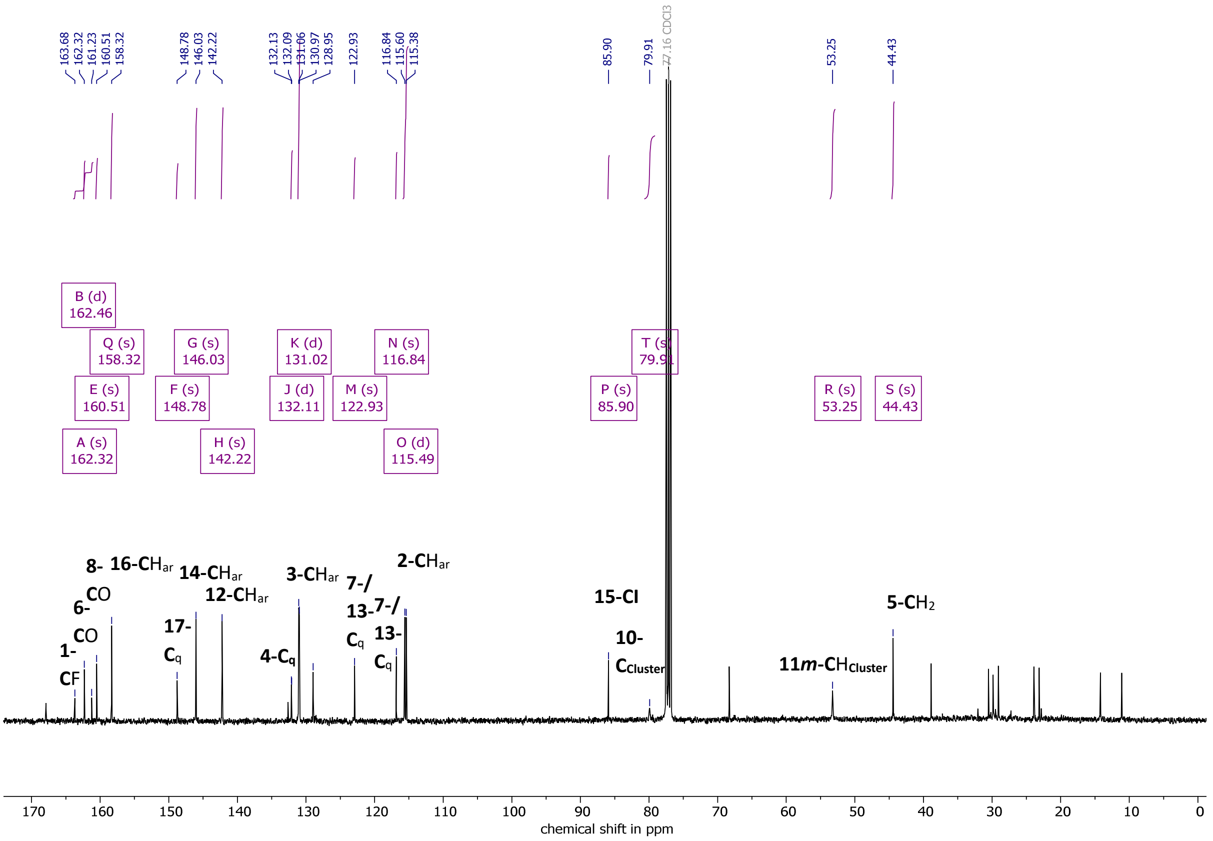

Figure S33. $^{13}C\{^{1}H\}$ NMR spectrum of compound **3*m*** in $CDCl_3$.

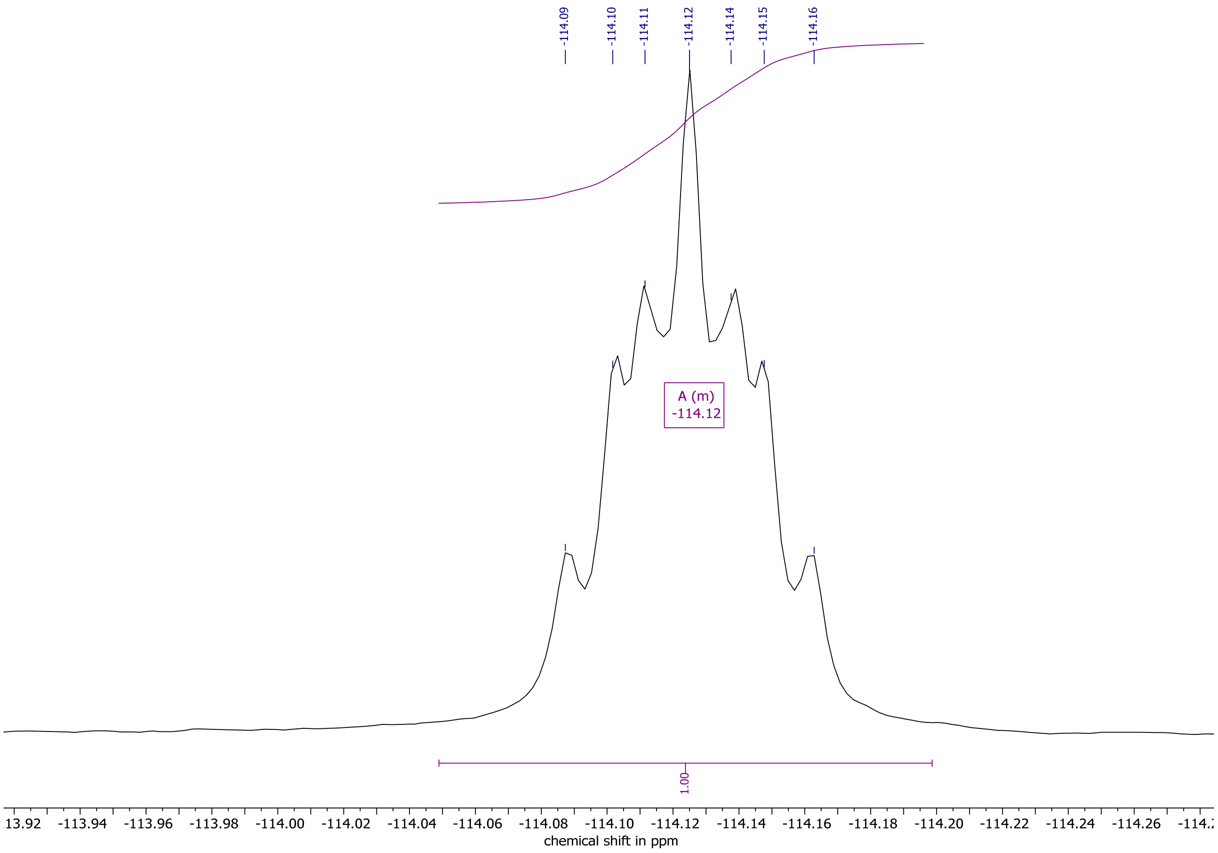

Figure S34. $^{19}F\{^{1}H\}$ NMR spectrum of compound **3*m*** in $CDCl_3$.

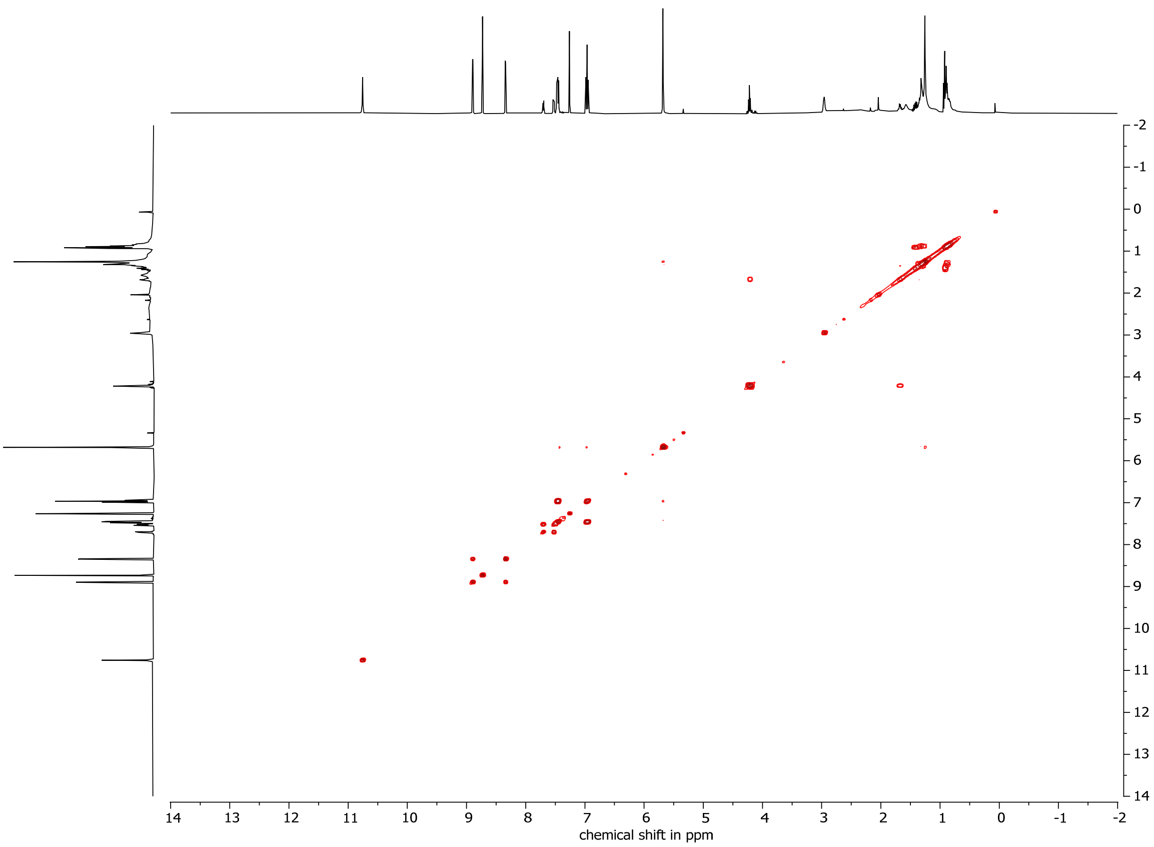

Figure S35. COSY (^{1}H, ^{1}H) NMR spectrum of compound **3*m*** in $CDCl_3$.

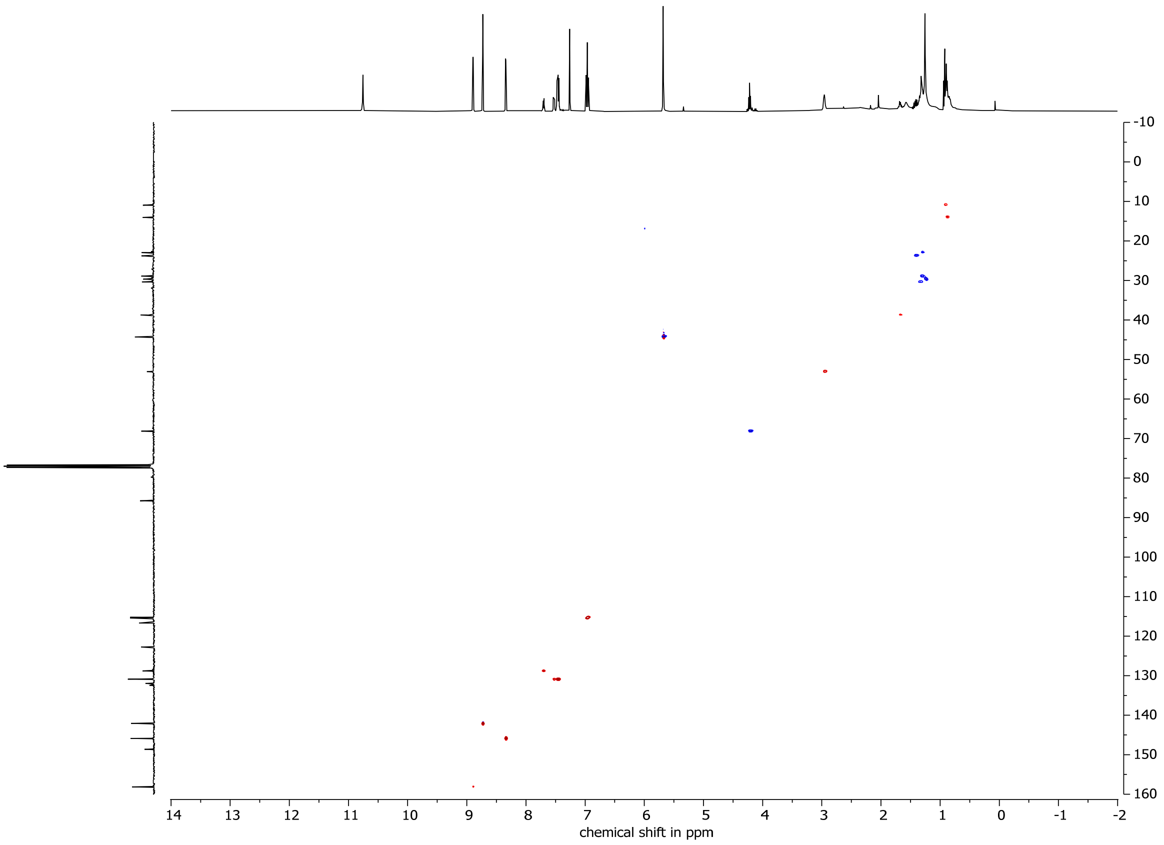

Figure S36. HSQC (^{1}H, ^{13}C) NMR spectrum of compound **3*m*** in $CDCl_3$.

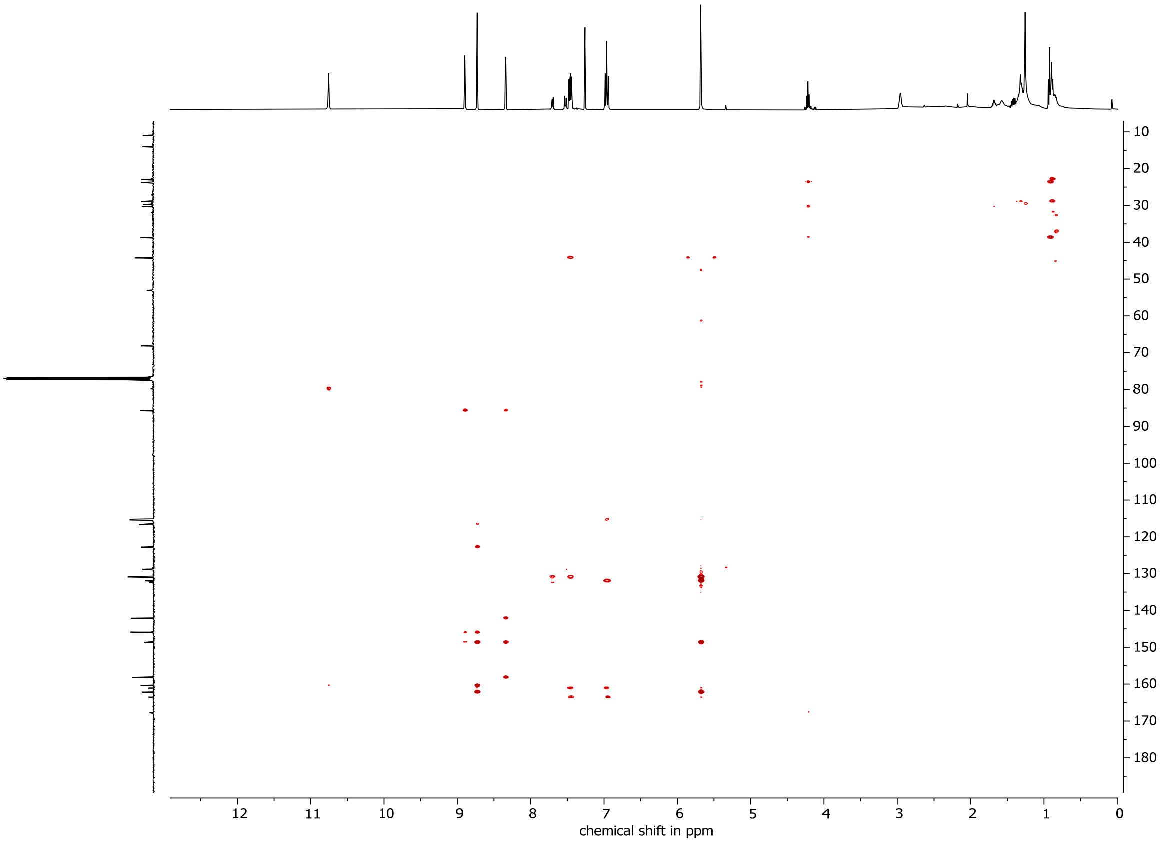

Figure S37. HMBC (^{1}H, ^{13}C) NMR spectrum of compound **3*m*** in $CDCl_3$.

Compound 3_p

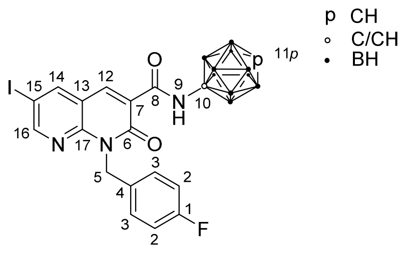

3_p

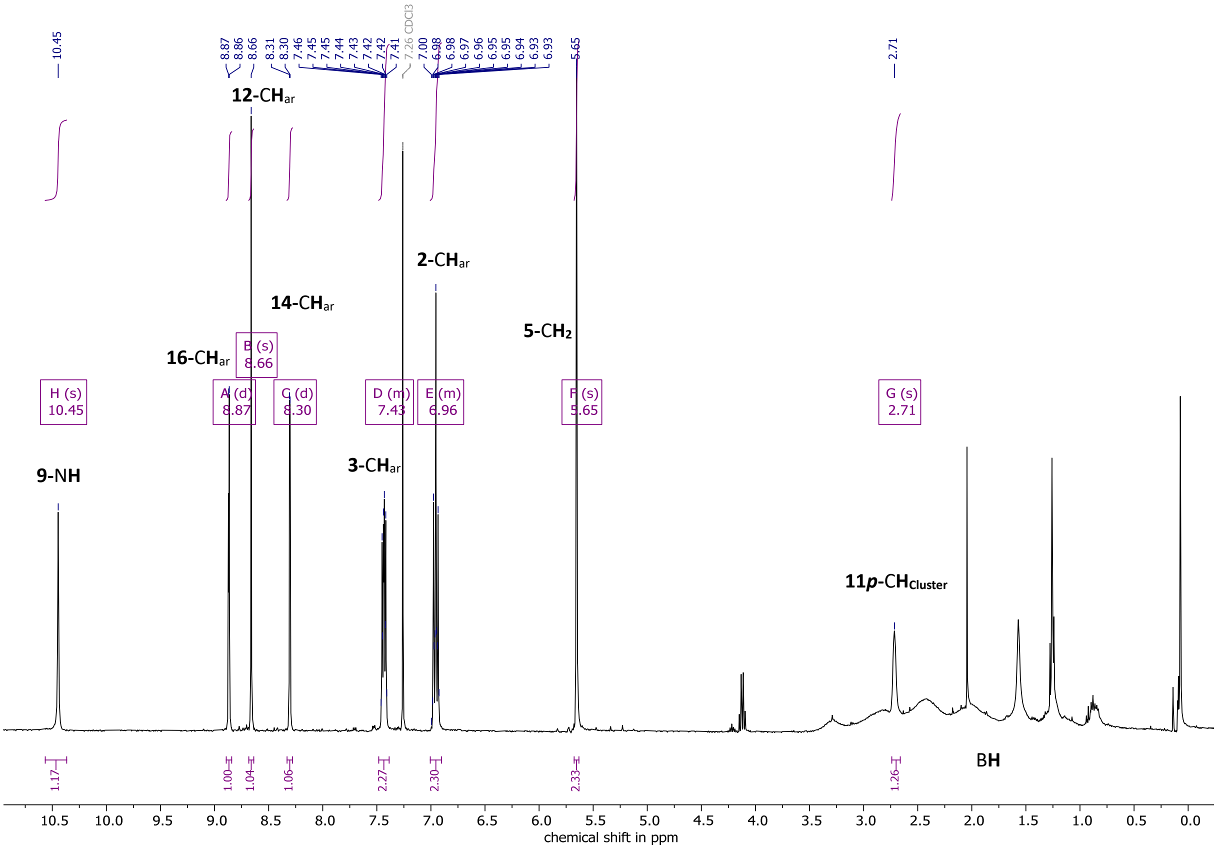

Figure S38. ^{1}H NMR spectrum of compound 3_p in $CDCl_3$.

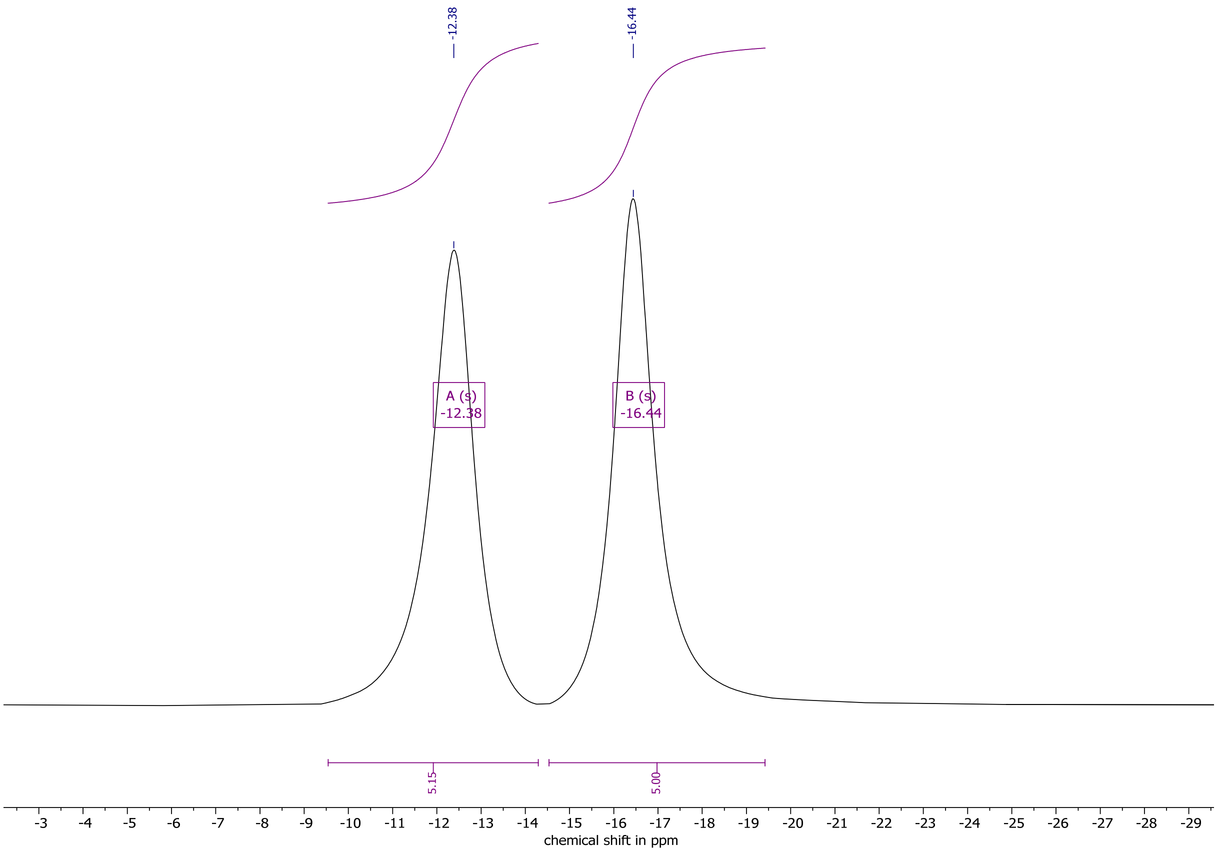

Figure S39. $^{11}B\{^1H\}$ NMR spectrum of compound **3*p*** in $CDCl_3$.

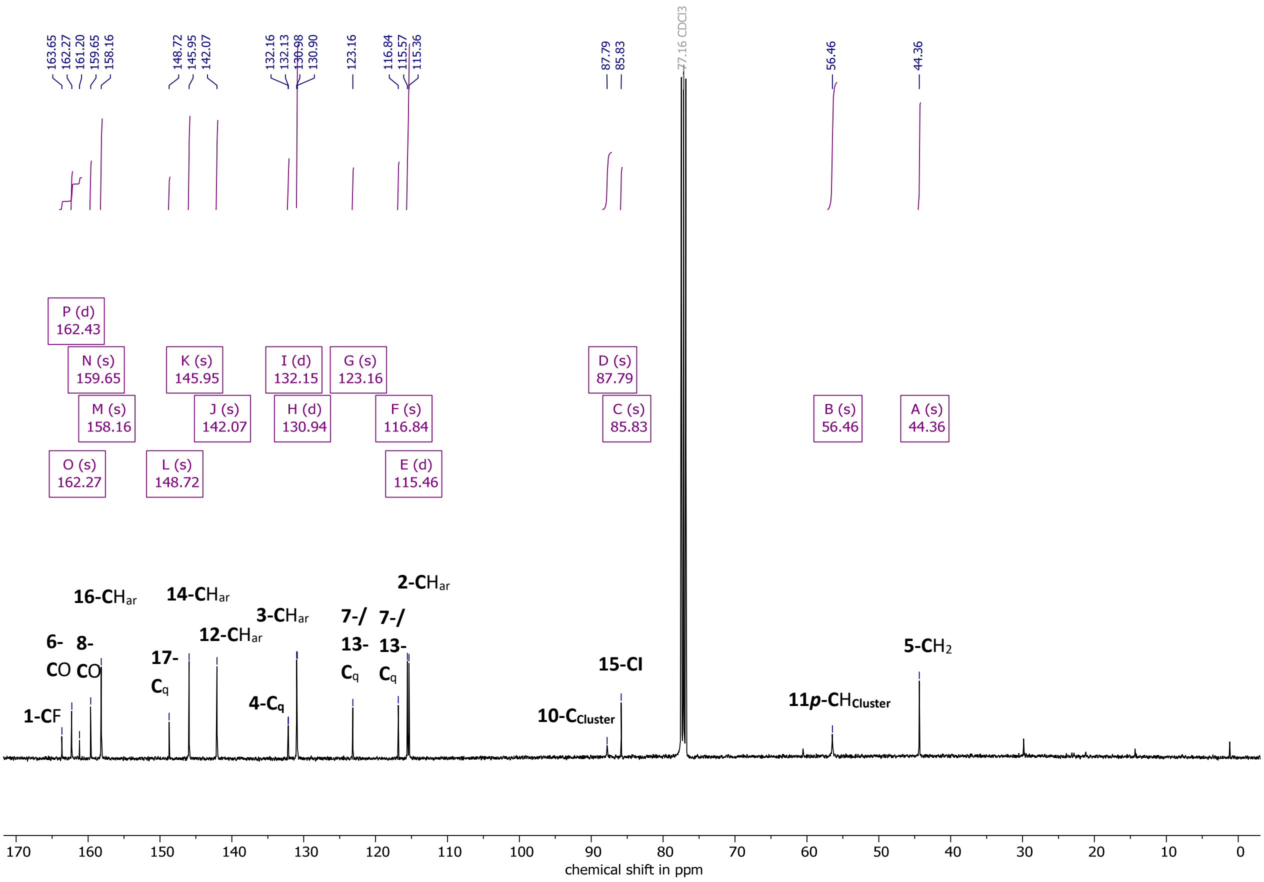

Figure S40. $^{13}C\{^1H\}$ NMR spectrum of compound **3*p*** in $CDCl_3$.

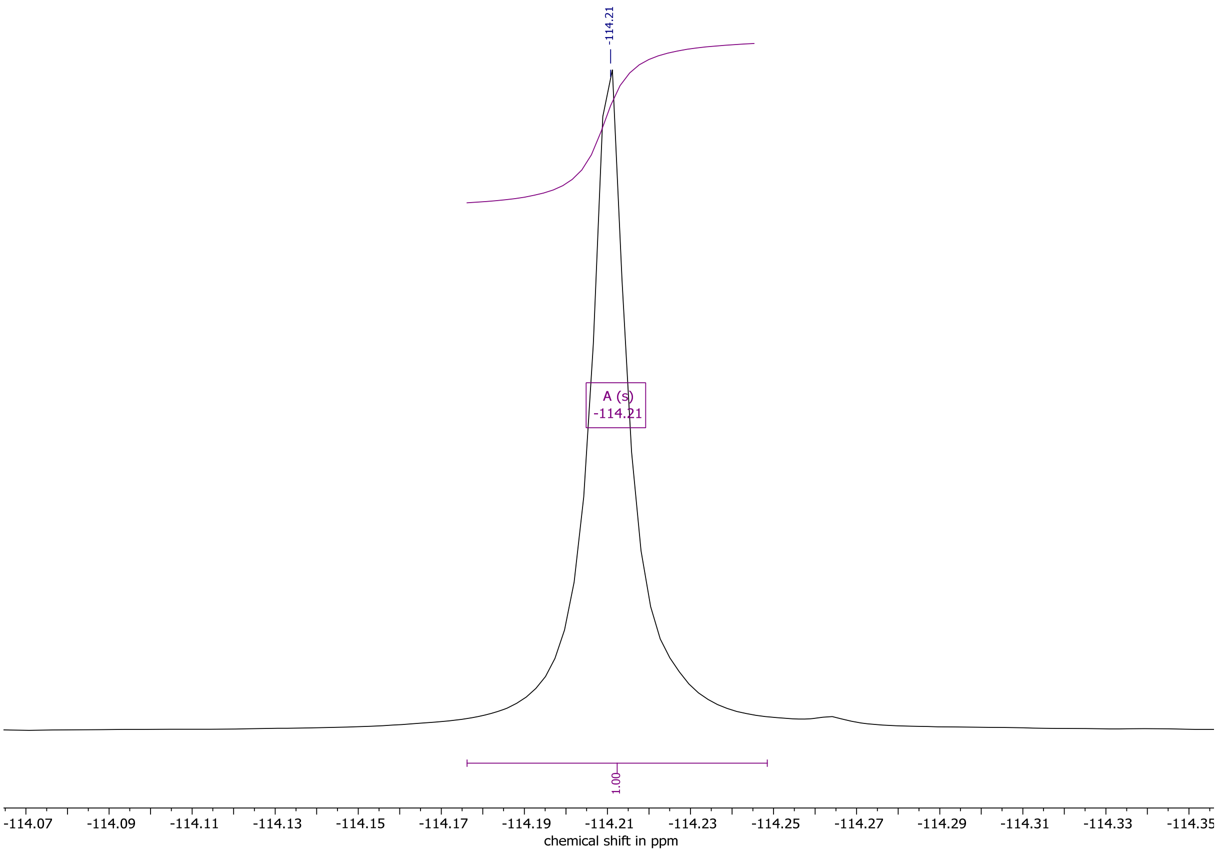

Figure S41. $^{19}F\{^{1}H\}$ NMR spectrum of compound **3*p*** in $CDCl_3$.

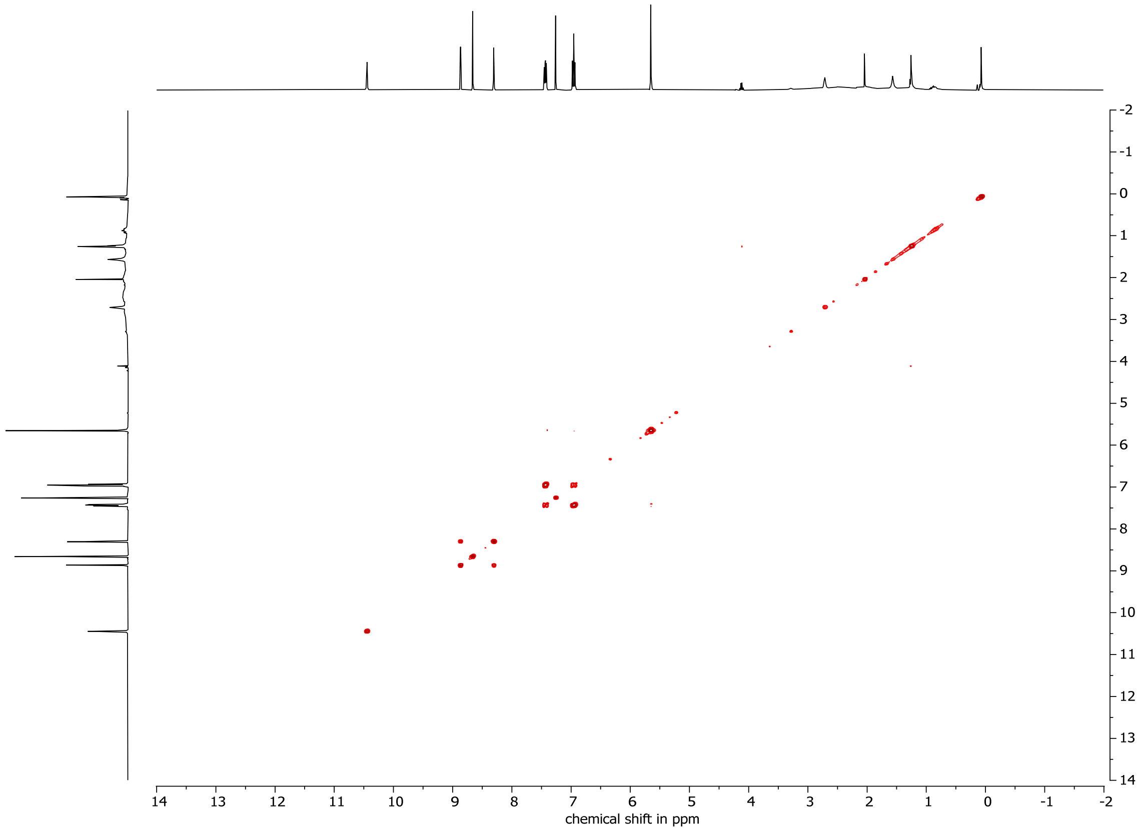

Figure S42. COSY (^{1}H, ^{1}H) NMR spectrum of compound **3*p*** in $CDCl_3$.

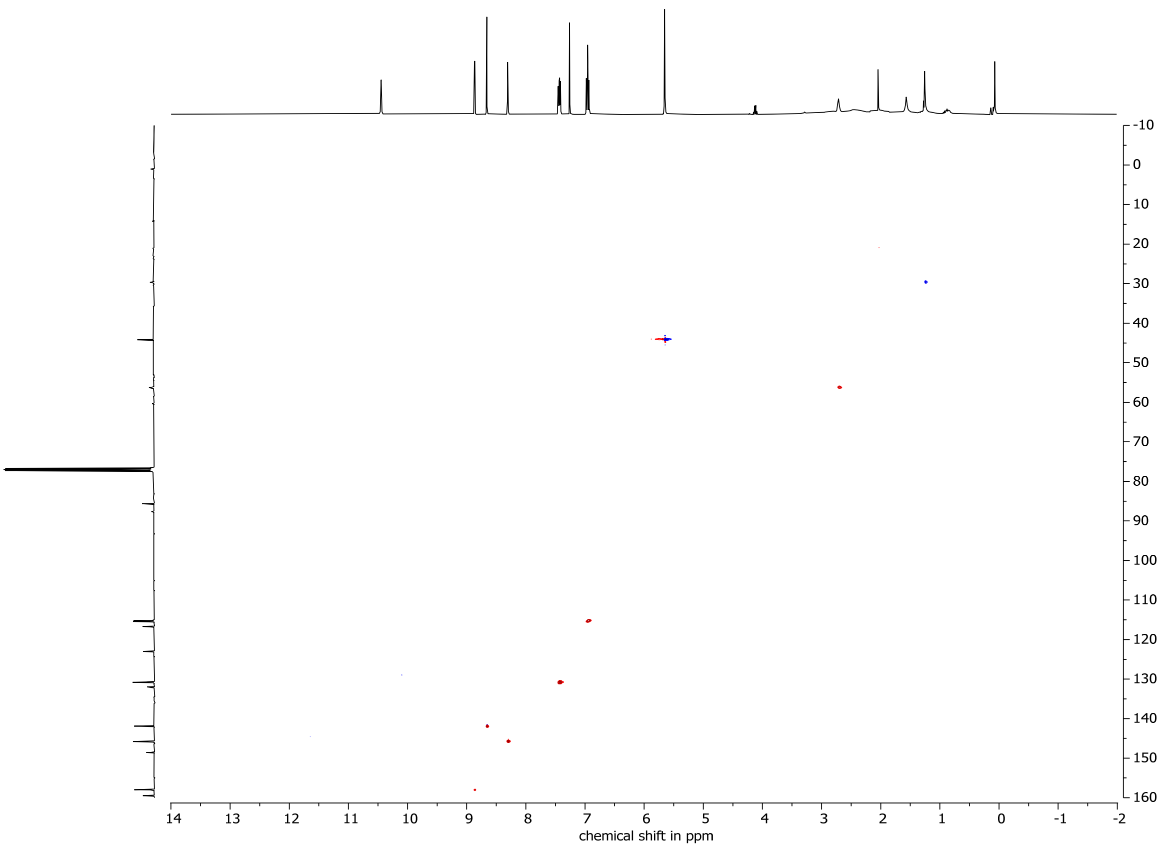

Figure S43. HSQC (^1H, ^{13}C) NMR spectrum of compound **3*p*** in $CDCl_3$.

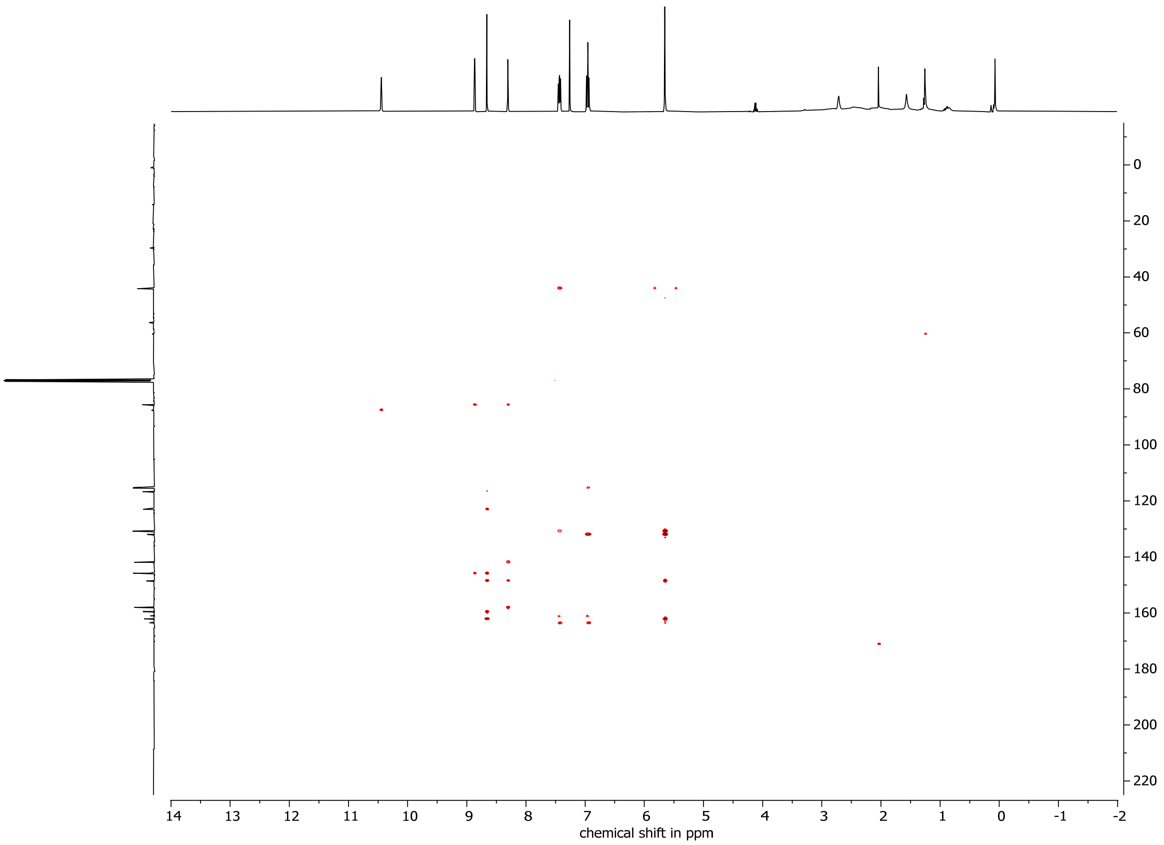

Figure S44. HMBC (^1H, ^{13}C) NMR spectrum of compound **3*p*** in $CDCl_3$.

Compound **4**

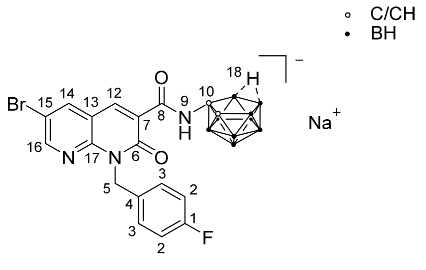

4

nido-ClusterCH = 11*n*

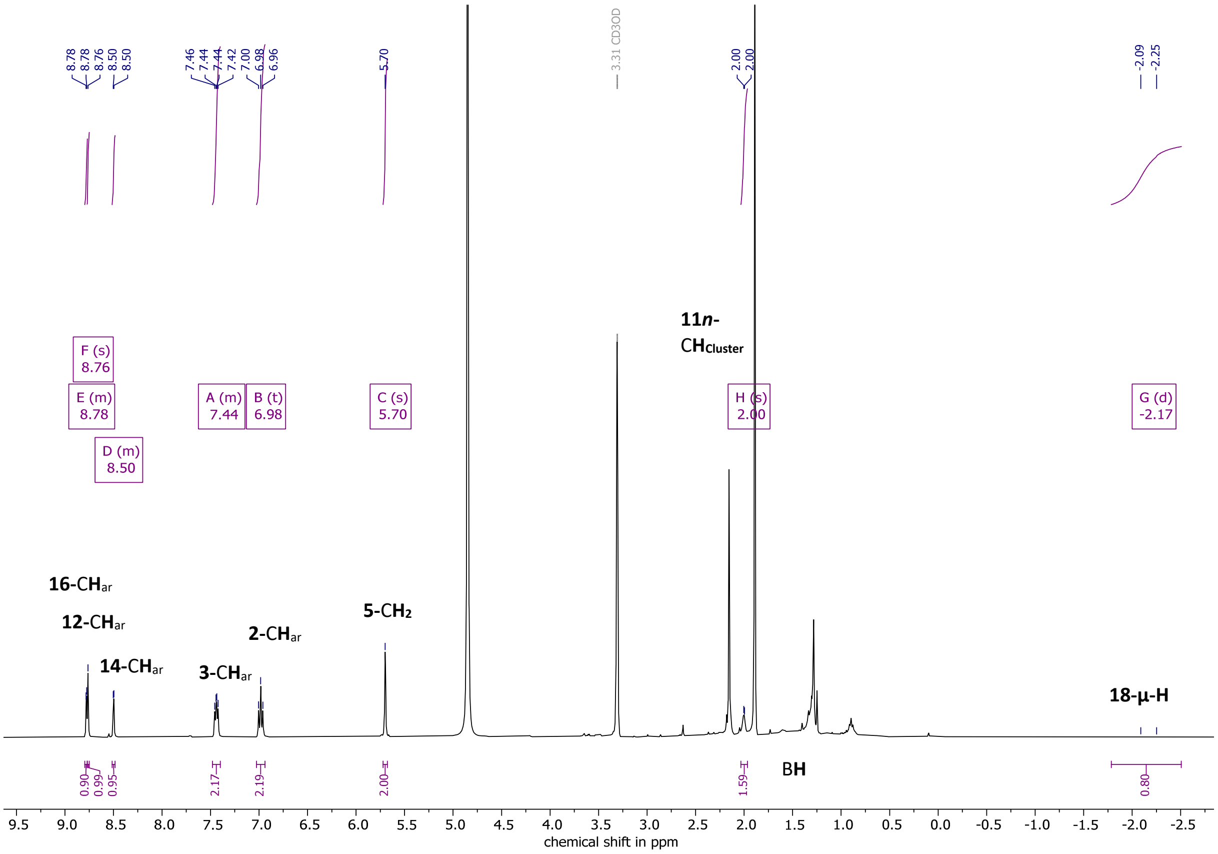

Figure S45. ^{1}H NMR spectrum of compound **4** in CD_3OD full spectrum.

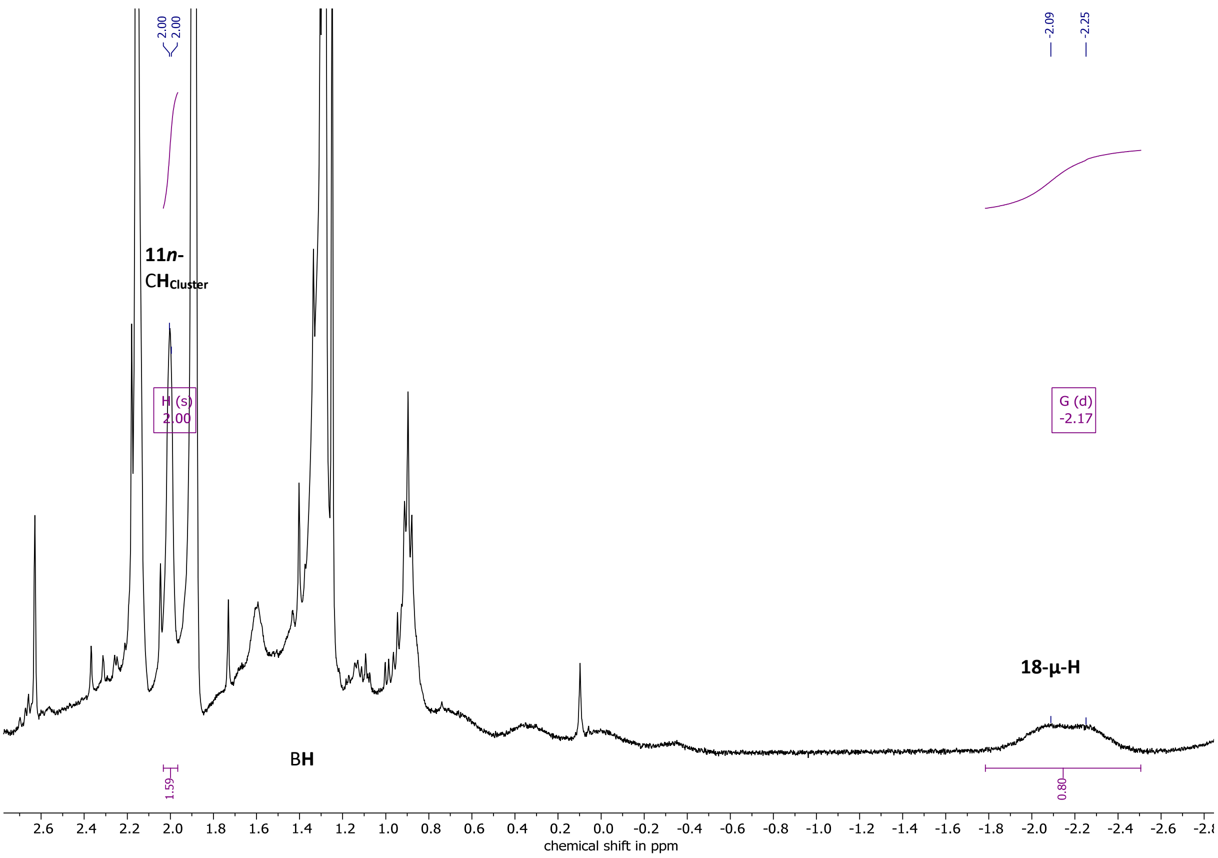

Figure S46. ^{1}H NMR spectrum of compound **4** in CD_3OD (magnified spectrum, zoomed in).

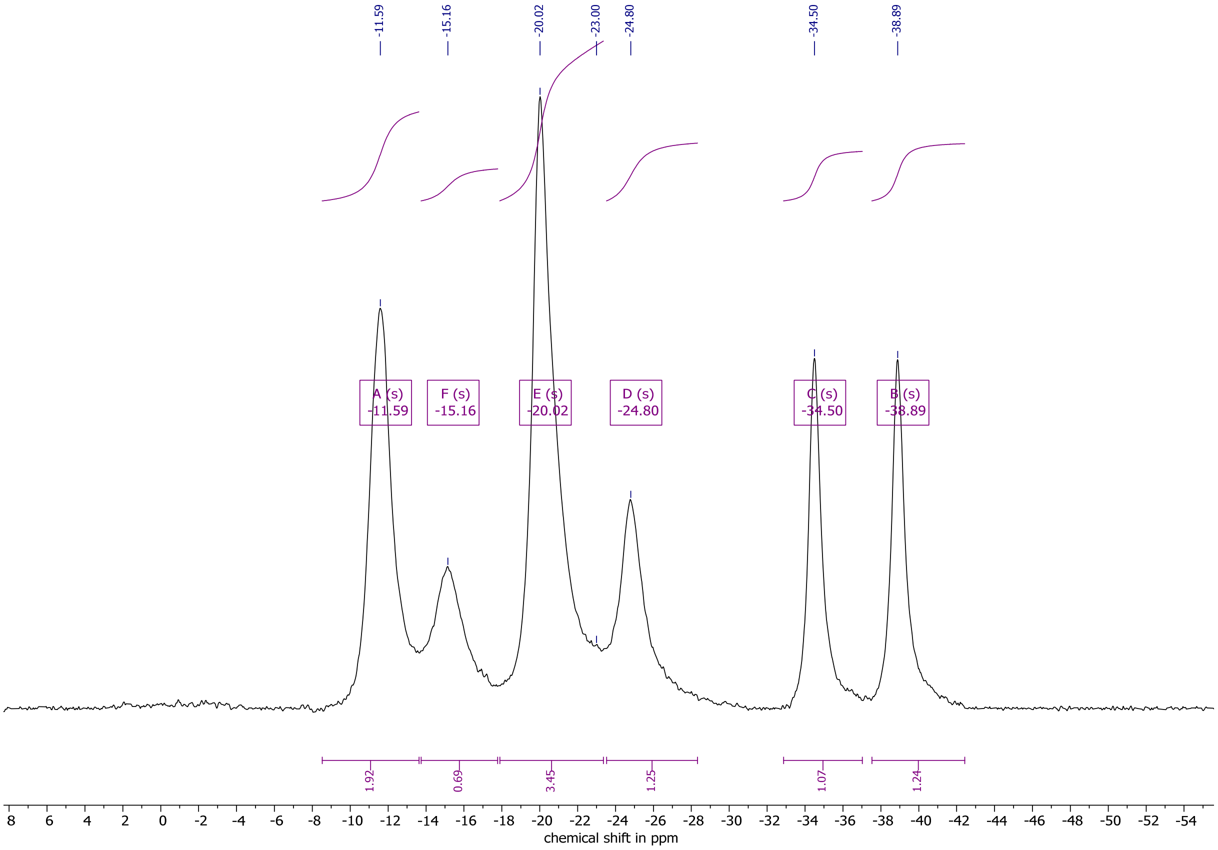

Figure S47. $^{11}B\{^{1}H\}$ NMR spectrum of compound **4** in CD_3OD.

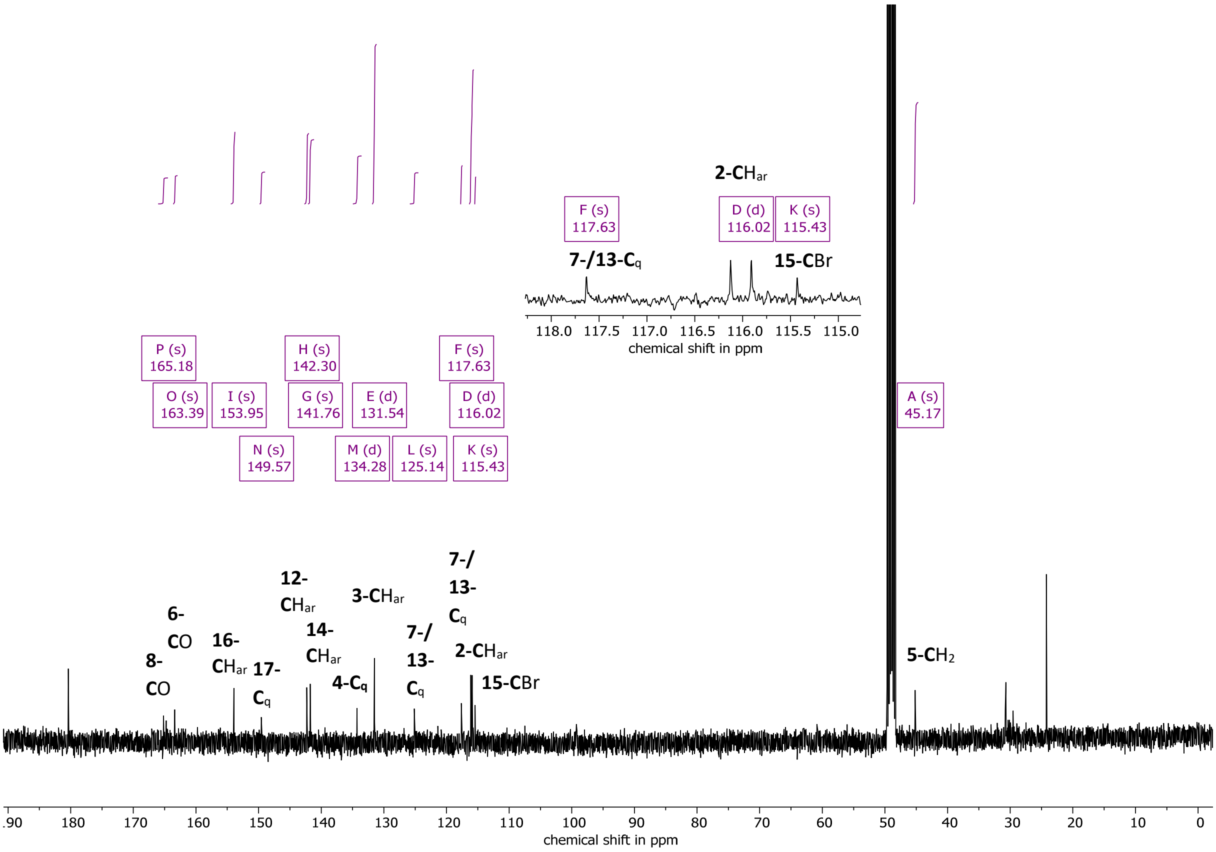

Figure S48. $^{13}C\{^{1}H\}$ NMR spectrum of compound **4** in CD_3OD.

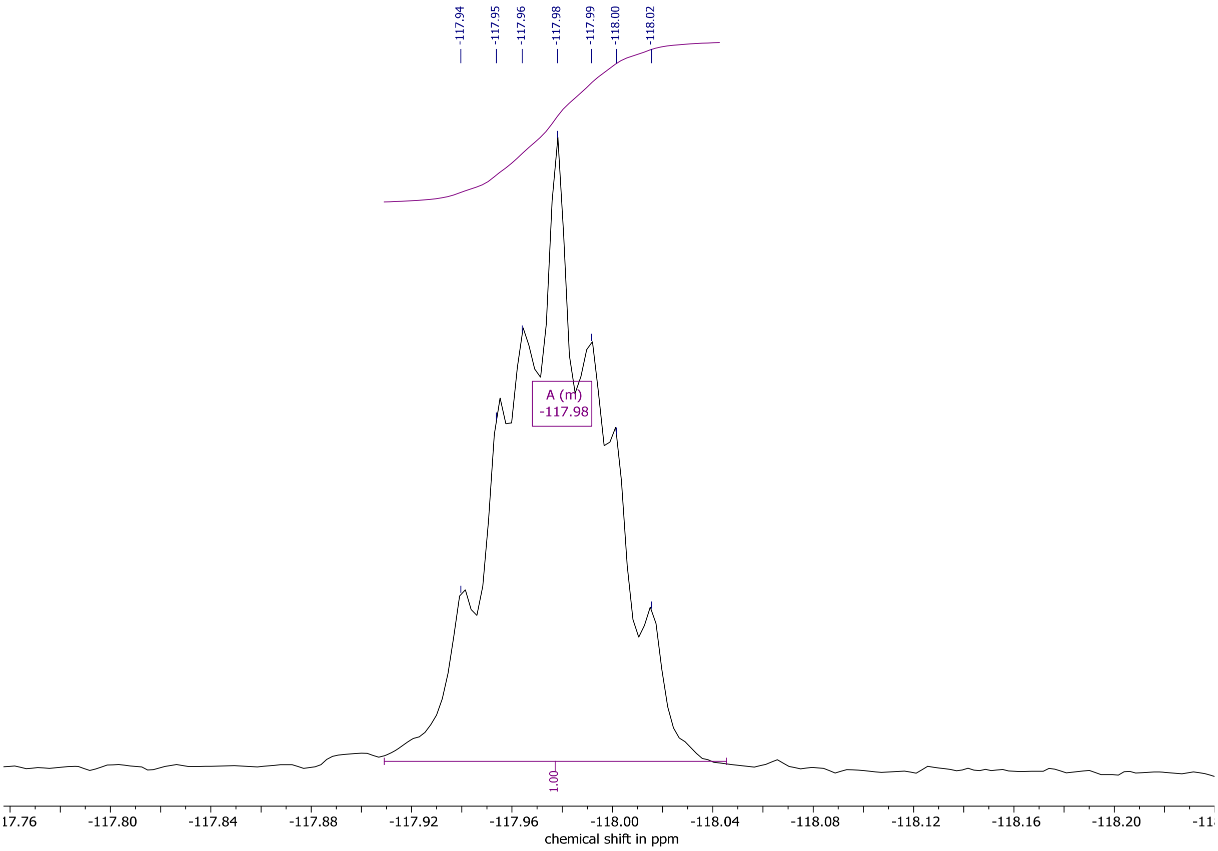

Figure S49. $^{19}F\{^{1}H\}$ NMR spectrum of compound **4** in CD_3OD.

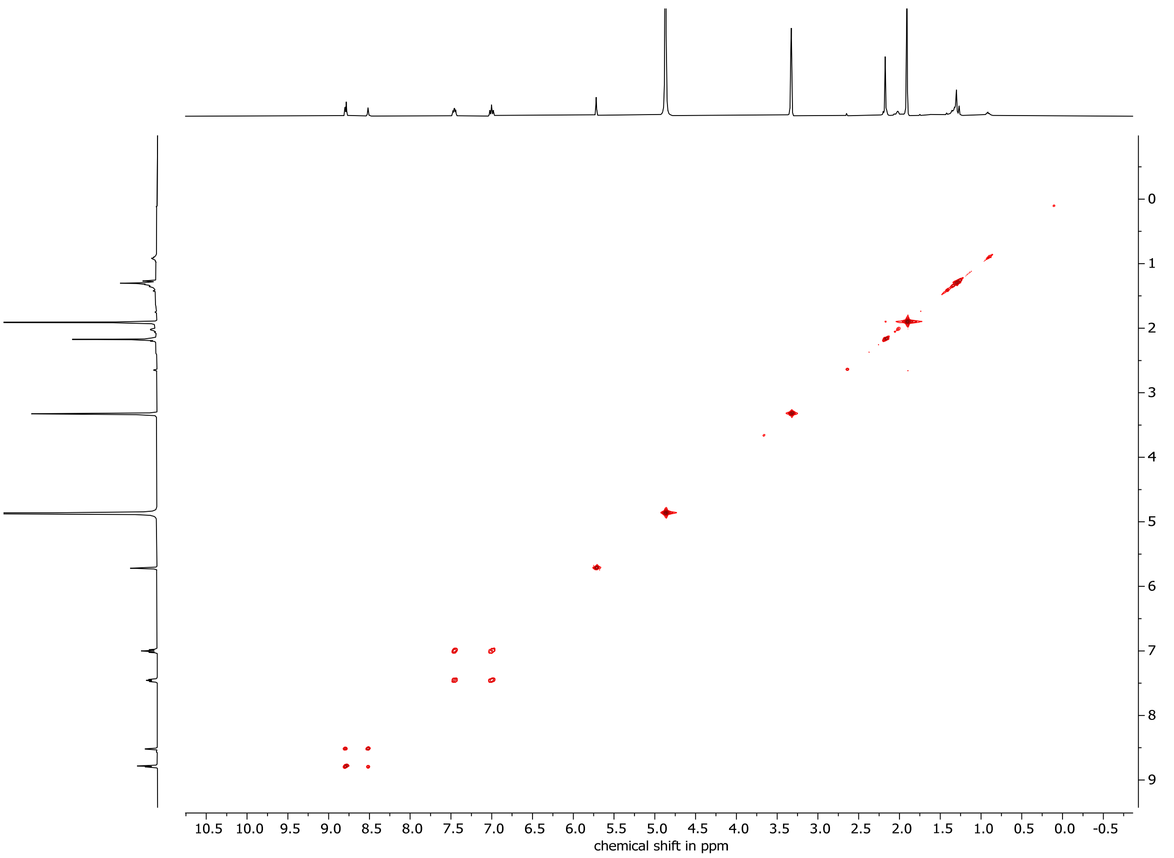

Figure S50. COSY (^{1}H, ^{1}H) NMR spectrum of compound **4** in CD_3OD.

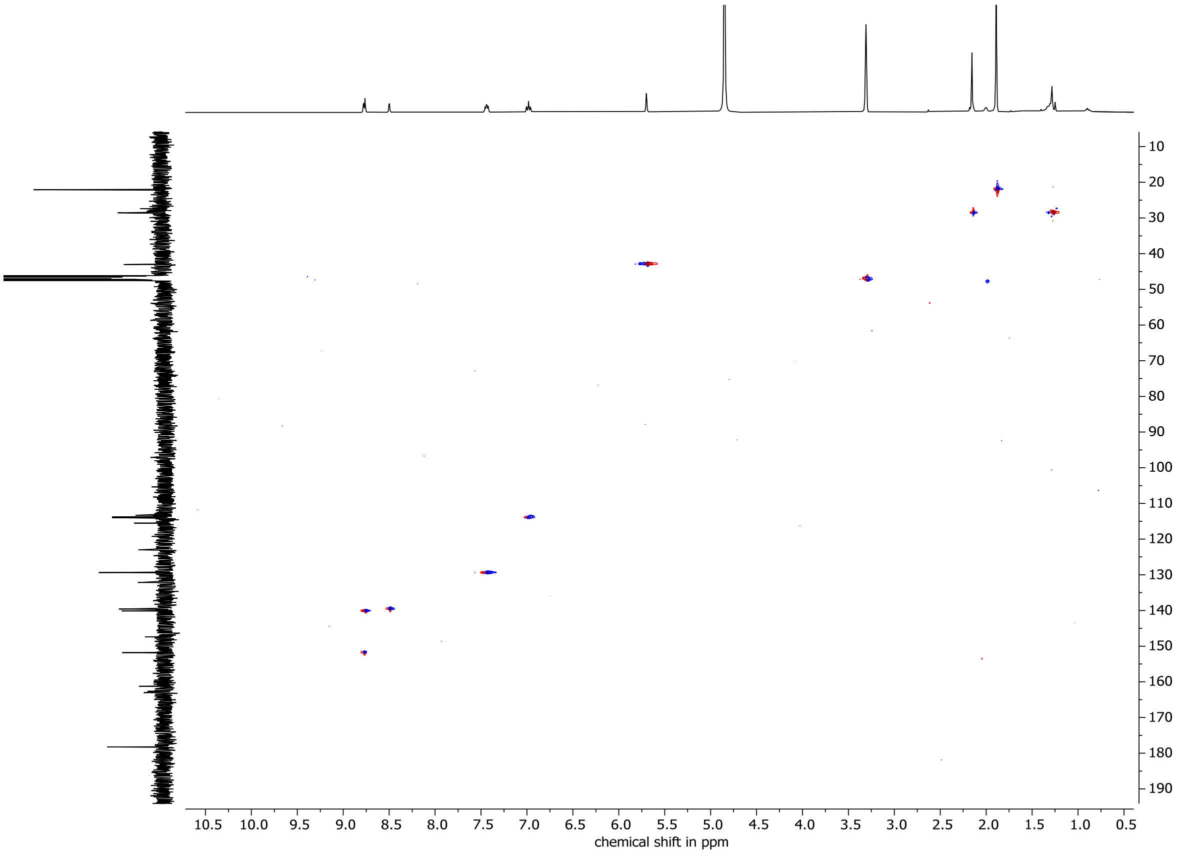

Figure S51. HSQC (^{1}H, ^{13}C) NMR spectrum of compound **4** in CD_3OD.

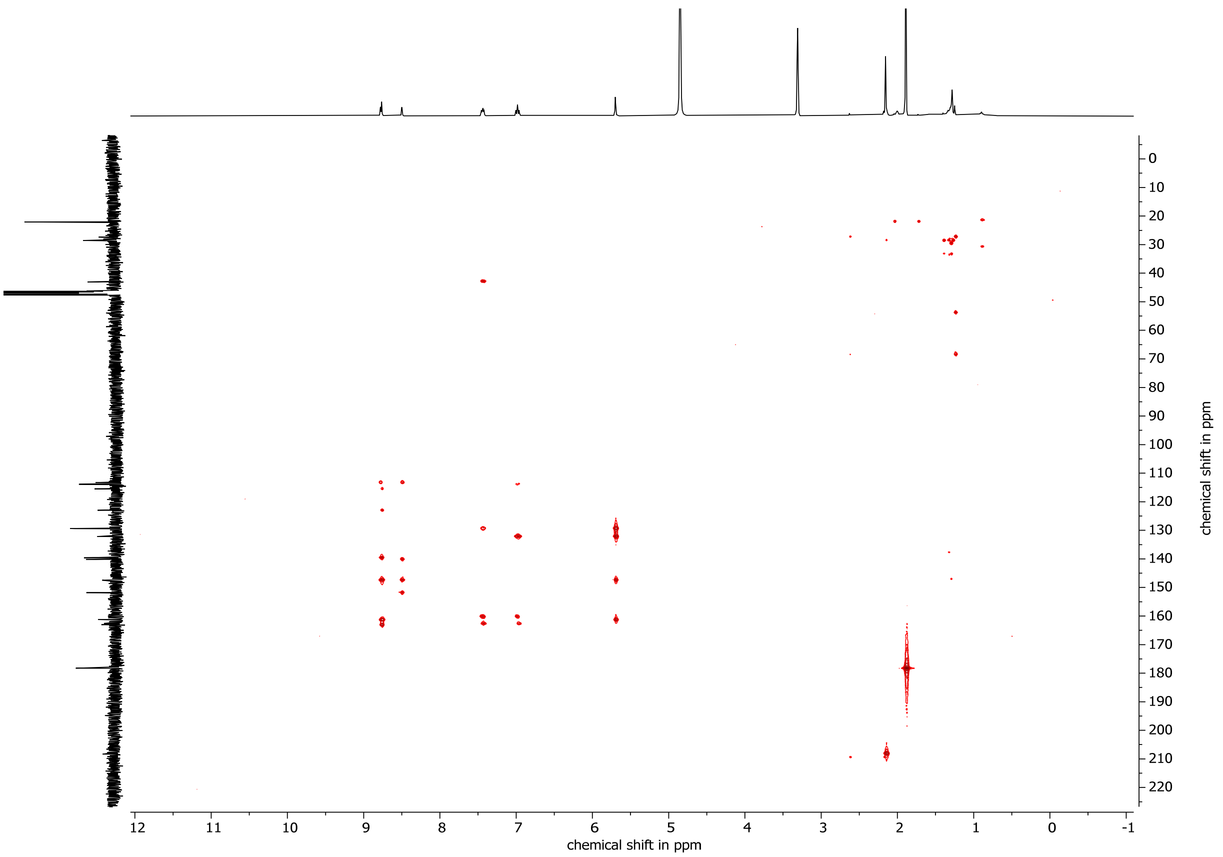

Figure S52. HMBC (^{1}H, ^{13}C) NMR spectrum of compound **4** in CD_3OD.

Compound **5**

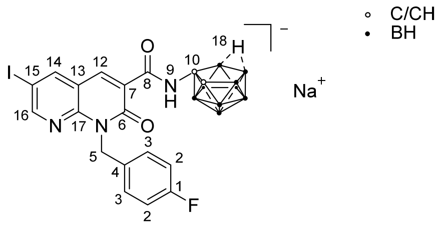

5

nido-ClusterCH = 11*n*

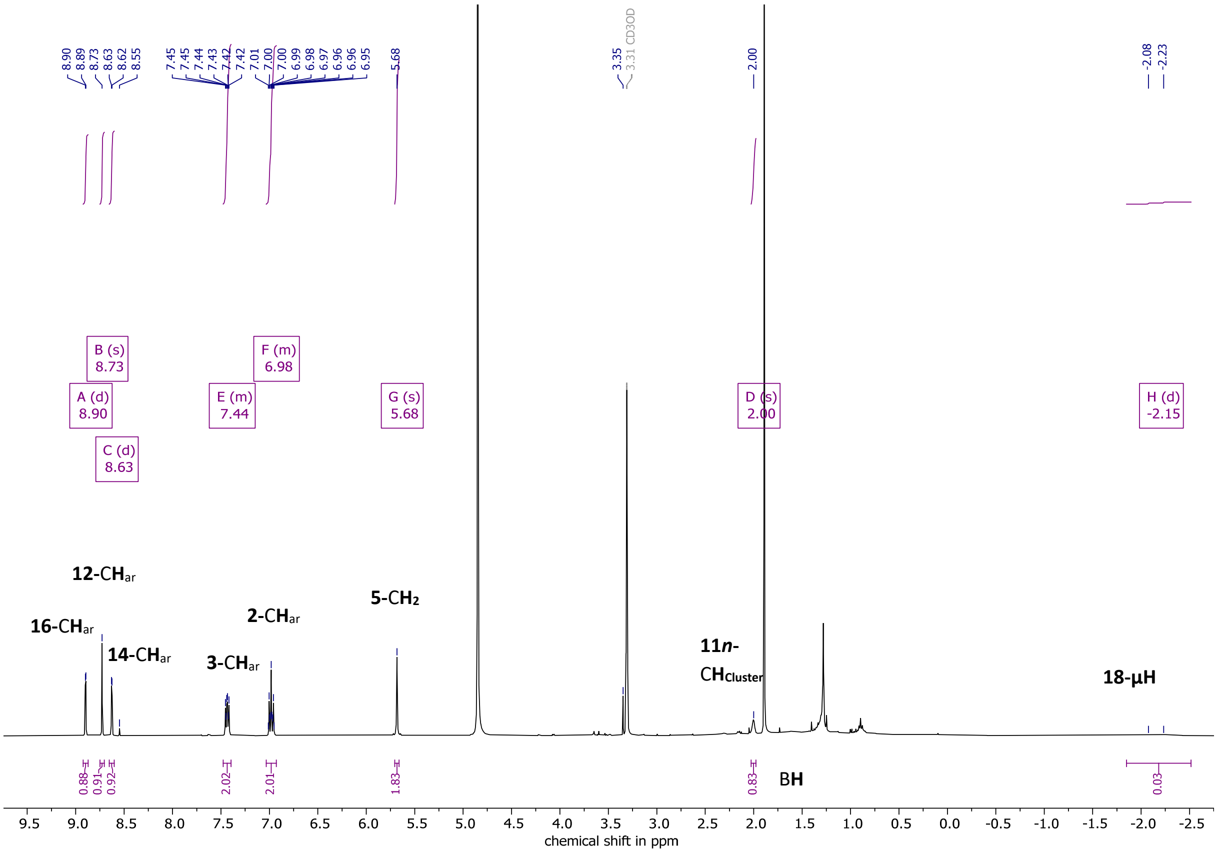

Figure S53. ^{1}H NMR spectrum of compound **5** in CD_3OD full spectrum.

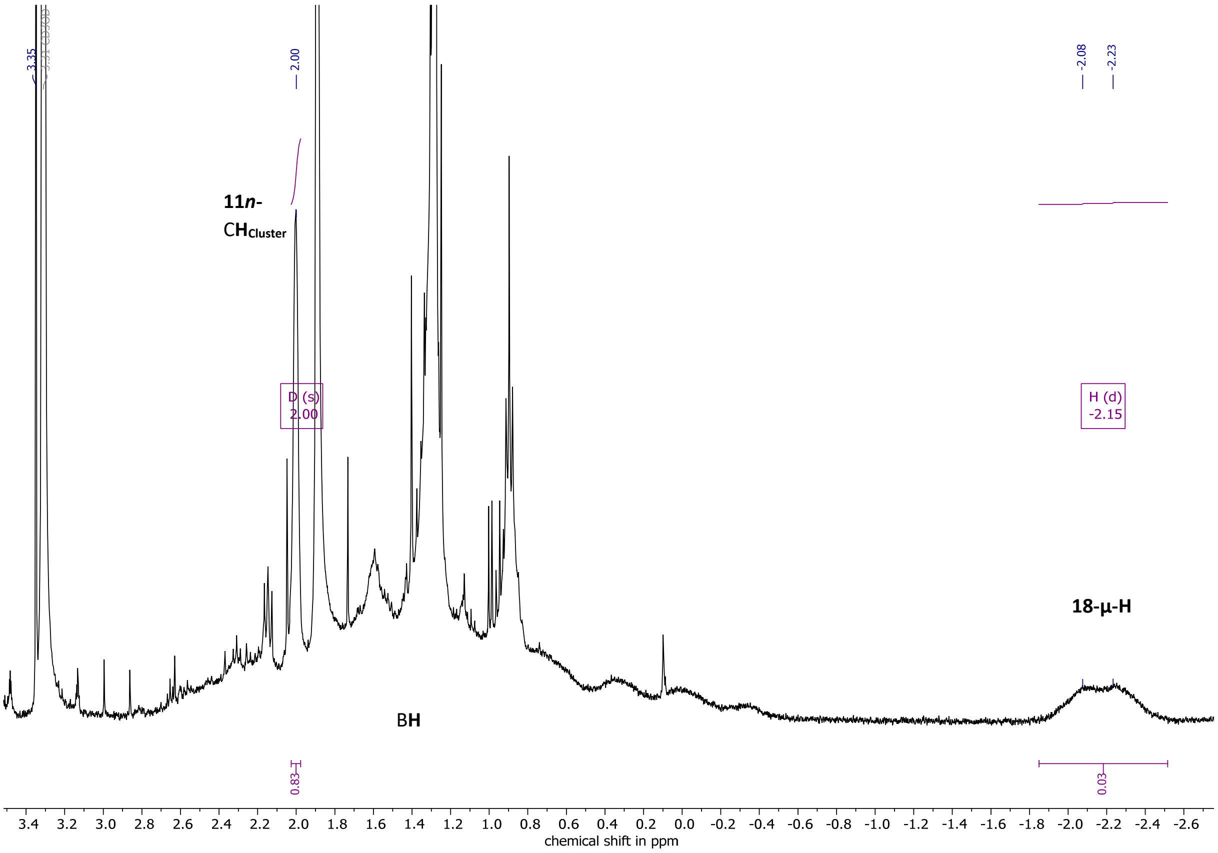

Figure S54. ^{1}H NMR spectrum of compound **5** in CD_3OD (magnified spectrum, zoomed in).

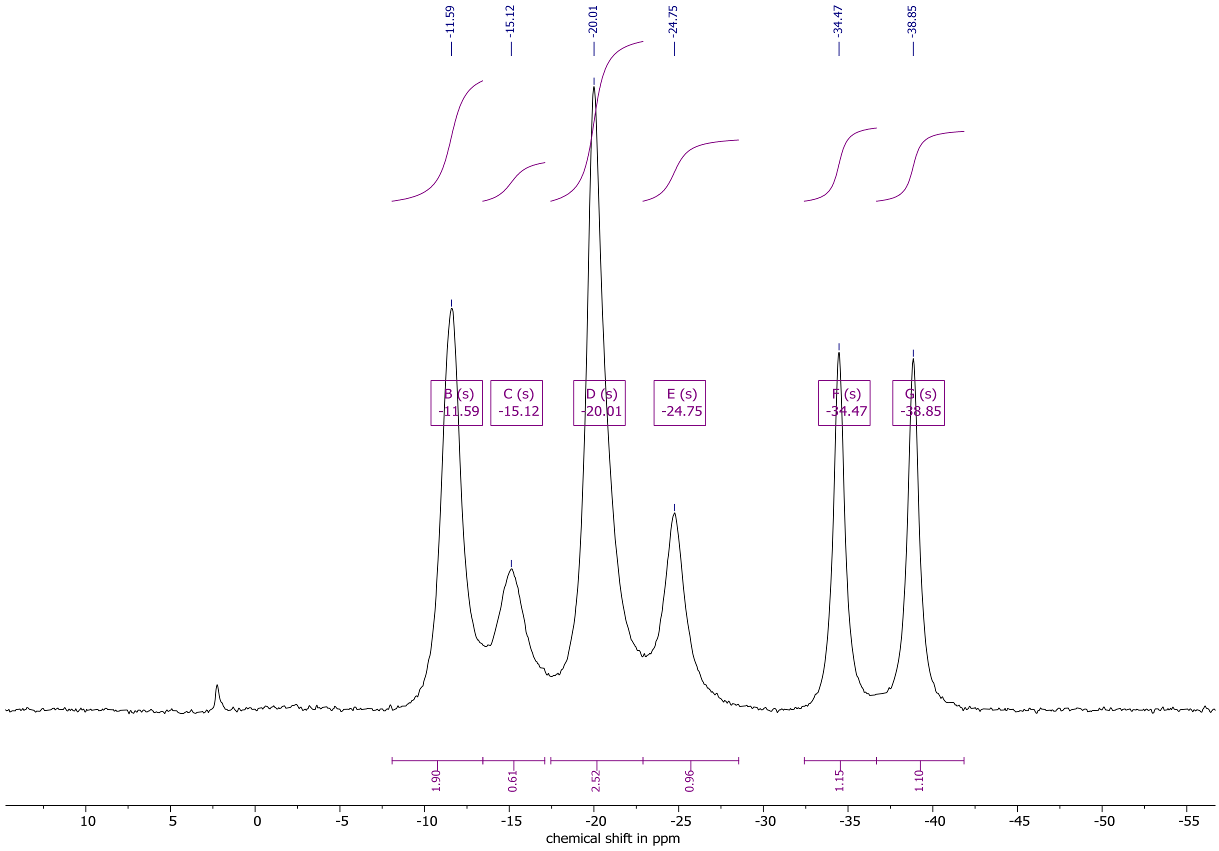

Figure S55. $^{11}B\{^{1}H\}$ NMR spectrum of compound **5** in CD_3OD.

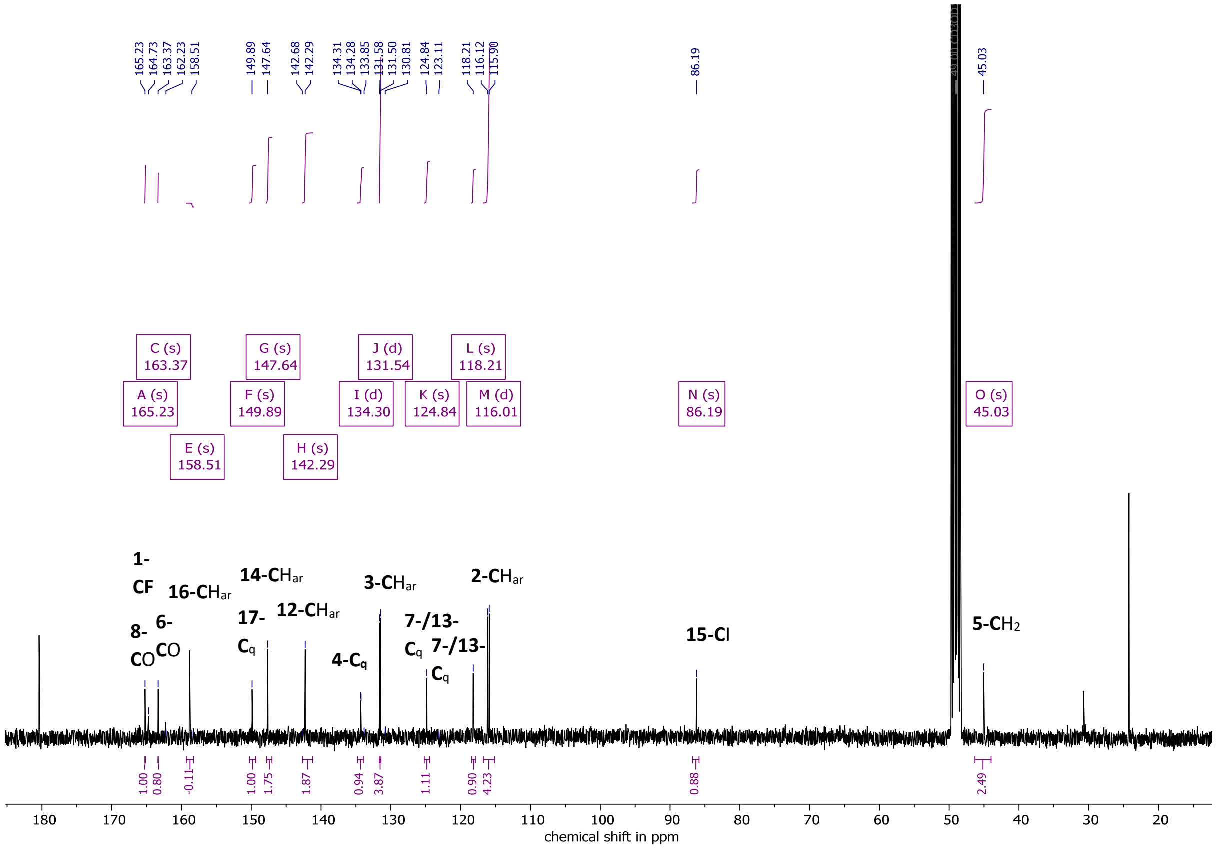

Figure S56. $^{13}C\{^{1}H\}$ NMR spectrum of compound **5** in CD_3OD.

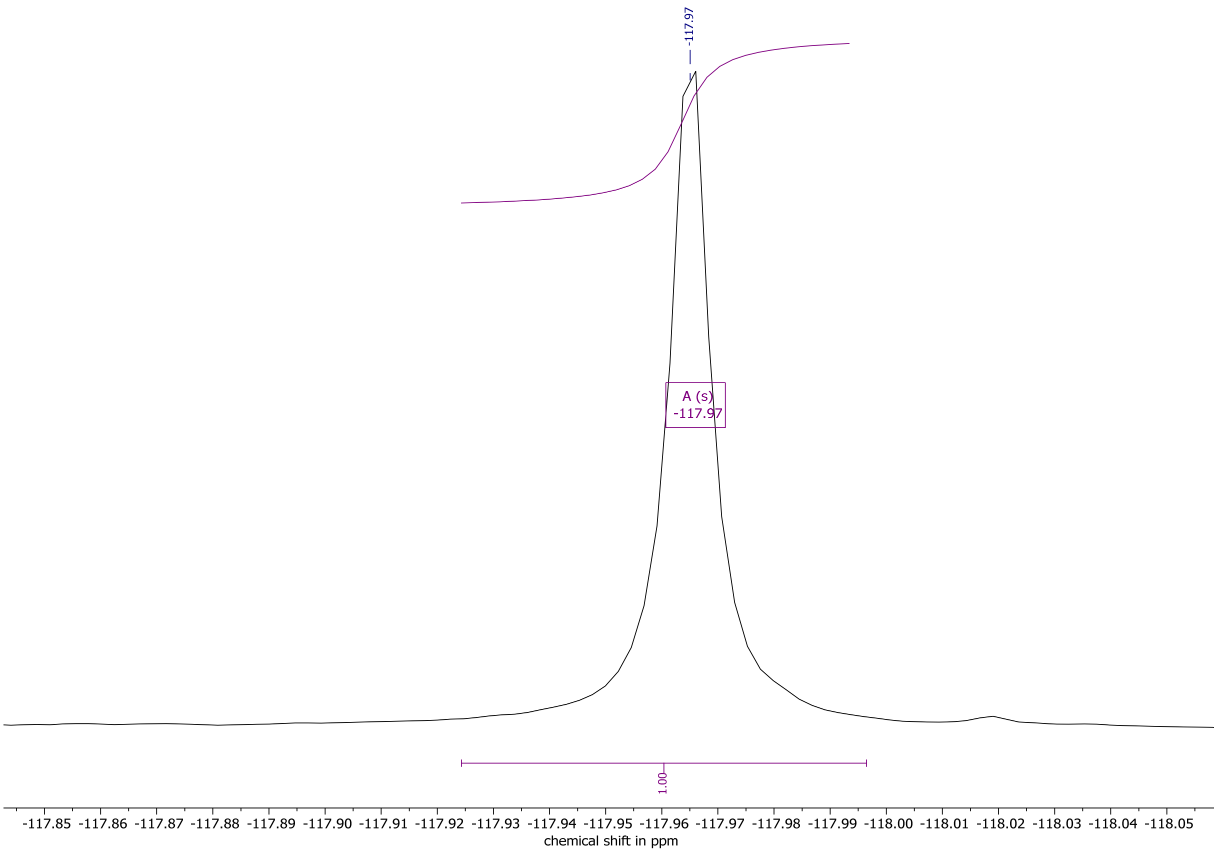

Figure S57. $^{19}F\{^1H\}$ NMR spectrum of compound **5** in CD_3OD.

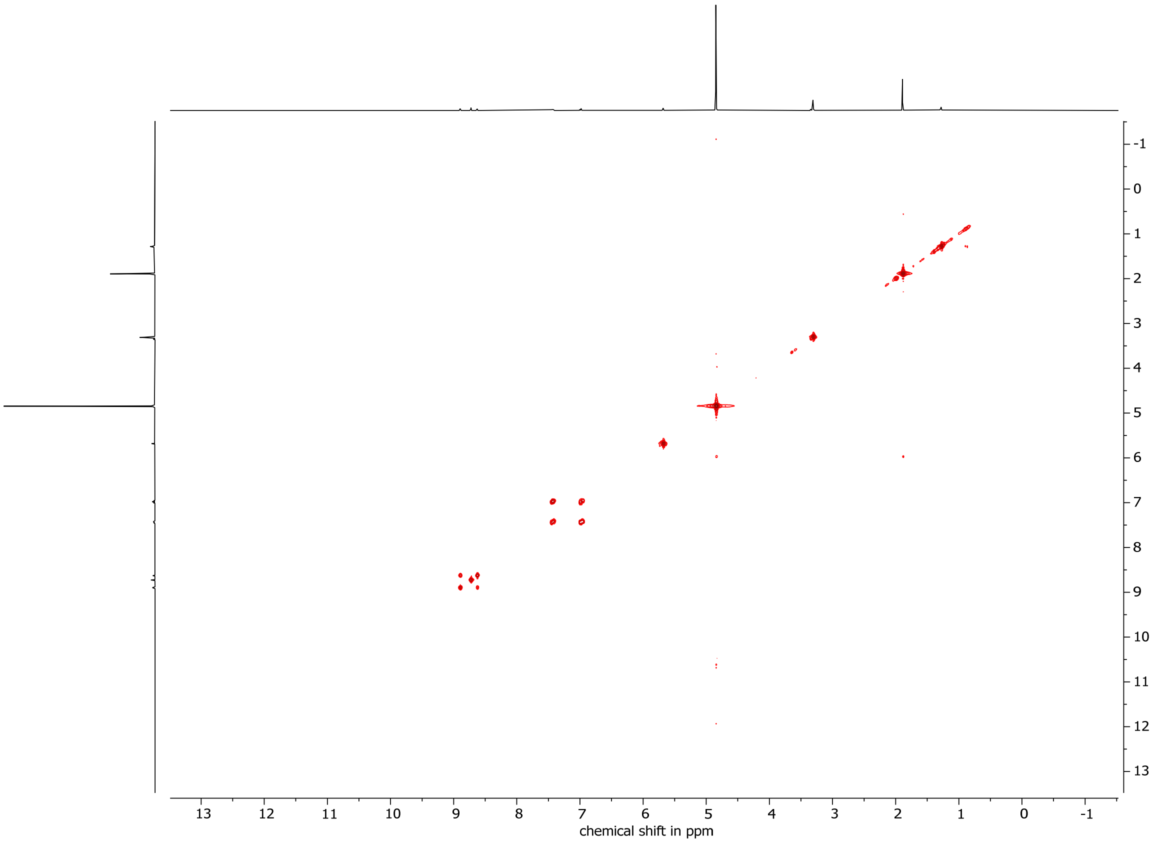

Figure S58. COSY (^1H, ^1H) NMR spectrum of compound **5** in CD_3OD.

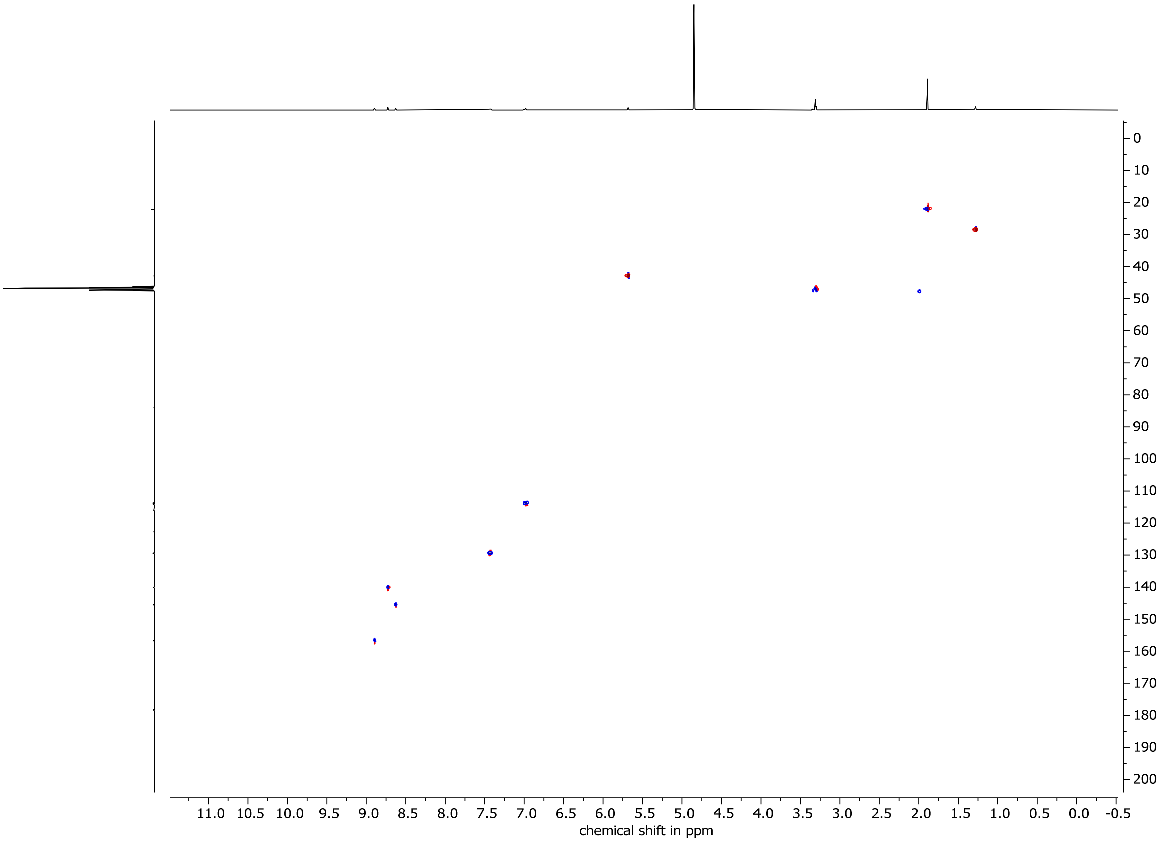

Figure S59. HSQC (^{1}H, ^{13}C) NMR spectrum of compound **5** in CD_3OD.

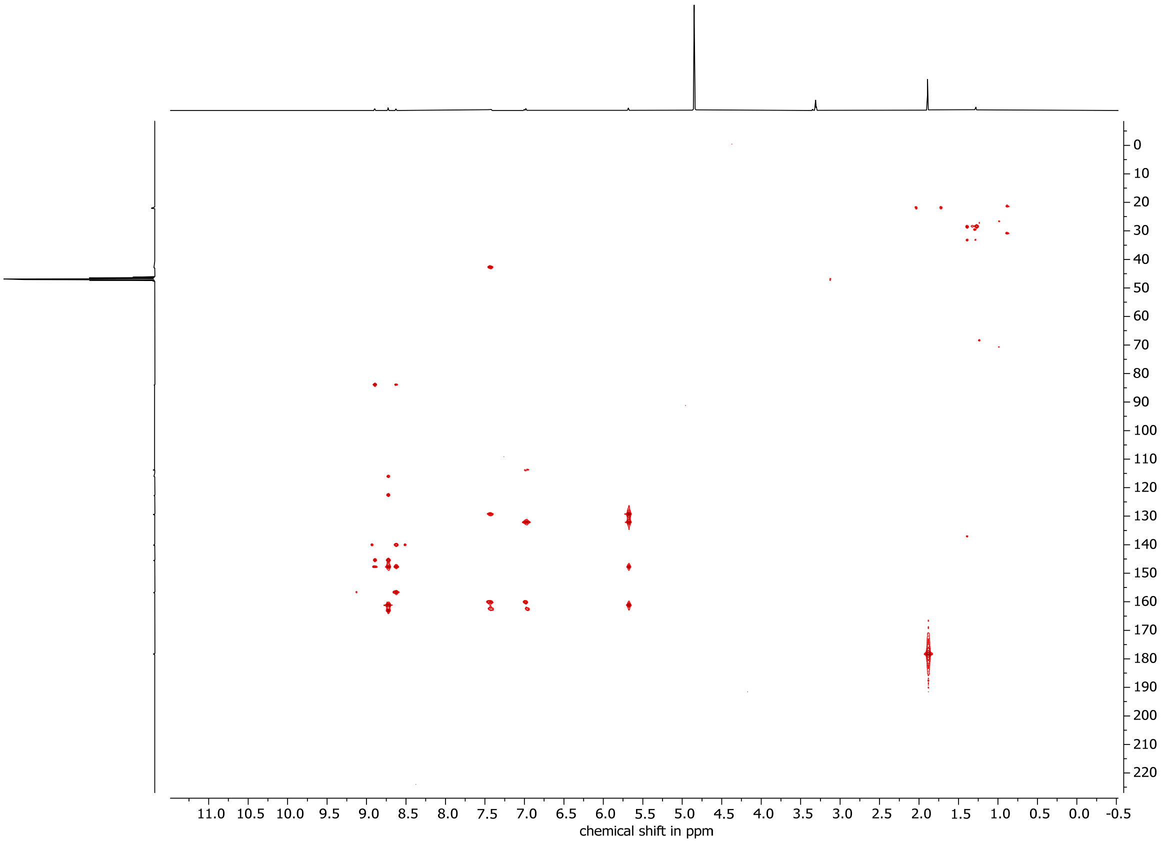

Figure S60. HMBC (^{1}H, ^{13}C) NMR spectrum of compound **5** in CD_3OD.

3 HR-ESI Mass Spectra of Compounds $2_{o,m,p}$, $3_{o,m,p}$, 4, and 5

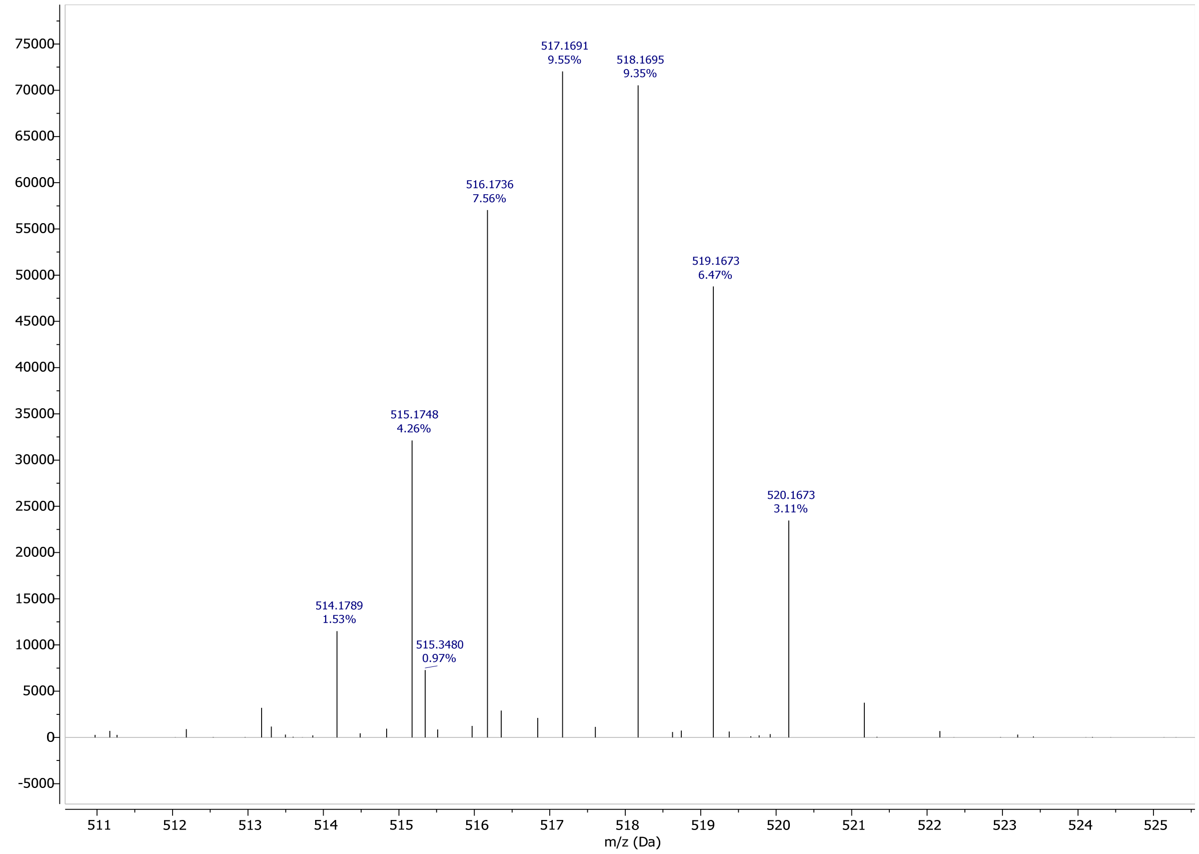

Figure S61. HRMS (ESI–)of compound **2*o*** in CH_3CN.

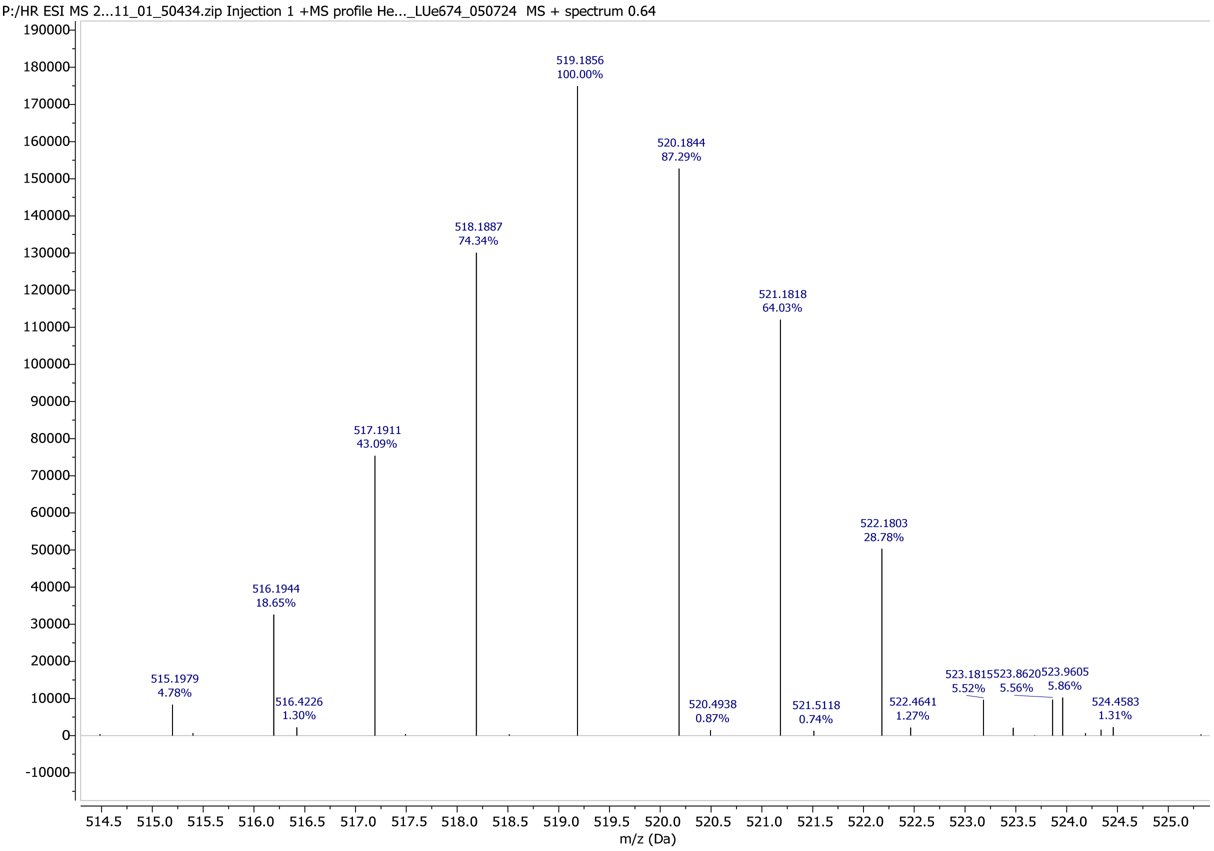

Figure S62. HRMS (ESI+) of compound **2*m*** in CH_3CN.

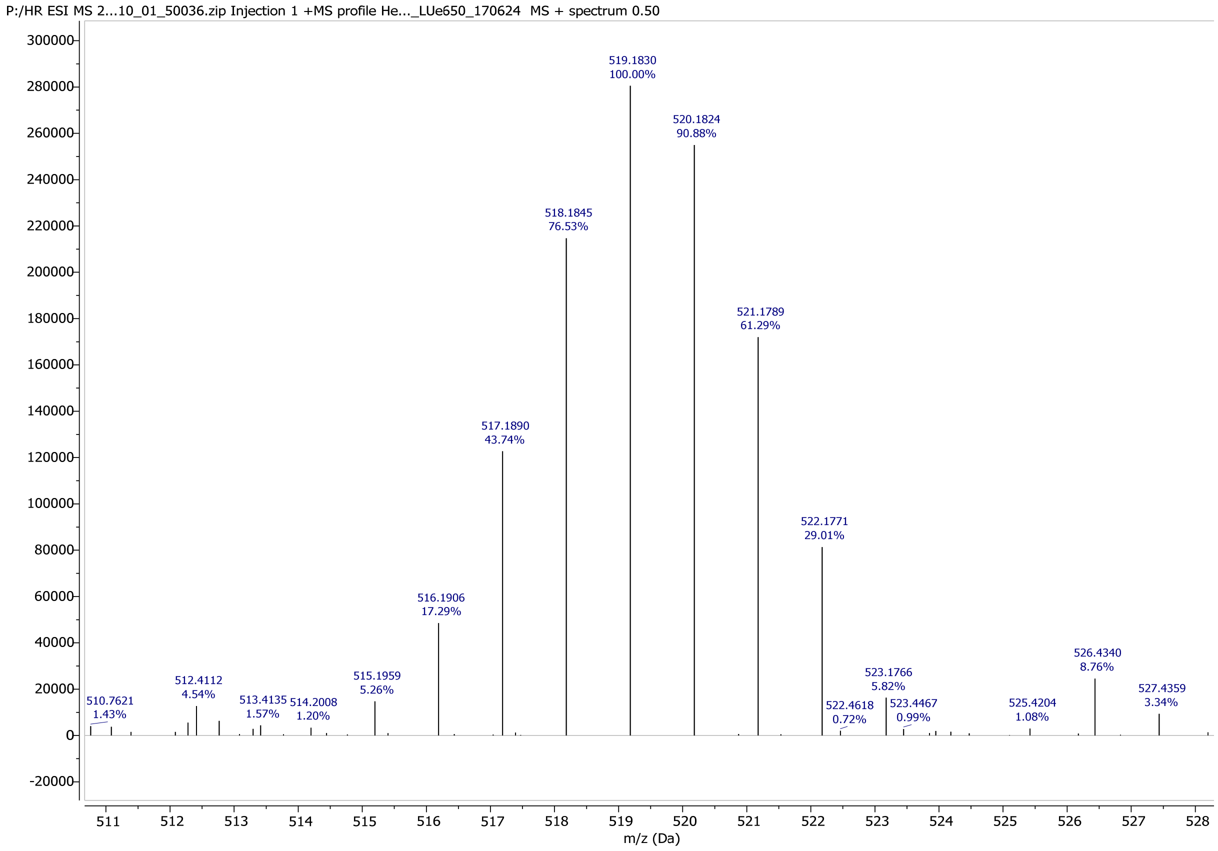

Figure S63. HRMS (ESI+) of compound $\mathbf{2}_{\boldsymbol{p}}$ in CH_3CN.

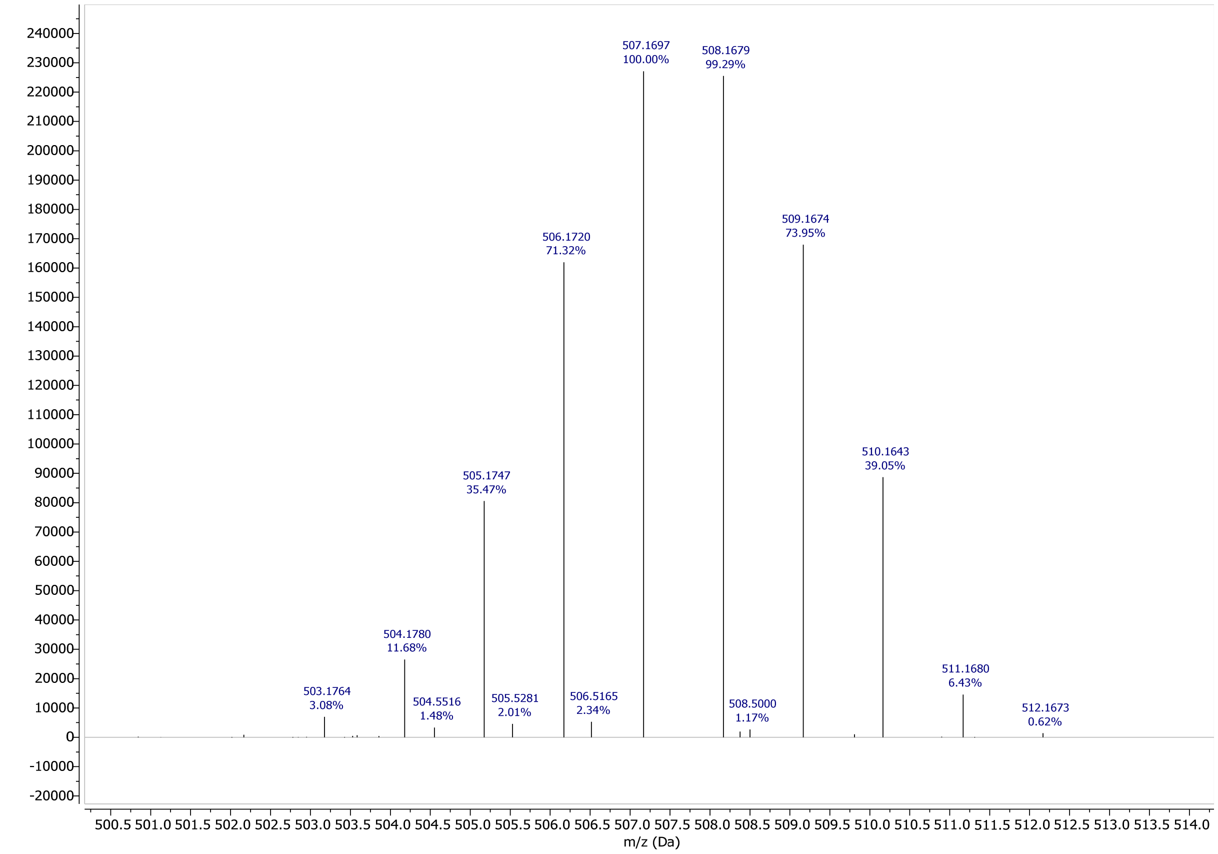

Figure S64. HRMS (ESI–) of compound **4** in MeOH.

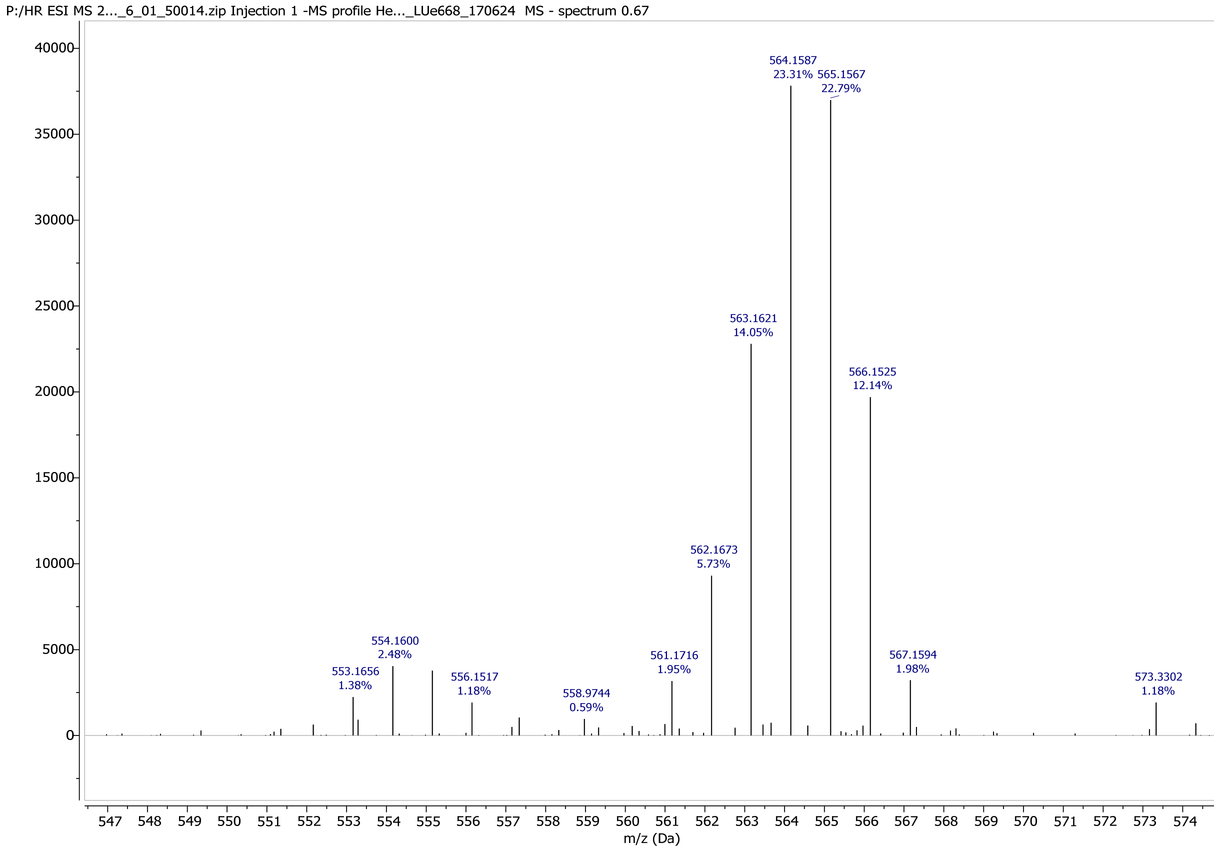

Figure S65. HRMS (ESI–) of compound $\mathbf{3}_{\boldsymbol{o}}$ in CH_3CN.

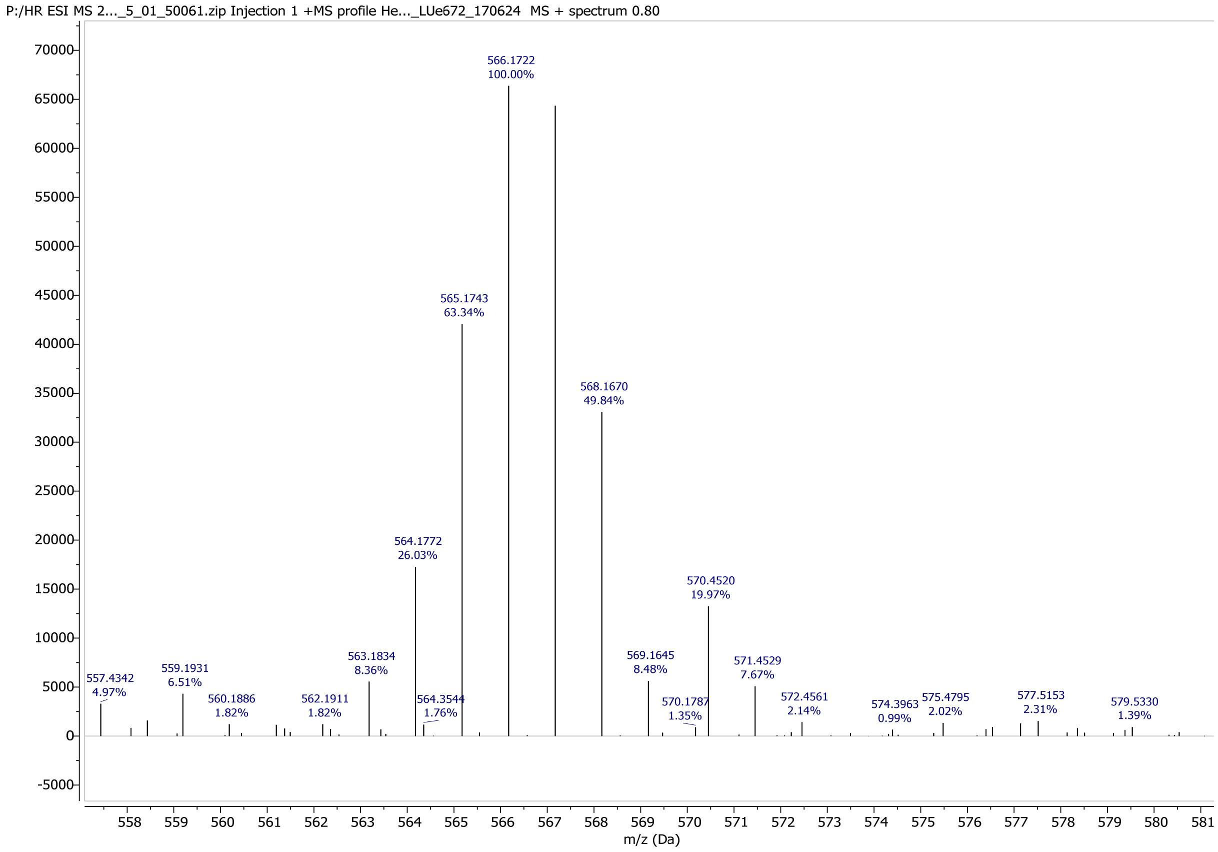

Figure S66. HRMS (ESI+) of compound $\mathbf{3}_{\boldsymbol{m}}$ in CH_3CN.

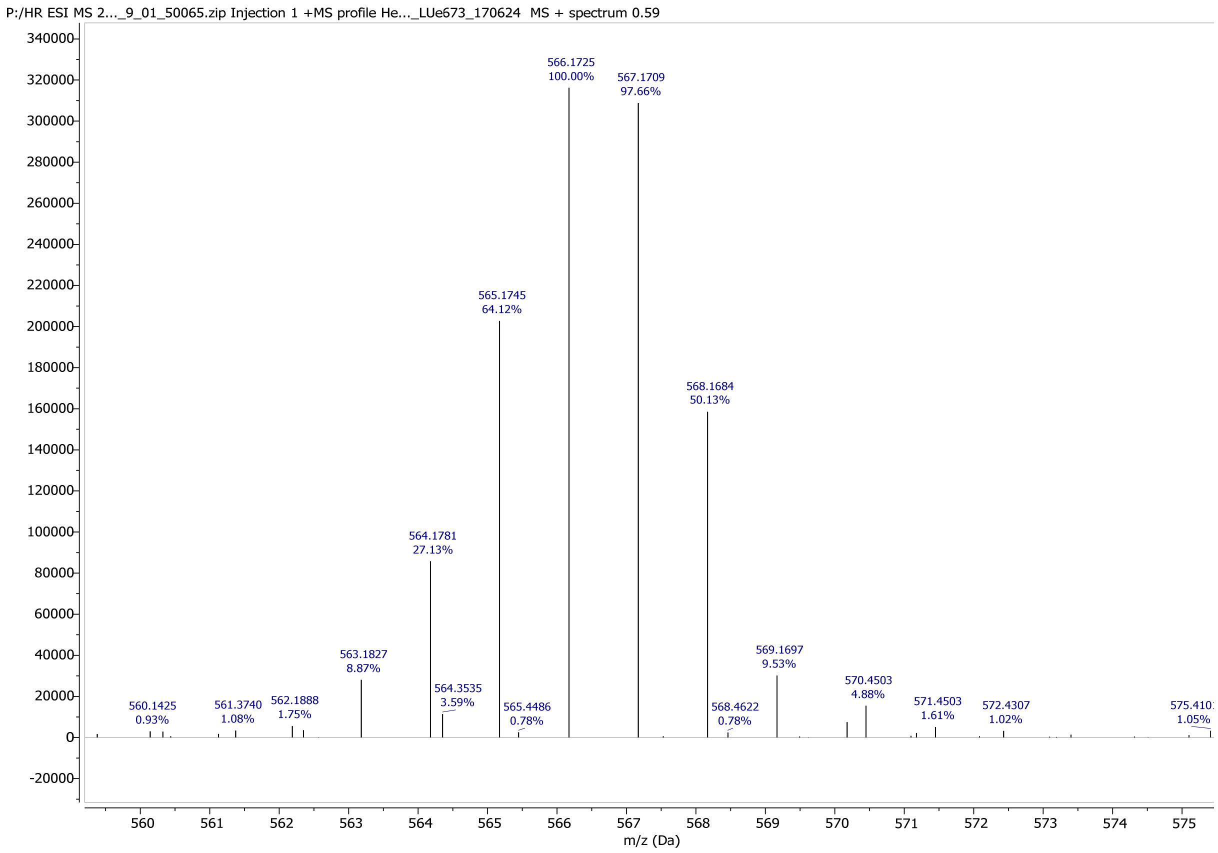

Figure S67. HRMS (ESI+) of compound $\mathbf{3}_{\boldsymbol{p}}$ in CH_3CN.

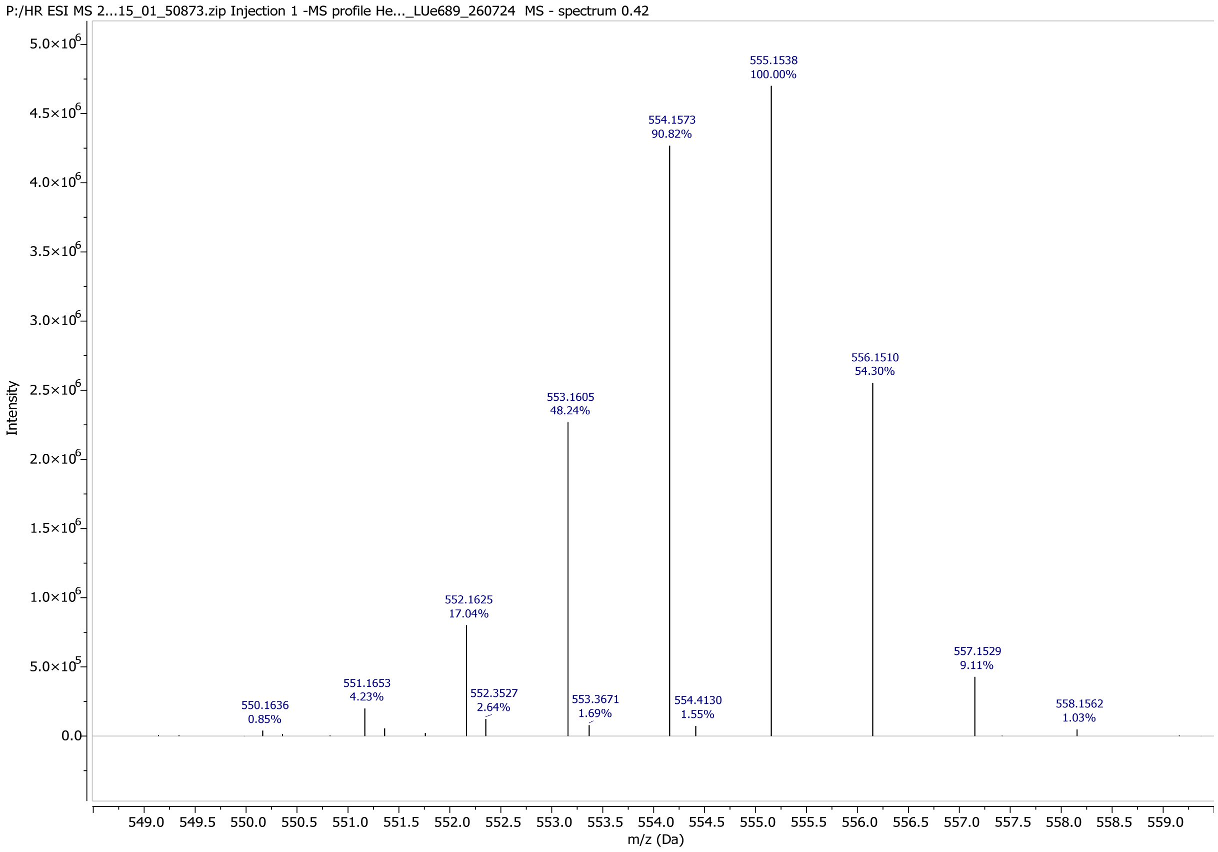

Figure S68. HRMS (ESI–) of compound **5** in MeOH.

4 Determination of HPLC Purity of Compounds $2_{o,m,p}$, $3_{o,m,p}$, 4, and 5

The HPLC purity was determined using HPLC-UV-MS with an RP column. The *closo*-carborane derivatives $\mathbf{2}_{o,m,p}$ and $\mathbf{3}_{o,m,p}$ were dissolved in CH_3CN, the *nido*-carborane derivatives **4** and **5** in MeOH. All compounds had a purity >95%. The double peaks or shoulders visible for the signals of the compounds are related to the column that has been used and not to the compound, since variations in gradient system could not resolve the double-peaks or shoulders. Broad signals have been observed in case of *nido*-carborane derivatives.

Compound $\mathbf{2}_{o}$

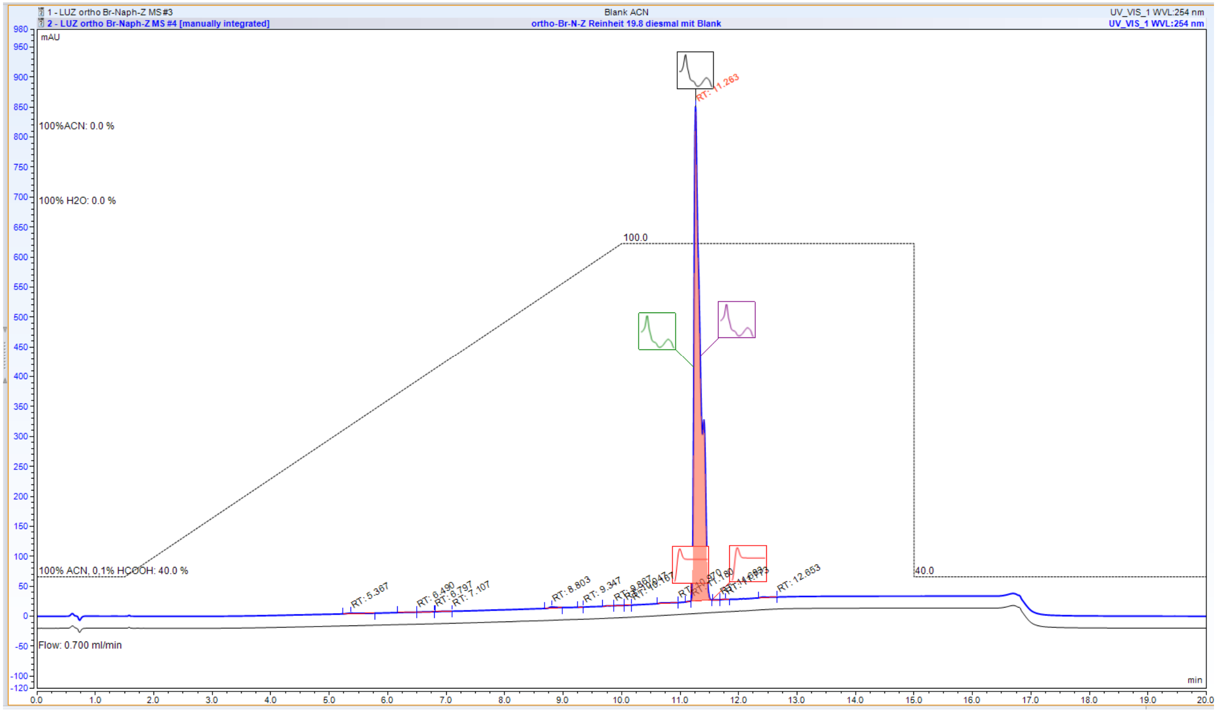

Figure S69. RP-HPLC chromatogram of blank (CH_3CN) and compound $\mathbf{2}_{o}$, retention time: 11.3 min.

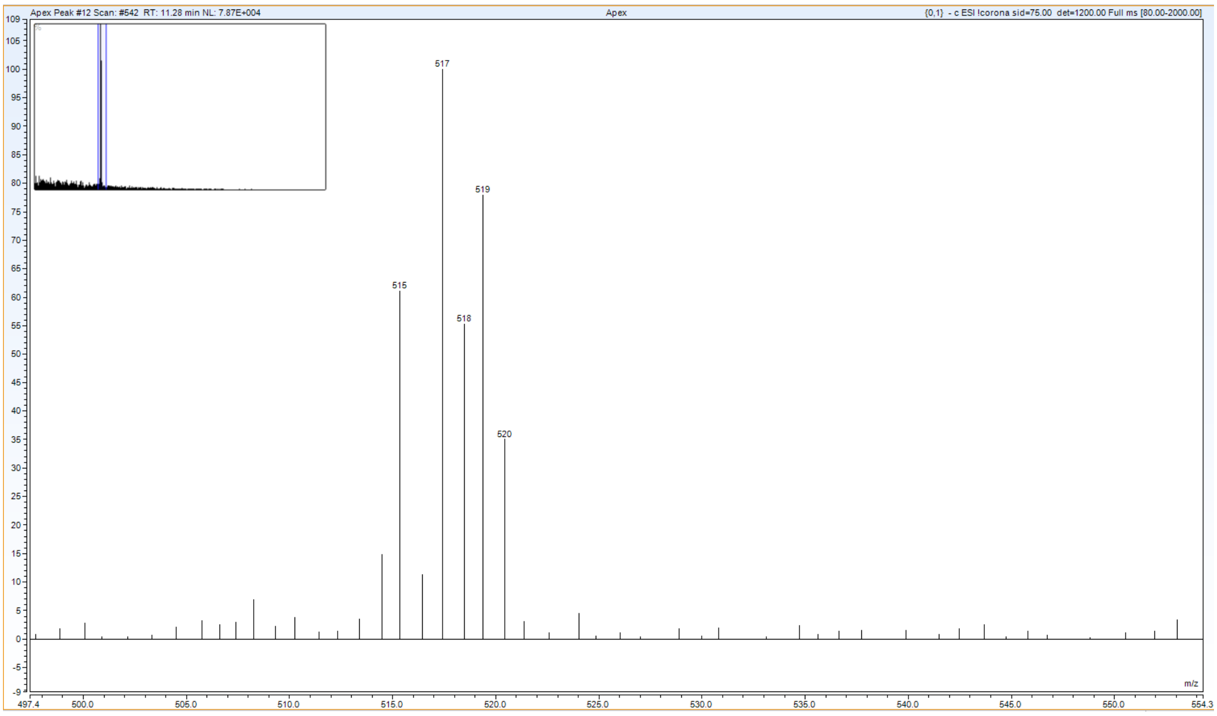

Figure S70. MS(-) of compound **2*o***, retention time: 11.3 min.

Compound **2*m***

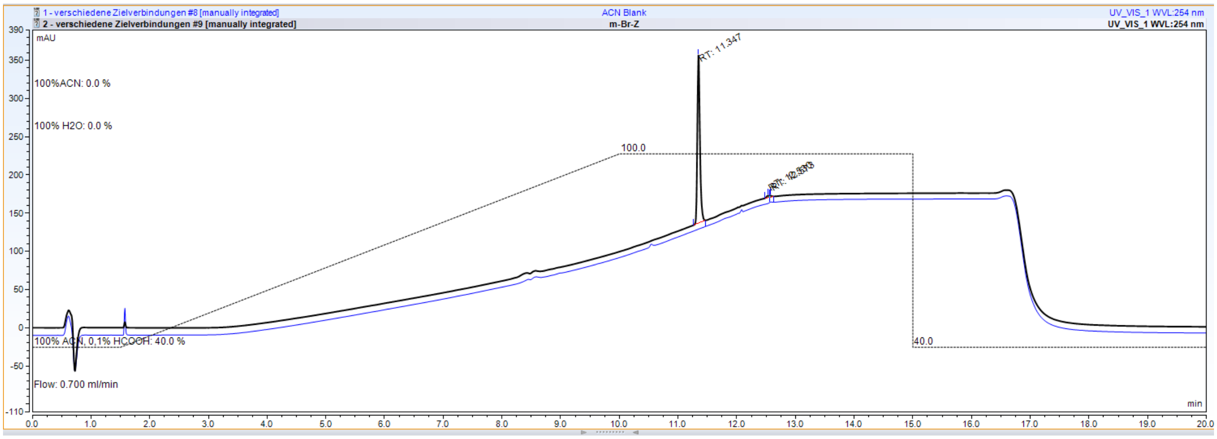

Figure S71. RP-HPLC chromatogram of blank (CH_3CN) and compound **2*m***, retention time: 11.3 min.

Compound 2_p

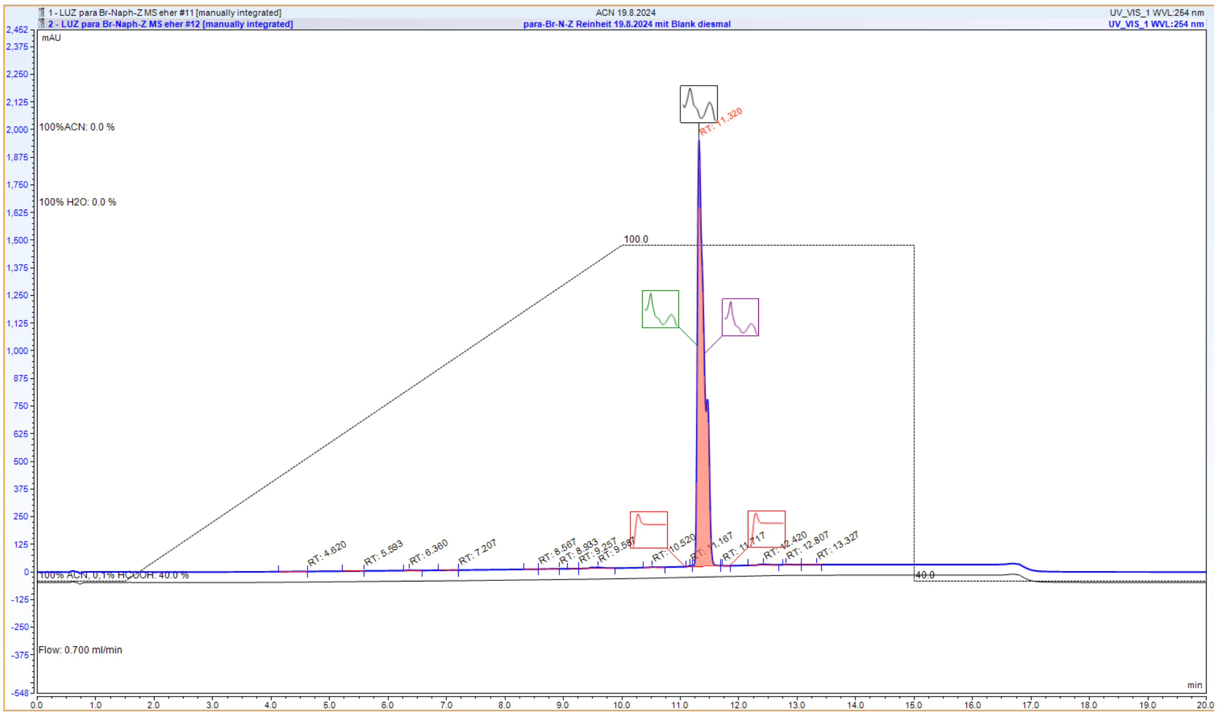

Figure S72. RP-HPLC chromatogram of blank (CH_3CN) and compound $\mathbf{2}_p$, retention time: 11.3 min.

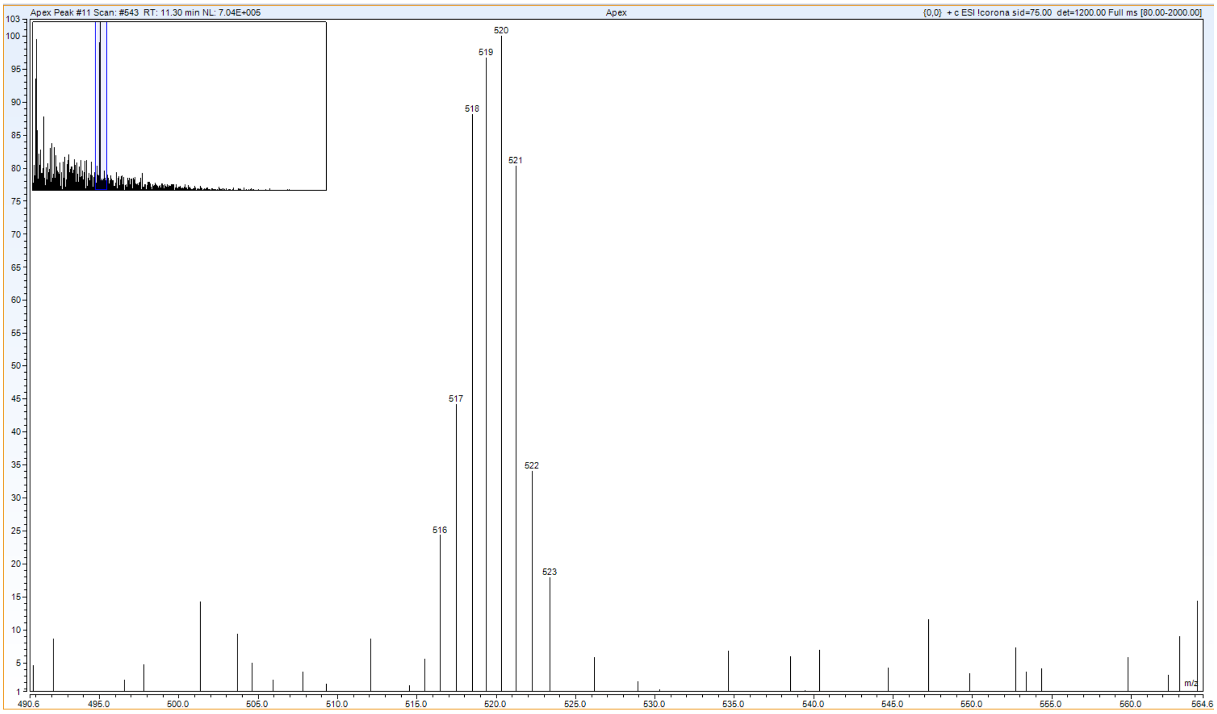

Figure S73. MS(+) of compound $\mathbf{2}_p$, retention time: 11.3 min.

Compound 3_o

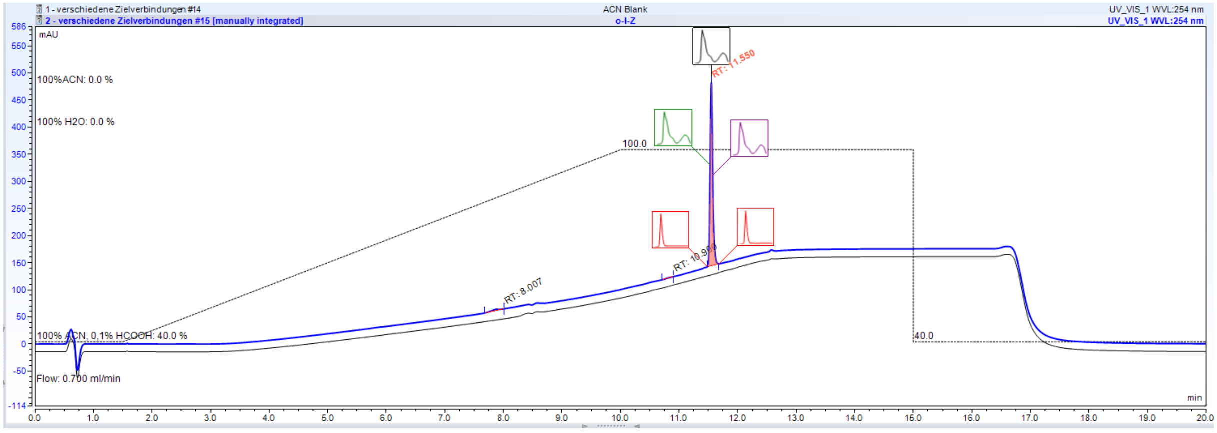

Figure S74. RP-HPLC chromatogram of blank (CH_3CN) and compound $\mathbf{3_o}$, retention time: 11.6 min.

Compound 3_m

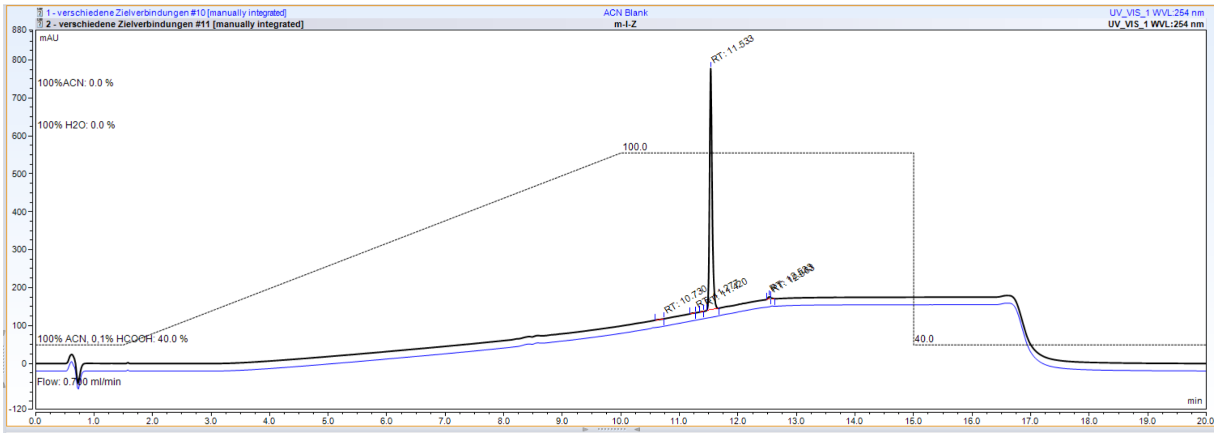

Figure S75. RP-HPLC chromatogram of blank (CH_3CN) and compound $\mathbf{3_m}$, retention time: 11.5 min.

Compound 3_p

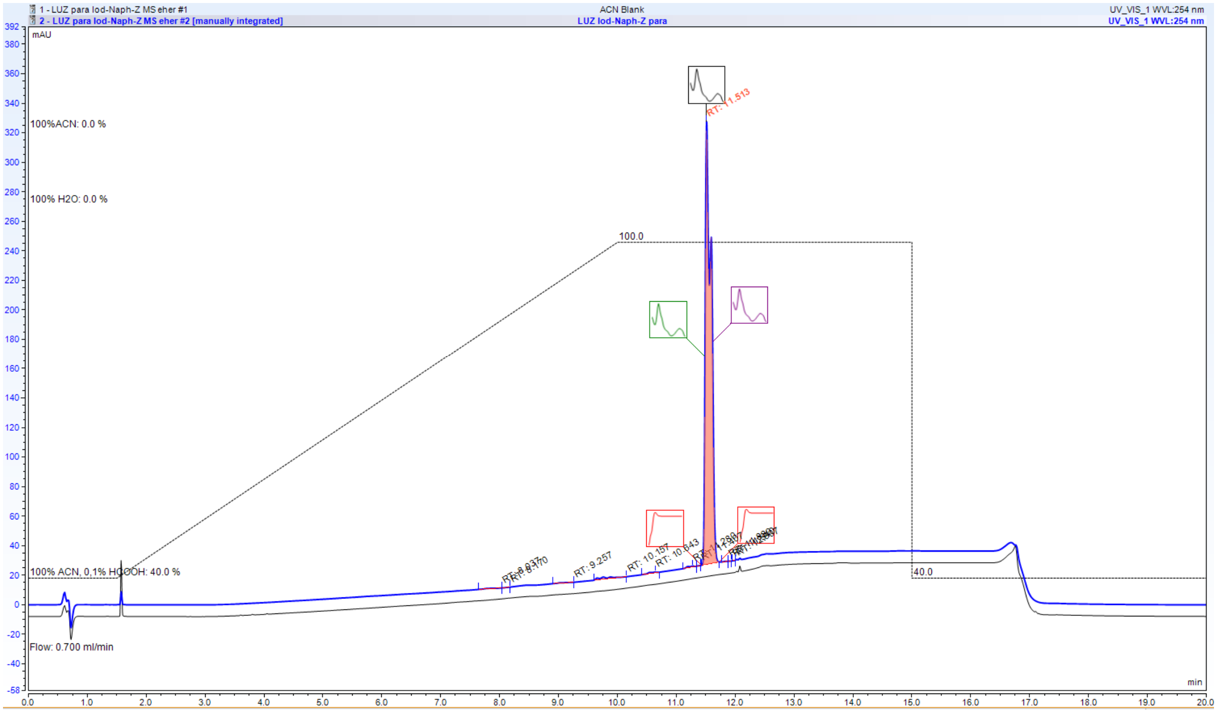

Figure S76. RP-HPLC chromatogram of blank (CH_3CN) and compound **3_p**, retention time: 11.5 min.

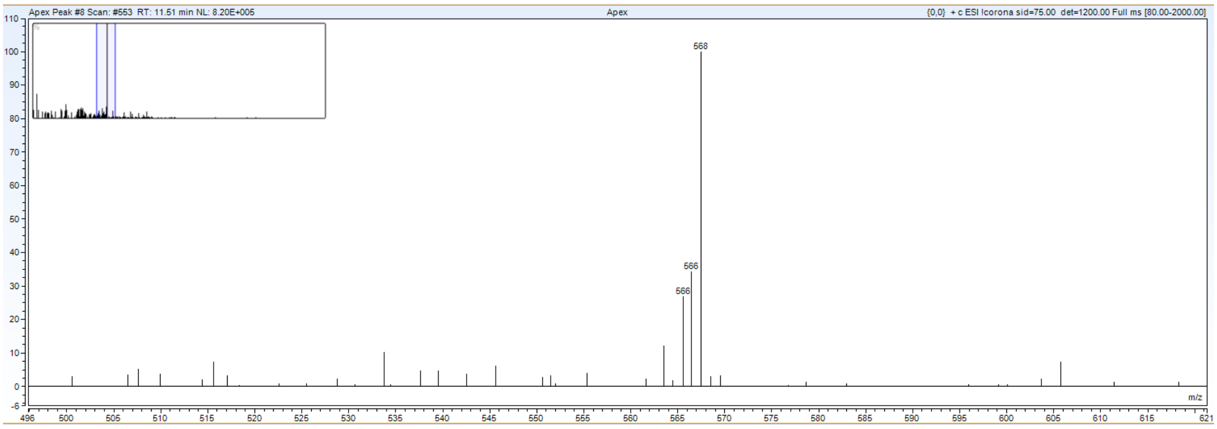

Figure S77. MS(+) of compound **3_p**, retention time: 11.5 min.

Compound **4**

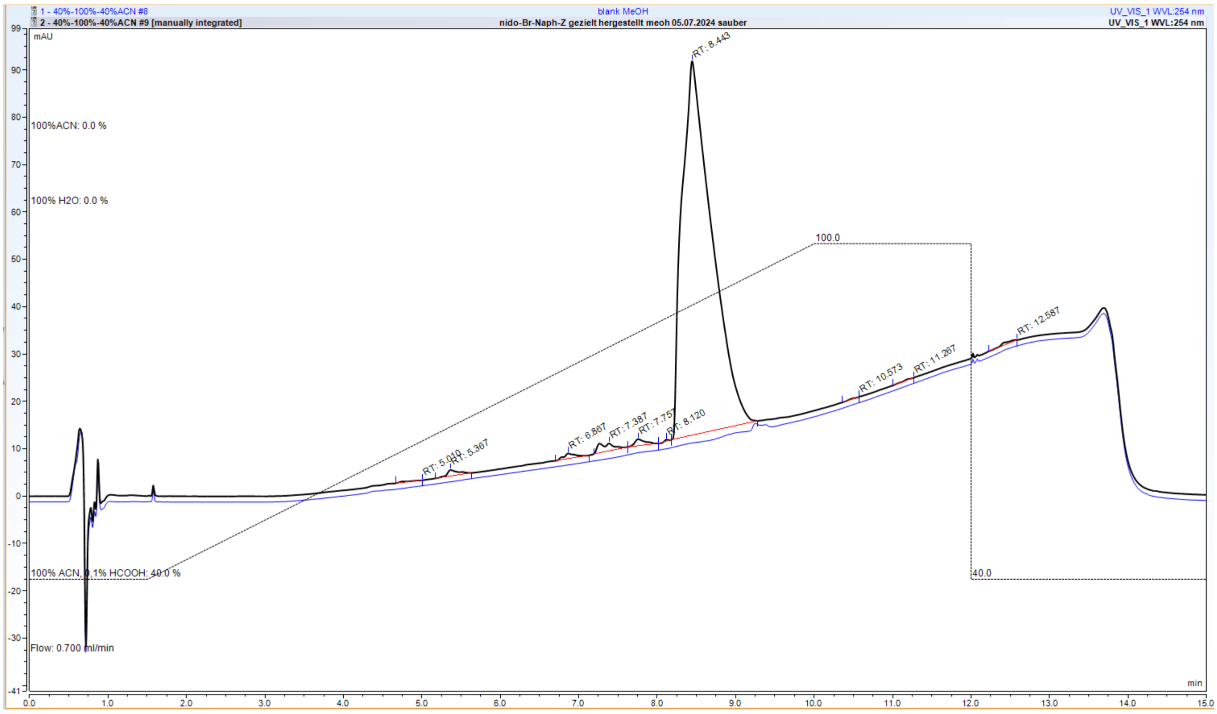

Figure S78. RP-HPLC chromatogram of blank (MeOH) and compound **4**, retention time: 8.4 min.

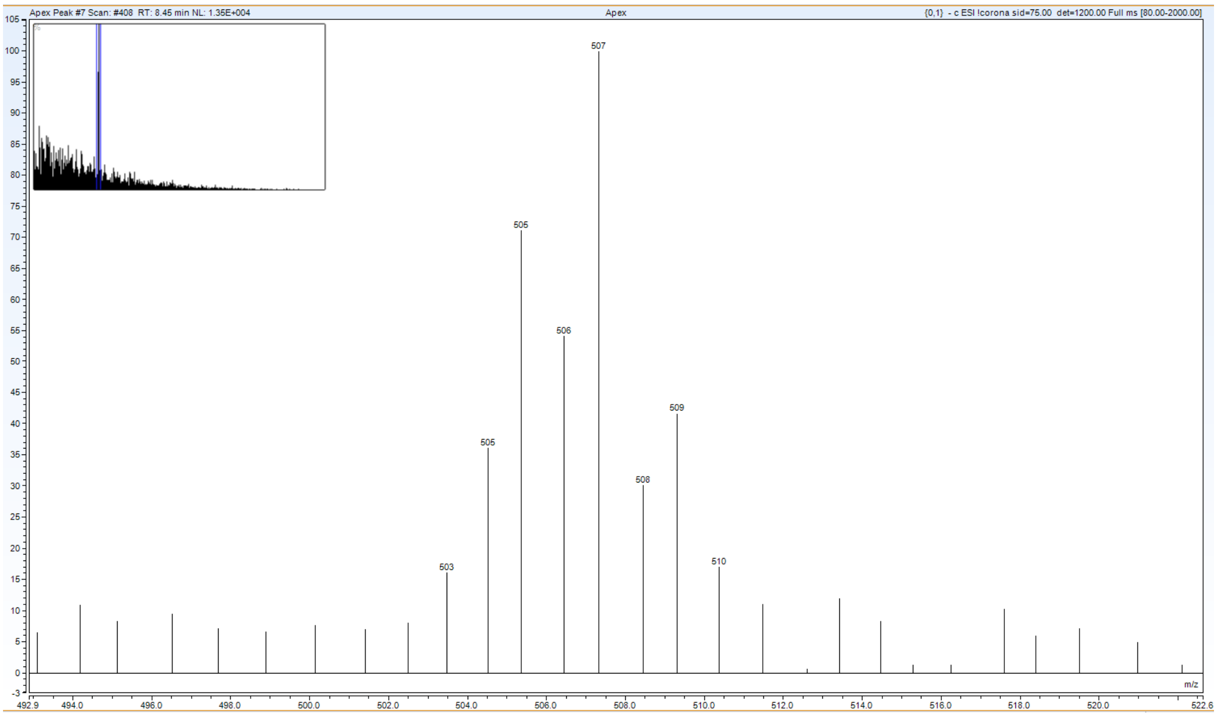

Figure S79. MS(-) of compound **4**, retention time: 8.45 min.

Compound **5**

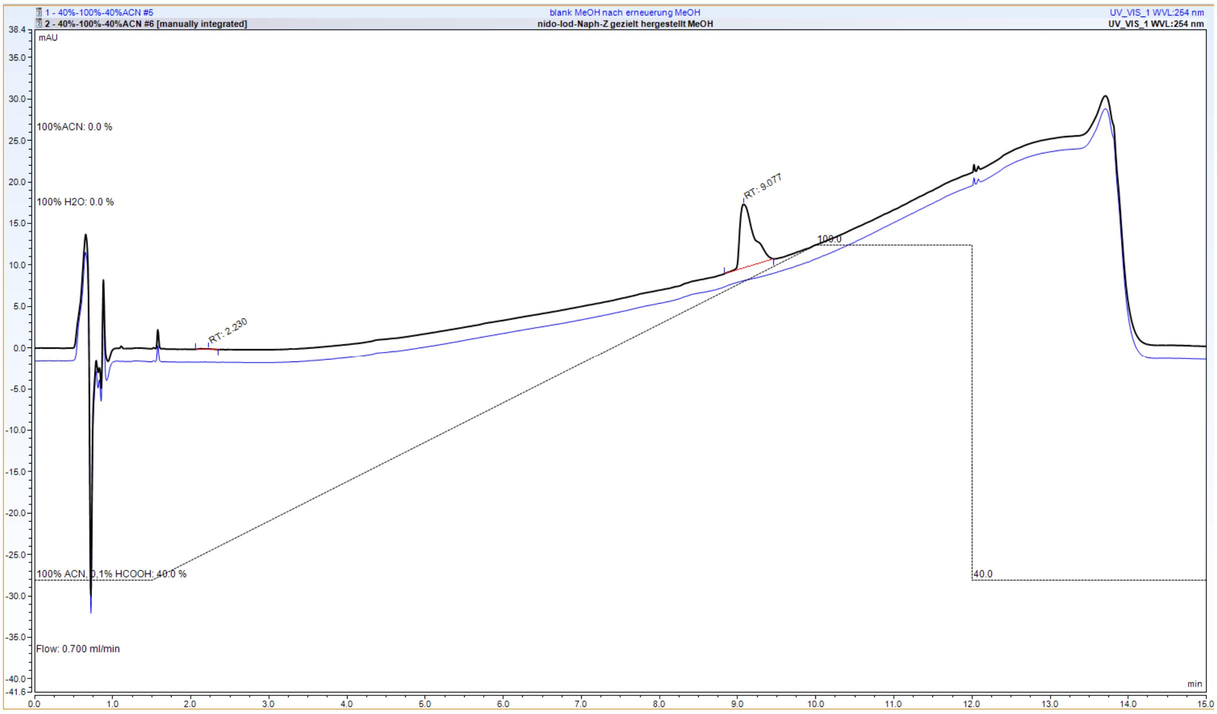

Figure S80. RP-HPLC chromatogram of blank (MeOH) and compound **5**, retention time: 9.1 min.

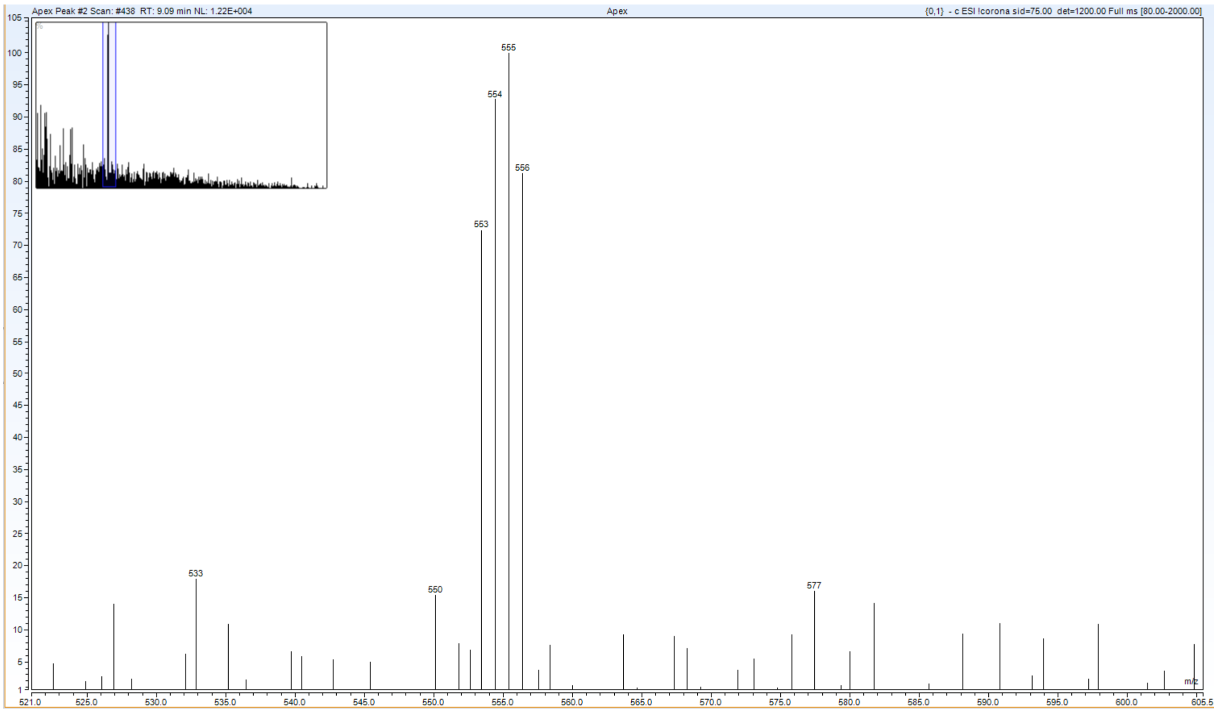

Figure S81. MS(-) of compound **5**, retention time: 9.1 min.

5 Determination of the Stability of Compounds $2_{o,m,p}$, $3_{o,m,p}$, 4, and 5 by HPLC

The stability measurements have been performed with an HPLC-UV-MS on an RP column. Blank samples of pure DMSO/H_20 (1:1, *v*/*v*) have been measured prior to the target compounds. The target compounds have been dissolved in DMSO/H_2O (1:1, *v*/*v*) for stability determination. The measurements have been started directly after the addition of the water. The double peaks or shoulders of the signals of the compound are related to the column that has been used. Broad signals have been observed in case of the *nido*-carborane derivatives.

Compound **2*o***

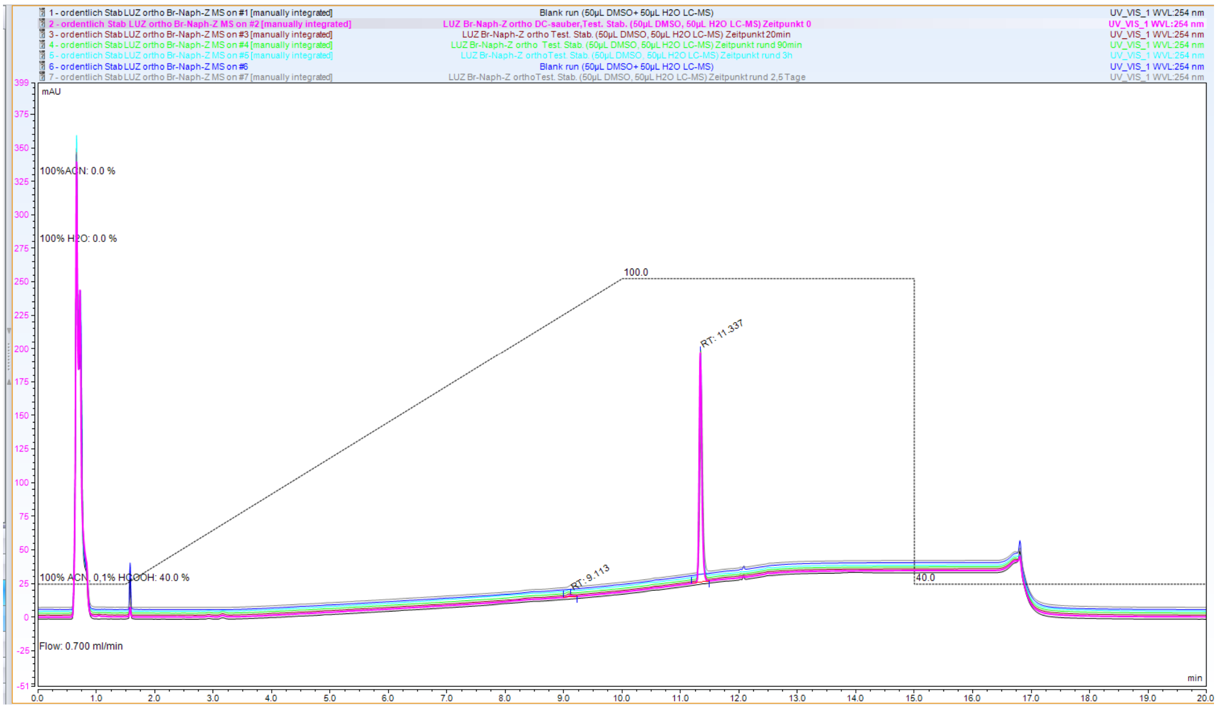

Figure S82. RP-HPLC chromatograms of blank (DMSO/H_2O, black and dark blue) and compound **2*o***, retention time: 11.4 min, purity after 2.5 d: >98%.

Compound **2***m*

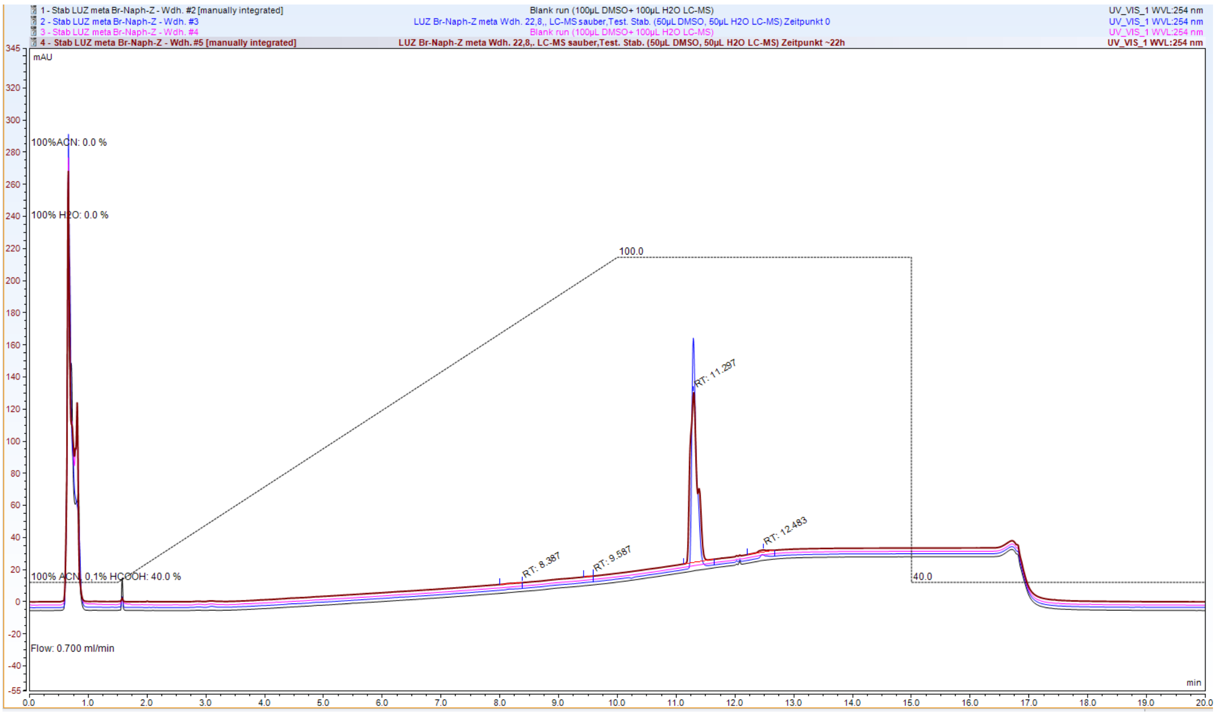

Figure S83. RP-HPLC chromatograms of blank (DMSO/H_2O, black, pink) and compound **2***m*, retention time: 11.3 min, purity after 1 d: ~98%.

Compound **2***p*

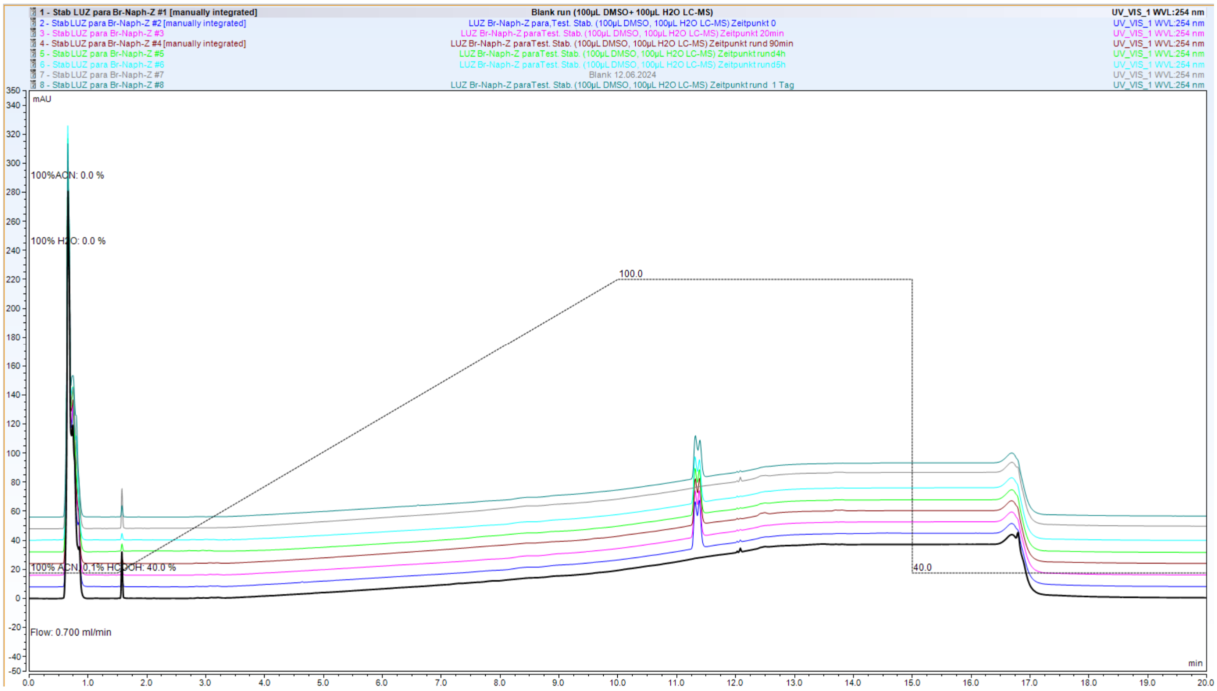

Figure S84. RP-HPLC chromatograms of blank (DMSO/H_2O, black, grey) and compound **2***p*, retention time: 11.4 min

Compound $\mathbf{3_o}$

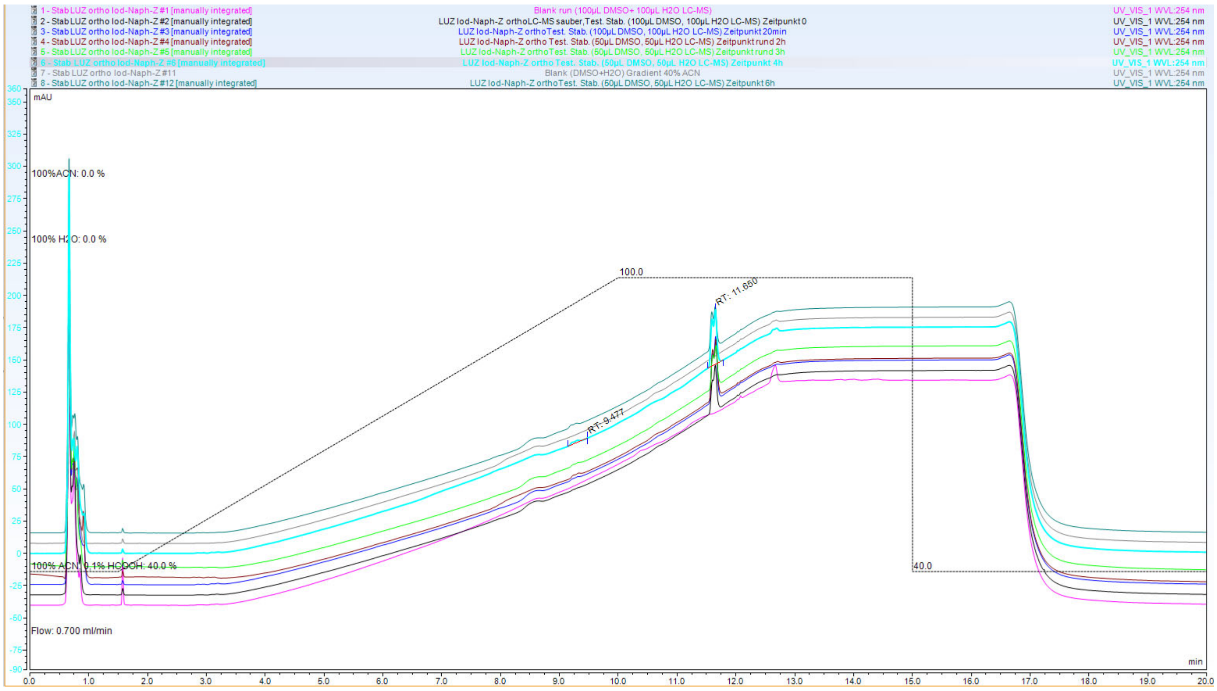

Figure S85. RP-HPLC chromatograms of blank (DMSO/H_2O, pink, grey) and compound $\mathbf{3_o}$, retention time: 11.7 min, purity after 6 h: ~93%

Compound $\mathbf{3_m}$

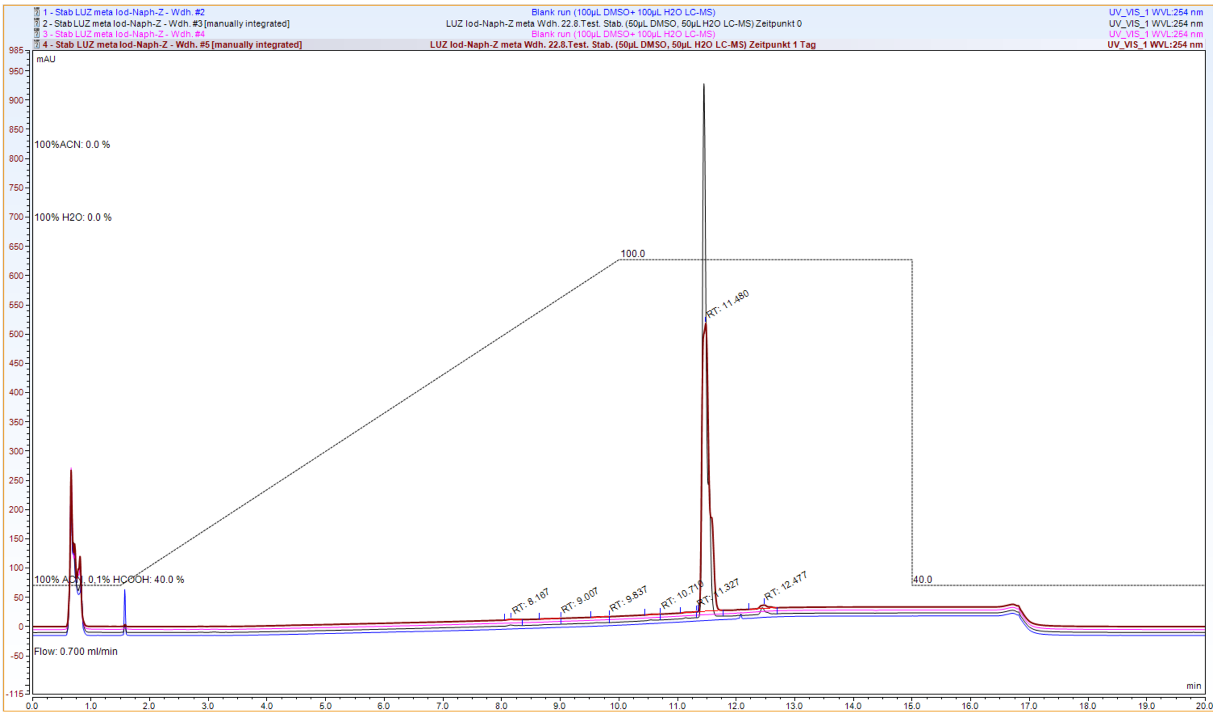

Figure S86. RP-HPLC chromatograms of blank (DMSO/H_2O, pink, blue) and compound $\mathbf{3_m}$, retention time: 11.5 min, purity after 1 d: ~98%

Compound 3_p

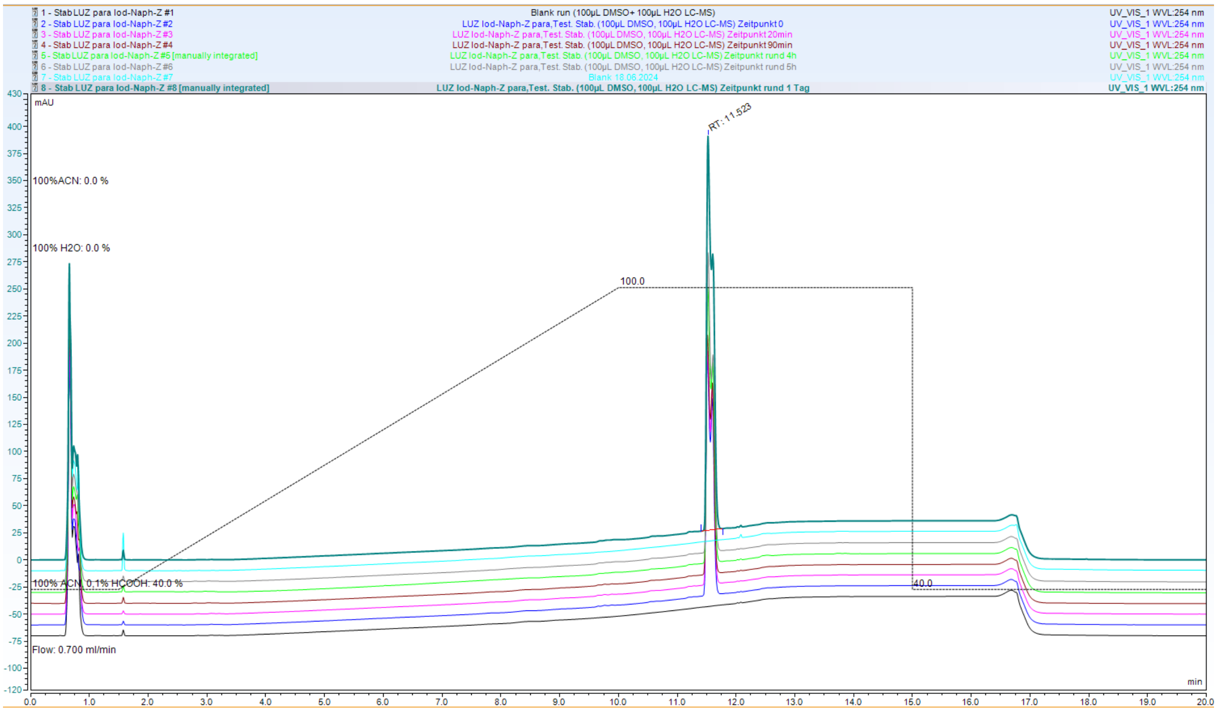

Figure S87. RP-HPLC chromatograms of blank (DMSO/H_2O, black, light blue) and compound $\mathbf{3}_p$, retention time: 11.5 min, purity after 1 d: >98%

Compound 4

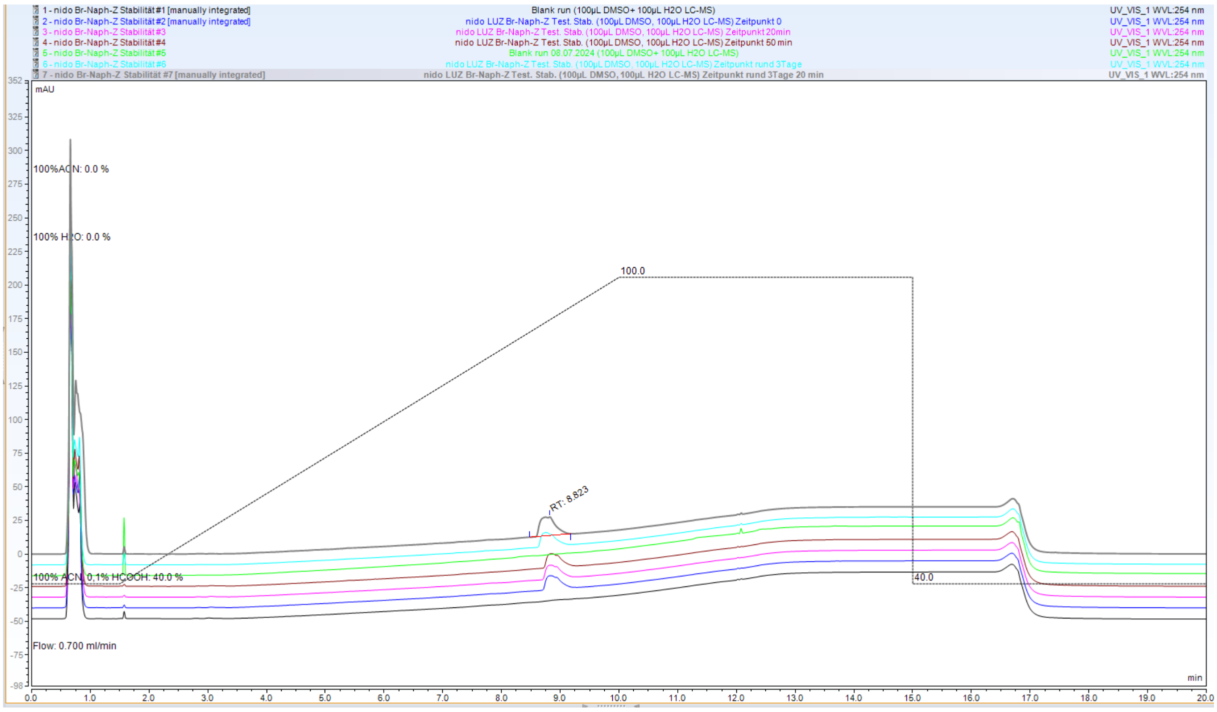

Figure S88. RP-HPLC chromatograms of blank (DMSO/H_2O, black, green) and compound **4**, retention time: 8.8 min, purity after 3 d: ~98%

Compound **5**

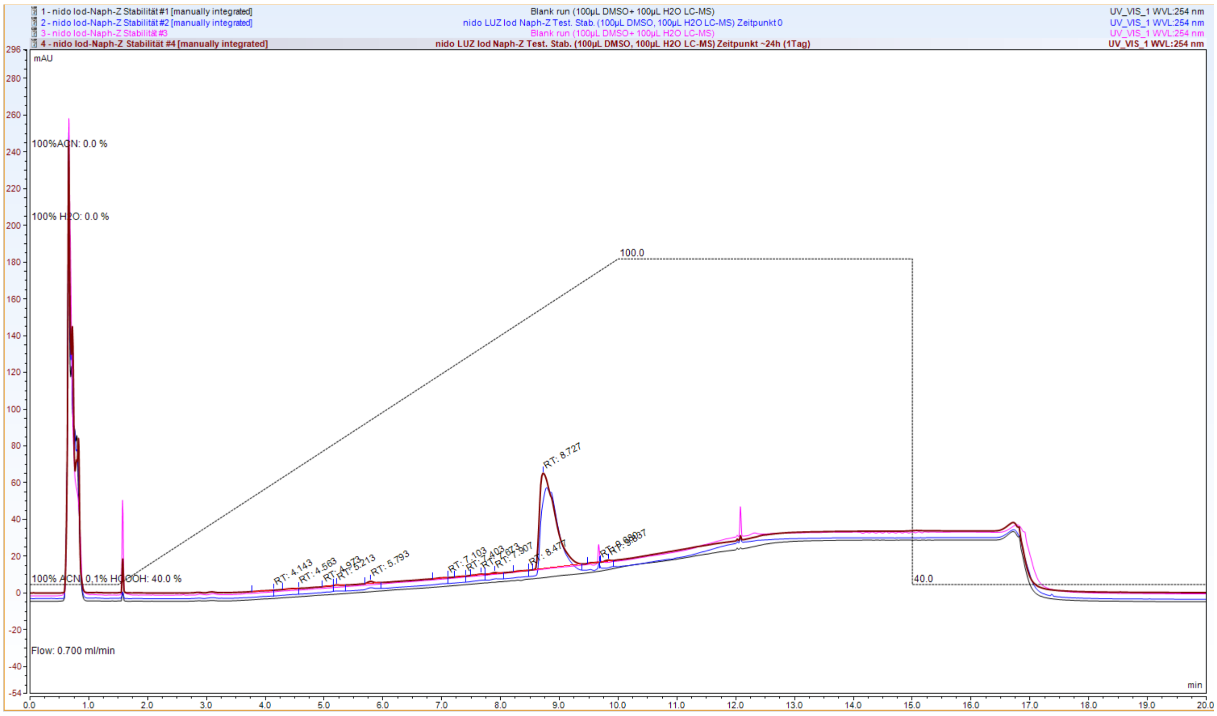

Figure S89. RP-HPLC chromatograms of blank (DMSO/H_2O, black, pink) and compound **5**, retention time: 8.7 min, purity after 1 d: >97%

6 X-ray Crystallography Data of Compounds 2_o, 2_p, 3_o, and 3_m

Table S1. Fundamental structure parameters of $\mathbf{2}_o$, $\mathbf{2}_p$, $\mathbf{3}_o$, $\mathbf{3}_m$ at 190(2) K.

**Compound**	$\mathbf{2}_o$	$\mathbf{2}_p$	$\mathbf{3}_o$	$\mathbf{3}_m$
Empirical formula	$C_{18}H_{21}B_{10}BrFN_3O_2$	$C_{18}H_{21}B_{10}BrFN_3O_2$	$C_{18}H_{21}B_{10}FIN_3O_2$	$C_{18}H_{21}B_{10}FIN_3O_2$
Formula weight	518.39	518.39	565.38	565.38
Temperature [K]	190(2)	190(2)	190(2)	190(2)
Wavelength [pm]	71.073	71.073	71.073	71.073
Crystal system	Triclinic	Triclinic	Triclinic	Triclinic
Space group	$P\overline{1}$	$P\overline{1}$	$P\overline{1}$	$P\overline{1}$
Unit cell dimensions				
a [pm]	717.95(1)	716.05(2)	729.17(2)	729.57(2)
b [pm]	1262.93(3)	1259.81(5)	1261.01(4)	1251.29(4)
c [pm]	1441.12(3)	1438.06(5)	1438.32(4)	1435.06(4)
α [deg]	114.074(2)	114.635(3)	113.052(3)	112.654(3)
β [deg]	94.818(2)	95.020(2)	95.013(2)	94.816(2)
γ [deg]	99.527(2)	99.560(3)	100.414(3)	99.656(3)
Volume [nm^3]	1.15981(4)	1.14489(7)	1.17845(7)	1.17587(6)
Z	2	2	2	2
$\rho_{(calculated)}$ [Mg/m^3]	1.484	1.504	1.593	1.597
μ [mm^{-1}]	1.804	1.828	1.390	1.393
F(000)	520	520	556	556
Crystal size [mm^3]	0.40 · 0.26 · 0.20	0.40 · 0.26 · 0.19	0.28 · 0.24 · 0.09	0.32 · 0.13 · 0.09
Θ_{Min} / Θ_{Max} [deg]	2.861 / 30.330	2.877 / 30.269	2.838 / 32.296	2.826 / 32.382
Index ranges	-10 ≤ h ≤ 10<br>-17 ≤ k ≤ 17<br>-20 ≤ l ≤ 19	-9 ≤ h ≤ 10<br>-17 ≤ k ≤ 17<br>-20 ≤ l ≤ 20	-10 ≤ h ≤ 10<br>-18 ≤ k ≤ 18<br>-20 ≤ l ≤ 21	-10 ≤ h ≤ 10<br>-18 ≤ k ≤ 18<br>-21 ≤ l ≤ 21
Reflections collected	30819	22051	21855	25123
Indp. reflections (R_{int})	6479 (0.0315)	6278 (0.0291)	7700 (0.0262)	7754 (0.0276)
Completeness (Θ_{Max}) [deg]	99.9 % (28.285)	99.9 % (28.285)	99.9 % (30.510)	100.0 % (30.510)
T_{Max} / T_{Min}	1.00000 / 0.93618	1.00000 / 0.88629	1.00000 / 0.98898	1.00000 / 0.91640
Restraints / [illegible]	0 / 400	0 / 400	0 / 400	0 / 400
Gof on F^2	1.077	1.030	1.019	1.041
R1 / wR2 (I>2σ(I))	0.0343 / 0.0783	0.0322 / 0.0758	0.0298 / 0.0631	0.0300 / 0.0625
R1 / wR2 (all data)	0.0537 / 0.0867	0.0438 / 0.0819	0.0409 / 0.0679	0.0414 / 0.0675
Residual electron density [e·$Å^{-3}$]	0.324 / -0.416	0.416 / -0.534	0.462 / -0.556	0.616 / -0.622
Comments	†1	-	-	-
CCDC No[103]	2432537	2432538	2432539	2432540

†1: The crystals always tend to crack at temperatures below 180 K. For this reason, all samples had been recorded at 190 K.

Table S2. Fundamental structure parameters of $\mathbf{2_o}$, $\mathbf{2_p}$ at 297(2) K.

**Compound**	$\mathbf{2}_o$	$\mathbf{2}_p$
Empirical formula	$C_{18}H_{21}B_{10}BrFN_3O_2$	$C_{18}H_{21}B_{10}BrFN_3O_2$
Formula weight	518.39	518.39
Temperature [K]	297(2)	297(2)
Wavelength [pm]	71.073	71.073
Crystal system	Triclinic	Triclinic
Space group	$P\overline{1}$	$P\overline{1}$
Unit cell dimensions		
a [pm]	715.15(2)	716.53(1)
b [pm]	1268.90(6)	1267.64(3)
c [pm]	1457.16(4)	1447.18(3)
α [deg]	113.502(3)	114.284(2)
β [deg]	95.248(2)	95.478(2)
γ [deg]	98.669(3)	98.721(2)
Volume [nm^3]	1.18189(8)	1.16601(4)
Z	2	2
$\rho_{(calculated)}$ [Mg/m^3]	1.457	1.476
μ [mm^{-1}]	1.770	1.794
F(000)	520	520
Crystal size [mm^3]	0.35 · 0.24 · 0.16	0.41 · 0.27 · 0.17
Θ_{Min} / Θ_{Max} [deg]	2.812/ 29.197	2.856/ 28.703
Index ranges	$-9 \leq h \leq 9$<br>$-16 \leq k \leq 17$<br>$-19 \leq l \leq 19$	$-9 \leq h \leq 9$<br>$-16 \leq k \leq 16$<br>$-19 \leq l \leq 19$
Reflections collected	27843	27492
Indp. reflections (R_{int})	5745 (0.0292)	5440 (0.0278)
Completeness (Θ_{Max}) [deg]	99.9 % (26.375)	99.9 % (26.375)
T_{Max} / T_{Min}	1.00000 / 0.94443	1.00000 / 0.91888
Restraints / parameters	421 / 429	0 / 400
Gof on F^2	1.017	1.044
R1 / wR2 (I>2σ(I))	0.0373 / 0.0839	0.0375 / 0.0904
R1 / wR2 (all data)	0.0685 / 0.0973	0.0565 / 0.1004
Residual electron density [e·Å^{-3}]	0.208 / -0.510	0.474 / -0.490
Comments	†[2]	-
CCDC No[103]	2432541	2432542

†[2]: The *ortho*-carborane unit is disordered on two positions with a ratio of 0.67(1):0.33(1).

7 Docking Studies of Compounds $2_{o,m,p}$, $3_{o,m,p}$, 4, and 5

Molecular docking was performed using AutoDockTools4[104] with the Lamarckian Genetic Algorithm.[105] The force-field parameters for boron atoms were manually added to the AutoDockTools4 parameter file.

The protein structure (PDB ID: 5ZTY)[9] was obtained from the Protein Data Bank (PDB) and originally contained the ligand *N*-(adamantan-1-yl)-1-(5-hydroxypentyl)-4-methyl-5-phenyl-1*H*-pyrazole-3-carboxamide. The docking site was selected based on the position of this ligand within the binding pocket. Prior to docking, the ligand and water molecules were removed, and the protein structure was protonated using the Reduce software.[106]

For docking, water molecules were eliminated, and non-polar hydrogen atoms were merged. The docking grid box was set with the parameters bellow:

58 x 44 x 54 center at (7.24, 1.919, -60.355) for $\mathbf{2}_{o}$, $\mathbf{2}_{m}$, $\mathbf{2}_{p}$, **4**,

9.441 0.12 -59.514 center at (8.982 1.344 -54.339) for $\mathbf{3}_{o}$, $\mathbf{3}_{m}$, $\mathbf{3}_{p}$, **5**,

aligning with the ligand-binding domain (LBD).

The following docking parameters were used:

- Number of hybrid GA-LS runs: 100
- Population size: 150
- Maximum number of energy evaluations: 25,000,000
- Top individuals surviving to the next generation: 1
- Gene mutation rate: 0.02
- Crossover rate: 0.8
- Mean of Cauchy distribution for gene mutation: 0.0
- Variance of Cauchy distribution for gene mutation: 1.0

8 Chemical Structures of WIN55212-2 and SR141716A

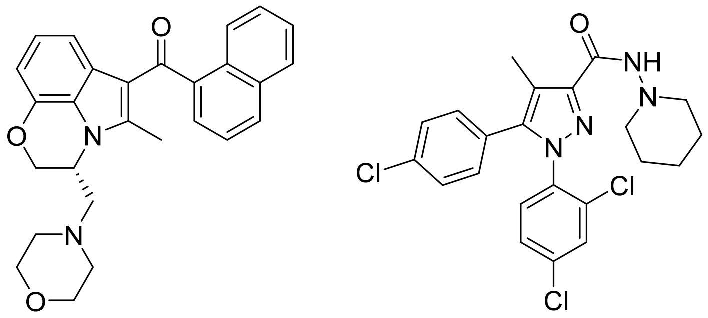

WIN55212-2 **SR141716A**

Figure S90. Chemical Structures of CB_2R agonist **WIN55212-2** and CB_1R antagonist/inverse agonist **SR141716A**.

[1] V. Lucchesi, D.P. Hurst, D.M. Shore, S. Bertini, B.M. Ehrmann, M. Allarà, L. Lawrence, A. Ligresti, F. Minutolo, G. Saccomanni, H. Sharir, M. Macchia, V. Di Marzo, M.E. Abood, P.H. Reggio, C. Manera, *J. Med. Chem.* **2014**, *57,* 8777–8791.

[9] X. Li, T. Hua, K. Vemuri, J.-H. Ho, Y. Wu, L. Wu, P. Popov, O. Benchama, N. Zvonok, K. Locke, L. Qu, G.W. Han, M.R. Iyer, R. Cinar, N.J. Coffey, J. Wang, M. Wu, V. Katritch, S. Zhao, G. Kunos, L.M. Bohn, A. Makriyannis, R.C. Stevens, Z.-J. Liu, *Cell* **2019**, *176,* 459-467.e13.

[89] H. Suzuki, *Org. Synth.* **1971**, *51,* 94.

[90] L. Fader, P.L. Beaulieu, M. Bailey, F. Bilodeau, R. Carson, A. Giroux, C. Godbout, O. Hucke, M.A. Joly, M. Leblanc, O. Lepage, B. Moreau, J. Naud, M. Poirier, E. Villemure, CA2873882 (A1), **2013**.

[100] Y. Nie, Y. Wang, J. Miao, Y. Li, Z. Zhang, *J. Organomet. Chem.* **2015**, *798,* 182–188.

[101] M. Scholz, A.L. Blobaum, L.J. Marnett, E. Hey-Hawkins, *Bioorg. Med. Chem.* **2012**, *20,* 4830–4837.

[102] S. Choi, Y. Byun, *J. Organomet. Chem.* **2013**, *733,* 49–52.

[103] Deposition numbers 2432537 (for $\mathbf{2}_{o}$ at 190(2) K), 2432538 (for $\mathbf{2}_{p}$ at 190(2) K), 2432539 (for $\mathbf{3}_{o}$ at 190(2) K) 2432540 (for $\mathbf{3}_{m}$ at 190(2) K), 2432541 (for $\mathbf{2}_{o}$ at 297(2) K) and 2432542 (for $\mathbf{2}_{p}$ at 297(2) K) contain the supplementary crystallographic data for this paper. These data are provided free of charge by the joint Cambridge Crystallographic Data Centre and Fachinformationszentrum Karlsruhe Access Structures service (http://www.ccdc.cam.ac.uk/structures).

[104] G.M. Morris, R. Huey, W. Lindstrom, M.F. Sanner, R.K. Belew, D.S. Goodsell, A.J. Olson, *J. Comput. Chem.* **2009**, *30,* 2785–2791.

[105] G.M. Morris, D.S. Goodsell, R.S. Halliday, R. Huey, W.E. Hart, R.K. Belew, A.J. Olson, *J. Comput. Chem.* **1998**, *19,* 1639–1662.

[106] J. Word, S.C. Lovell, J.S. Richardson, D.C. Richardson, *J. Mol. Biol.* **1999**, *285,* 1735–1747.
